# PRDM16 Inhibits Cell Proliferation and Migration via Epithelial-to-Mesenchymal Transition by Directly Targeting Pyruvate Carboxylase in Papillary Thyroid Cancer

**DOI:** 10.3389/fcell.2021.723777

**Published:** 2021-11-02

**Authors:** Wan-Lin Liu, Qing Guan, Duo Wen, Ben Ma, Wei-Bo Xu, Jia-Qian Hu, Wen-Jun Wei, Duan-Shu Li, Yu Wang, Jun Xiang, Tian Liao, Qing-Hai Ji

**Affiliations:** ^1^Department of Head and Neck Surgery, Shanghai Cancer Center, Fudan University, Shanghai, China; ^2^Department of Oncology, Shanghai Medical College, Fudan University, Shanghai, China

**Keywords:** papillary thyroid cancer, PRDM16, pyruvate carboxylase, epithelial to mesenchymal transition, metastasis

## Abstract

PRDM16 (known as MEL1), a member of the PR domain zinc finger family, has been implicated in multiple biological processes, including cancers. It is not clear yet whether PRDM16 is involved in tumor progress of papillary thyroid cancer (PTC). We identified the PRDM16 expression level in PTC tissues by qRT-PCR and analyzed its relationship with clinical characteristics in both Fudan University Shanghai Cancer Center (FUSCC) and TCGA cohorts. We tested the function of PRDM16 in PTC cells both *in vivo* and *in vitro*. We found a direct downstream target of PRDM16, pyruvate carboxylase (PC), by RNA-sequencing, rescue experiments, luciferase assay, and chromatin immunoprecipitation assay. PRDM16 was downregulated in papillary thyroid cancer tissues and was significantly related with lymph node metastases and extrathyroidal extension in both FUSCC and TCGA cohorts. Overexpression of PRDM16 could attenuate proliferation and migration of PTC cells via inhibiting the epithelial-to-mesenchymal transition process. PC was upregulated in papillary thyroid cancer tissues. Knockdown of PC could inhibit proliferation and migration in TPC-1 and K1 cells. The repression effect on cell proliferation and migration from PRDM16 was PC dependent. PRDM16 could directly bind to the PC promoter and inhibit its expression at the transcription level. Moreover, the mRNA expression level of PRDM16 and PC was negatively related in human PTC tissues. In conclusion, PRDM16 exhibited an antitumor effect and EMT inhibition function in PTC by directly binding with the PC promoter. PRDM16 may be a novel therapeutic target in papillary thyroid cancer.

## Introduction

Thyroid cancer is the most common type of endocrine malignancy. Since the 1970s, its incidence has increased threefold over the past three decades all over the world (Chen et al., 2009). Based on recent data, thyroid cancer is the fifth most common cancer in women in the United States (Bray et al., 2018). Papillary thyroid carcinoma (PTC) comprises 80% of all thyroid cancer, named for the papillary histological architecture (Cancer Genome Atlas Research Network, 2014). Worldwide trends in thyroid cancer incidence have been largely driven by an increase in PTC, followed by follicular, medullar, and anaplastic histological subtypes (Kitahara and Sosa, 2016). Though the prognosis of papillary thyroid cancer is usually excellent with a 5-year survival rate exceeding 95% (Hay et al., 2002) and a 10-year survival rate of more than 90% (Leboulleux et al., 2005), about 20% of PTC patients still develop recurrent disease, including local cervical recurrences and distant metastases (Carling and Udelsman, 2014), which can be incurable and fatal (Mazzaferri and Massoll, 2002). Prognostic factors of recurrence for PTC include age at diagnosis, histological subtypes, extrathyroidal extension (ETE), lymph node metastases (LNM), and tumor-node-metastasis (TNM) stage (Leboulleux et al., 2005). Besides this, several molecular genetic alterations have been studied as putative predictive markers in PTC. For example, the BRAF^V600E^ gene mutation is widely demonstrated to be associated with ETE, LNM, recurrence, and mortality (Caronia et al., 2011; Cancer Genome Atlas Research Network, 2014; Liu et al., 2014; Xing et al., 2015) and are considered to be a potential target for treatment of PTC (Cabanillas et al., 2015; Falchook et al., 2015). The underlying factors and mechanisms for the aggressiveness of PTC remain unclear.

The positive regulatory domain containing 16 (PRDM16), also known as the MDS1/EVI1-like gene 1 (MEL1), is a member of the PR domain zinc finger family. The N-terminal PR domain is characteristic of the PRDM family, which consists of 17 members known currently in the human body, named PRDM1 to PRDM17 (Fumasoni et al., 2007). The PRDM16 gene is located on human chromosome 1, encoding a protein of 1275 amino acids. It was first discovered by Mochizuki et al. (2000) in an acute myeloid leukemia (AML) and myelodysplastic syndrome study. The PRDM16 gene is a crucial regulator of the cell-fate switch between brown adipose tissue and skeletal myoblasts (Seale et al., 2007, 2008, 2011). PRDM16 overexpression is demonstrated to be a compelling poor prognostic marker of pediatric AML (Jo et al., 2015; Shiba et al., 2016). It could suppress mixed-lineage leukemia (MLL) through its intrinsic histone methyltransferase activity of the PR domain (Zhou et al., 2016). Apart from the study in hematologic neoplasm, recent research has explored controversial functions of the PRDM16 gene in solid cancers. Takahata et al. (2009) demonstrates that PRDM16 can inhibit the TGF-β signal in gastric cancer cells in cooperation with SKI. In prostate cancer, PRDM16 has an antiapoptosis function and may act in an oncogenic role (Zhu et al., 2016). Meanwhile, the high methylation status of the PRDM16 promoter is demonstrated as potential biomarker for esophageal squamous cell cancer (Peng et al., 2017) and lung cancers (Tan et al., 2014). So far, the role of the PRDM16 gene in PTC has not been established yet.

In this study, we first identified that the expression of PRDM16 was downregulated in tumors from PTC patients with significantly lower levels found in PTC patients with LNM and ETE. PRDM16 functions as a key determinant of pyruvate carboxylase (PC) overexpression and aberrant signaling in PTC cells. Furthermore, PRDM16 could regulate cell proliferation and migration via suppressing the epithelial-to-mesenchymal transition (EMT) process. Thus, our findings establish PRDM16 as a previously unsuspected key player and a novel therapeutic target for PTC.

## Materials and Methods

### Clinical Specimen and Data Collection

A total of 110 pairs of PTC tumor and adjacent normal thyroid tissues were obtained from patients who underwent thyroidectomy at the Department of Head and Neck Surgery at Fudan University Shanghai Cancer Center (FUSCC) between 2012 and 2017. The diagnosis of PTC was histopathologically confirmed, and no patient received preoperative treatment. This study was performed in accordance with the 1964 Helsinki Declaration and its later amendments or comparable ethical standards. It was approved by the human ethics committee/institutional review board of FUSCC. Written informed consent was obtained from all 110 patients. The following clinical features were collected, respectively, from patients’ records: age at diagnosis, gender, maximum tumor size, multifocality, ETE, cervical LNM, and Hashimoto’s thyroiditis. All the patients were staged using the 2016 TNM classification of the American Joint Committee on Cancer/International Union Against Cancer (Zhang et al., 2017). We also estimated the level of recurrence for each PTC patient based on 2015 American Thyroid Association (ATA) recurrence risk stratification (Haugen et al., 2016). The resected tissue samples were immediately snap-frozen in liquid nitrogen and stored at –80°C for further use.

A validation cohort from The Cancer Genome Atlas (TCGA) database was identified to confirm the preliminary findings at FUSCC. A total of 382 primary PTC patients with detailed PRDM16 expression, BRAF^V600E^ mutation, and clinical data were collected from the updated TCGA database. The TCGA cohort clinical data and the gene expression data set are available on the website of cBioPortal.

### Human Papillary Thyroid Cancer Cell Culture

Three human PTC cell lines, TPC-1, BCPAP, and K1, and one normal human thyroid epithelial cell line (Nthy-ori 3-1) were used. The Nthy-ori 3-1 cell line was purchased from Sigma. BCPAP was obtained from the cell bank at the Chinese Academy of Sciences (Shanghai, People’s Republic of China). The TPC-1 and K1 cell lines were purchased from the Cell Bank of the University of Colorado. All cell lines were cultured in RPMI-1640 medium (GIBCO) supplemented with 10% heat-inactivated fetal bovine serum (FBS; GIBCO) at 37°C in a 5% CO_2_ chamber.

### Total RNA Extraction, Reverse Transcription, and Quantitative Real-Time PCR Analysis

Total RNA was extracted from tissues and cultured cells using TRIzol Reagent (Invitrogen) according to the manufacturer’s instructions. RNA purity and concentration were determined by the NanoDrop2000 spectrophotometer. A total of 1 μg of RNA was reverse transcribed using a PrimeScript RT reagent kit (Takara, Dalian, China). For quantitative real-time PCR (qPCR), cDNA was amplified using SYBR Green Premix Ex Taq (Takara, Dalian, China) following the manufacturer’s instructions. The primers used for qPCR are listed in Supplementary Table 1. Gene expression was normalized against beta actin mRNA expression in three independent experiments. The relative mRNA expression levels were determined by the comparative Ct (2^–ΔCt^) method. The relative PRDM16 expression level for each PTC patient was determined by the ratio of the respective tumor tissue expression level to the average value of the whole 110 adjacent normal thyroid tissues expression level. The 110 enrolled PTC patients were divided into PRDM16 low- and high-expression groups based on the median value of their relative PRDM16 expression level, respectively.

### Genomic DNA Extraction and BRAF^V600E^ Mutation Analysis

Genomic DNA was extracted from the aforementioned specimens using the TIAamp Genomic DNA Kit (TIANGEN, Beijing, China) according to the manufacturer’s instructions. The DNA template was amplified for analysis of mutations in exon 15 of the BRAF gene using PCR followed by Sanger sequencing by BGI. The primers were as follows: forward: 5′-TGACTCTAAGAGGAAAGATG-3′; reverse: 5′-AATACTGGGAACTATGAAAA-3′.

### Immunohistochemistry Staining

Immunohistochemistry was carried out according to the manufacturer’s protocol. Briefly, formalin-fixed and paraffin-embedded tissue sections were deparaffinized in xylene and hydrated through descending concentrations of ethanol before being placed in a blocking solution to inhibit endogenous peroxidase activity. The slides were incubated with primary antibodies (rabbit antihuman PRDM16, 1:200 dilution, SAB, United States; rabbit antihuman pyruvate carboxylase, 1:200 dilution, Proteintech, China; rabbit antihuman Ki-67, 1:1000 dilution, Proteintech, China) at 4°C overnight. A horseradish peroxidase-conjugated rabbit secondary antibody was added for 1 h at room temperature, followed by 3,3′-diaminobenzidine (DAB) development (DAB Substrate Chromogen System, Dako Agilent Technologies, Shanghai, China) and hematoxylin and eosin (H&E) as per standard staining protocol. Slides were fixed and images obtained with the Olympus IX71 inverted microscope using the DP2-BSW Olympus image acquisition software system. The results were confirmed by two experienced pathologists who were blinded to the clinicopathologic data of the patients. The staining results were calculated on the basis of the percentage of tumor cell nuclei stained (0 = no staining, 1 = ≤ 10%, 2 = 10%–50%, 3 = > 50%) and the staining intensity (0 = negative, 1 = weak, 2 = moderate, 3 = strong). The overall score was the products of the two staining scores. An overall score of 1–5 designated low expression, and an overall score of 6–9 designated high expression of PRDM16.

### Western Blotting Assay

Protein lysates were obtained from 1 × 10^6^ cultured cells with RIPA buffer and boiled at 100°C for 10 min. Approximately 20 μg protein lysates were extracted from each sample, separated by 10% sodium dodecyl sulfate-polyacrylamide gel electrophoresis (SDS-PAGE). After being blocked in 5% nonfat milk at room temperature for 1 h, the interested protein was probed with primary antibody against human PRDM16 (1:1000 dilution, Abcam), GAPDH (1:1000, Proteintech), Vimentin (1:1000, Proteintech), E-cadherin (1:1000, Proteintech), N-cadherin (1:1000, Proteintech), MMP3 (1:1000, Proteintech), or PC (1:1000, Proteintech) at 4°C overnight and then incubated with goat antirabbit IgG or goat antimouse IgG (1:5000 dilution for both; Proteintech) at room temperature for 1 h and detected with enhanced chemiluminescence reagents (Thermo Fisher Scientific, Shanghai, China). The bands were visualized using 1-step^TM^ NBT/BCIP reagents (Thermo Fisher Scientific, Rockford, IL, United States) and detected by the Alpha Imager (Alpha Innotech, San Leandro, CA, United States). The protein expression level was quantified and normalized to GAPDH protein expression by densitometry using Image-J. Statistical data was obtained from three independent experiments.

### Plasmid Construction and Transfection

The PRDM16 sequence was synthesized and subcloned into a GV146 vector (GENECHEM Co., Ltd., Shanghai, China). PC overexpression plasmid was bought from GENECHEM Co., Ltd. (Shanghai, China), and the GV492 vector was used as a negative control. PC-siRNAs were designed and synthesized by Biotend Co., Ltd. (Shanghai, China). The sequences of PC-siRNAs are listed in Supplementary Table 2. The cells were incubated for 48 h before use in assays. Human pGL3-PC-promoter and four truncated segments of pGL3-PC-promoters for the luciferase assay were constructed by GENEWIZ Co., Ltd. (Suzhou, China). Plasmids were transfected into cells using Hieff Trans Liposomal Transfection Reagent (Yeasen, Shanghai, China) according to the manufacturer’s protocol. We adopted the qRT-PCR assay and Western blotting to evaluate overexpression of PRDM16. PC overexpression and knockdown efficiency were tested using qRT-PCR. BCPAP^PRDM16–OE^ and BCPAP^PRDM16–NC^ cell lines were filtrated from BCPAP cells transfected with PRDM16 overexpression plasmids or the negative control vector using 200 μg/ml neomycin and cultured with RPMI-1640 with 100 μg/ml neomycin. The stable cell lines were identified using fluorescence microscope for green fluorescence to ensure the transfection efficiency was more than 90%.

### Cell Viability Assay

Cell proliferation was detected using a cell-counting kit-8 (CCK-8) assay according to the manufacturer’s protocol. Briefly, the cells were seeded into 96-well plates at 3 × 10^3^ cells/well. An aliquot of 10 μl CCK-8 solution was added to each well, and the plate was incubated for 2 h at 37°C. At the indicated time points, the absorbance at 450 nm was assessed using a spectrophotometer. For each group, data from five wells were pooled. Each experiment was performed in triplicate.

### Migration Assay

The migration of PTC cells was assayed using 6.5-mm-diameter chambers with 8-μm pore filters (Transwell, 24-well cell culture; BD Biosciences). Cells were suspended at 5 × 10^4^ cells/ml in serum-free media and then 100 μl cell suspension was added to the upper chamber. Subsequently, 600 μl complete medium was added to the lower chamber. The chambers were incubated for 8 h at 37°C with 5% CO_2_. After incubation, the filters were fixed with 4% paraformaldehyde and stained with 0.5% crystal violet. The upper surface of the filters was scraped with cotton swabs to remove nonmigrating cells. The experiments were repeated in triplicate wells and the number of migrating cells in five high-power fields per filter was counted microscopically at ×200 magnification.

### Animal Studies

A 5-week-old female BALB/c nude mice were obtained from the Shanghai experimental animal center (Shanghai, China). Briefly, 1 × 10^7^ cells were subcutaneously injected into the right back skin area of BALB/c nude mice. The size of tumors was measured by Vernier caliper twice a week. After 15 days, mice were killed, and tumor tissues were collected, photographed, and examined. Paraffin-embedded tissues were sectioned for IHC analysis. Part of the tumor tissue was frozen in liquid nitrogen for following experiments. Animal experiments have been preapproved by the animal experimentation ethics committee of FUSCC, and all procedures were performed in accordance with the National Institutes of Health Guide for the Care and Use of Laboratory Animals.

### RNA-Seq Data Analysis

BCPAP and K1 cell lines were transfected with PRDM16 overexpressing plasmids. After 48 h of incubation, total RNA (5 μg per example) from the two cell lines were isolated. Paired-end sequencing was performed with Illumina Hiseq 150PE at GENEWIZ Co., Ltd. (Suzhou, China). For computational analysis of RNA-seq data, sequencing reads were aligned using the spliced read aligner HISAT2, which was supplied with the ensemble human genome assembly (Genome Reference Consortium GRCh38) as the reference genome. Gene expression levels were calculated by the fragments per kilobase of transcript per million mapped reads (FPKM).

### Luciferase Reporter Assay

Luciferase reporter assay was performed using the Dual-Luciferase Reporter Assay System (Promega, United States). The human PC promoter region was inserted into a pGL3 basic vector as pGL3-PC-Promoter. Both 100 ng of constructed pGL3-PC-promoter plasmid and 25 ng Renilla luciferase control plasmid were cotransfected into 5 × 10^3^ cells in 96-well plates. A 80 h later, luciferase activities were measured using the Dual Luciferase Assay Kit (Promega, Madison, WI, United States). Renilla luciferase was used to normalize reporter luciferase activities, which were then rescaled to vector control signals equal to unit 1.

### Chromatin Immunoprecipitation Assay

Chromatin immunoprecipitation assays were performed using the SimpleChIP Enzymatic Chromatin IP Kit (Magenetic Beads, Cell Signaling Technology, #9003). Briefly, K1 cells were cross-linked by 1% formaldehyde for 10 min at room temperature. The cross-link reaction was quenched by glycine, and cells were lysed in PBS buffer containing a protease inhibitor cocktail. Fragmented chromatin was treated with micrococcal nuclease and subjected to sonication to shear chromatin DNA into fragments with 200–500 base pairs in size. Chromatin immunoprecipitation was performed with rabbit antihistone H3 (a technical positive control; 1:50) (sc7160; Santa Cruz Biotechnology), goat anti-PRDM16 antibody (ABIN, 184809; 3 μg), and normal rabbit IgG (a negative control; 5 μg) (Cell Signaling Technology). After washing with a series of low and high salt concentration washing buffers, immunoprecipitated DNA fragments were de-crosslinked and purified. Immunoprecipitated DNA was quantified by qRT-PCR using SYBR Green Premix Ex Taq (Takara, Dalian, China) with primers for PRDM16 binding sites in PC promoter (listed in Supplementary Table 3). Fold enrichment was calculated based on the threshold cycle (CT) value of the Ig G control using the comparative CT method.

### Statistical Analysis

All statistical analyses were performed using GraphPad Prism 5.0 for windows (La Jolla, CA, United States) and SPSS ver.19.0 (SPSS Inc., Chicago, IL, United States). Categorical data was presented with frequencies and percentages. The continuous results were expressed as the mean ± the standard deviation (SD). Paired Student’s *t*-test was used to compare PRDM16 and PC mRNA expression level in tumor and adjacent normal tissues for each patient. Clinical characteristics were compared using the chi-square and Fisher’s exact tests for categorical variables and Student’s *t*-test for continuous variables. Mann–Whitney *U*-test was used for continuous data with no normal distribution. Moreover, univariate and multivariate analyses were performed to determine the risk factors for ETE in PTC in the FUSCC and TCGA cohorts using a logistic regression calculated by odds ratio (OR) and 95% confidence interval (CI). Pearson correlation analysis was used to analyze the correlation between PRDM16 and PC mRNA expression. A *P-*value less than 0.05 was considered significant.

## Results

### PRDM16 Is Downregulated in Papillary Thyroid Cancer Patients and Correlated With Poor Outcome

**FIGURE 1:**
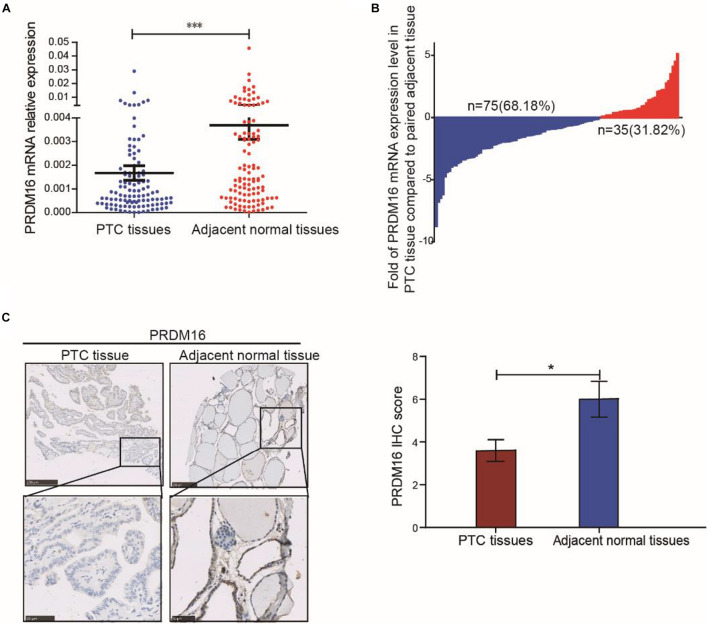
PRDM16 expression was downregulated in human PTC tissues. **(A)** PRDM16 mRNA expression level between tumor and normal tissues in 110 PTC patients from FUSCC. The results were normalized to β-actin mRNA level. **(B)** Waterfall plot shows the distribution of PRDM16 expression level in each PTC patient from FUSCC. **(C)** IHC staining of PRDM16 expression in formalin-fixed, paraffin-embedded PTC and corresponding nontumor thyroid tissues (original magnification, ×100, ×400). PRDM16 was expressed strongly negative in PTC tissues and almost positive in normal thyroid regions. ^∗∗∗^*P* < 0.001 and *P < 0.05, data were pooled from three independent experiments. FUSCC, Fudan University Shanghai Cancer Center; IHC, immunohistochemistry; PRDM16, positive regulatory domain containing 16; PTC, papillary thyroid cancer.

To evaluate PRDM16 expression in PTC tissue, qRT-PCR and IHC were performed in 110 PTC and matched adjacent thyroid tissue specimens from the FUSCC cohort. The results reveal that PRDM16 was transcriptionally downregulated in PTC samples (*P* < 0.001, Figure 1A). Seventy-five of 110 PTC patients presented a lower PRDM16 expression level in tumor tissue than that in the paired normal tissue, and only 31.82% patients had a higher PRDM16 expression level in tumor tissue compared with the paired normal tissue (Figure 1B). IHC analysis (*P* < 0.05, Figure 1C) confirmed low PRDM16 protein expression in PTC and high PRDM16 protein expression in normal thyroid tissue specimens.

Furthermore, the correlation of PRDM16 with the clinicopathological characteristics of PTC patients in the FUSCC and TCGA cohorts were analyzed as shown in Tables 1, 2. Low PRDM16 expression was significantly associated with ETE and LNM in both the FUSCC and TCGA cohorts, showing a lower expression level of PRDM16 in PTC with aggressive behaviors. Decreased PRDM16 levels correlated with N1b stage (*P* = 0.010 in the FUSCC cohort, *P* < 0.001 in the TCGA cohort). The FUSCC cohort also indicated that there was a significant difference in PRDM16 expression among gender (*P* = 0.017), multifocality (*P* = 0.002), and the maximum size of the tumor (*P* = 0.018). The low-expression group had a larger size of tumor (1.73 ± 0.81 cm) compared with the high-expression group (1.41 ± 0.69 cm). Additionally, PRDM16 low expression was significantly associated with intermediate and high levels of the 2015 ATA risk stratification system, showing a higher possibility of suffering disease recurrence. As for the TCGA cohort, PRDM16 low expression was also correlated with T stage (*P* < 0.001), TNM stage (*P* < 0.001), histological type, and BRAF^V600E^ mutation.

**TABLE 1:** Correlation of PRDM16 expression with clinicopathological characteristics in PTC of the FUSCC cohort.

**Variables**		**Low (*n* = 55)**	**High (*n* = 55)**	***P*-value**
Gender	Female	46 (56.79%)	35 (43.21%)	0.017*
	Male	9 (31.03%)	20 (68.97%)	
Age at diagnosis (years)	<55	46 (48.94%)	48 (51.06%)	0.589
	≥55	9 (56.25%)	7 (43.75%)	
ETE	Yes	20 (68.97%)	9 (31.03%)	0.017*
	No	35 (43.21%)	46 (56.79%)	
Coexistent HT	Yes	7 (58.33%)	5 (41.67%)	0.541
	No	48 (48.98%)	50 (51.02%)	
Multifocality	Unifocal	25 (71.43%)	10 (28.57%)	0.002**
	Multifocal	30 (40.00%)	45 (60.00%)	
T stage	T1-T2	38 (46.34%)	44 (53.66%)	0.189
	T3-T4	17 (60.71%)	11 (39.29%)	
LNM	N0&N1a	34 (42.50%)	46 (57.50%)	0.010*
	N1b	21 (70.00%)	9 (30.00%)	
8th AJCC TNM stage	I-II	52 (49.50%)	53 (50.50%)	0.647
	III-IV	3 (60.00%)	2 (40.00%)	
2015 ATA Risk Stratification System Level	Low	14 (35.90%)	25 (64.10%)	0.028*
	Intermediate & High	41 (57.75%)	30 (42.25%)	
BRAF^V600E^	Wild type	26 (48.15%)	28 (51.85%)	0.703
	Mutation	29 (51.79%)	27 (48.21%)	
Maximum size of tumor (Mean ± SD, cm)		1.73 ± 0.81	1.41 ± 0.69	0.018*

*Statistically significant. PRDM16, positive regulatory domain containing 16; PTC, papillary thyroid cancer; FUSCC, Fudan University Shanghai Cancer Center; HT, Hashimoto’s thyroiditis; ETE, extrathyroidal extension; LNM, lymph node metastasis; TNM, tumor–lymph node–metastasis.

**TABLE 2:** Correlation of PRDM16 expression with clinicopathological characteristics in PTC of the TCGA cohort.

**Variables**		**Low (*n* = 191)**	**High (*n* = 191)**	***P*-value**
Gender	Female	140 (49.12%)	145 (50.88%)	0.557
	Male	51 (52.58%)	46 (47.42%)	
Age at diagnosis (years)	<55	124 (48.25%)	133 (51.75%)	0.326
	≥55	67 (53.6%)	58 (46.4%)	
ETE	Yes	76 (73.08%)	28 (26.92%)	<0.001***
	No	103 (41.0%)	148 (59.0%)	
Histological type name	Classical	138 (51.30%)	131 (48.70%)	<0.001***
	Follicular	27 (32.53%)	56 (67.47%)	
	High risk subtypes (Tall cell, sclerosing, columnar)	26 (86.67%)	4 (13.33%)	
Multifocality	Unifocal	159 (50.8%)	154 (49.2%)	0.424
	Multifocal	29 (45.31%)	35 (54.69%)	
T stage	T1-T2	98 (40.8%)	142 (59.2%)	<0.001***
	T3-T4	93 (65.49%)	49 (34.51%)	
LNM	N0	73 (40.56%)	107 (59.44%)	<0.001***
	N1	104 (64.2%)	58 (35.8%)	
	NX	14 (37.8%)	23 (62.2%)	
Metastasis	M0	173 (48.3%)	185 (51.7%)	0.917
	M1	5 (50.0%)	5 (50.0%)	
8th AJCC TNM stage	I-II	111 (42.5%)	150 (57.5%)	<0.001***
	III-IV	79 (65.8%)	41 (34.2%)	
BRAF^V600E^	Wildtype	51 (32.9%)	104 (67.1%)	<0.001***
	Mutation	140 (61.67%)	87 (38.33%)	

***Statistically significant. PRDM16, positive regulatory domain containing 16; PTC, papillary thyroid cancer; TCGA, The Cancer Genomics Atlas; ETE, extrathyroidal extension; LNM, lymph node metastasis; TNM, tumor–lymph node–metastasis.

A further analysis was performed to estimate whether decreased PRDM16 expression was an independent risk factor for ETE in PTC in both the FUSCC and TCGA cohorts. Univariate analysis showed that PRDM16 low expression (OR = 2.921, 95% CI 1.186-7.192, *P* = 0.020) was a risk factor for ETE in PTC, whereas BRAF^V600E^ mutation (OR = 0.4, 95% CI 0.166-0.968, *P* = 0.042) was a protective factor. After adjusting for gender, age, LNM, multifocality, BRAF^V600E^ mutation, and PRDM16 expression level in multivariate analysis, PRDM16 low expression was found to be an independent risk factor that remained significant (OR = 2.914, 95% CI 1.074-7.911, *P* = 0.036). In the TCGA cohort, age ≥ 55 years, multifocality, LNM, BRAF^V600E^ mutation, and PRDM16 low expression were all demonstrated to be independent risk factors for ETE (Supplementary Tables 4, 5). Altogether, we supposed that PRDM16 may play a vital role on the tumorigenesis of PTC.

### Overexpression of PRDM16 Suppresses Proliferation and Migration in Papillary Thyroid Cancer Cells *in vitro*

**FIGURE 2:**
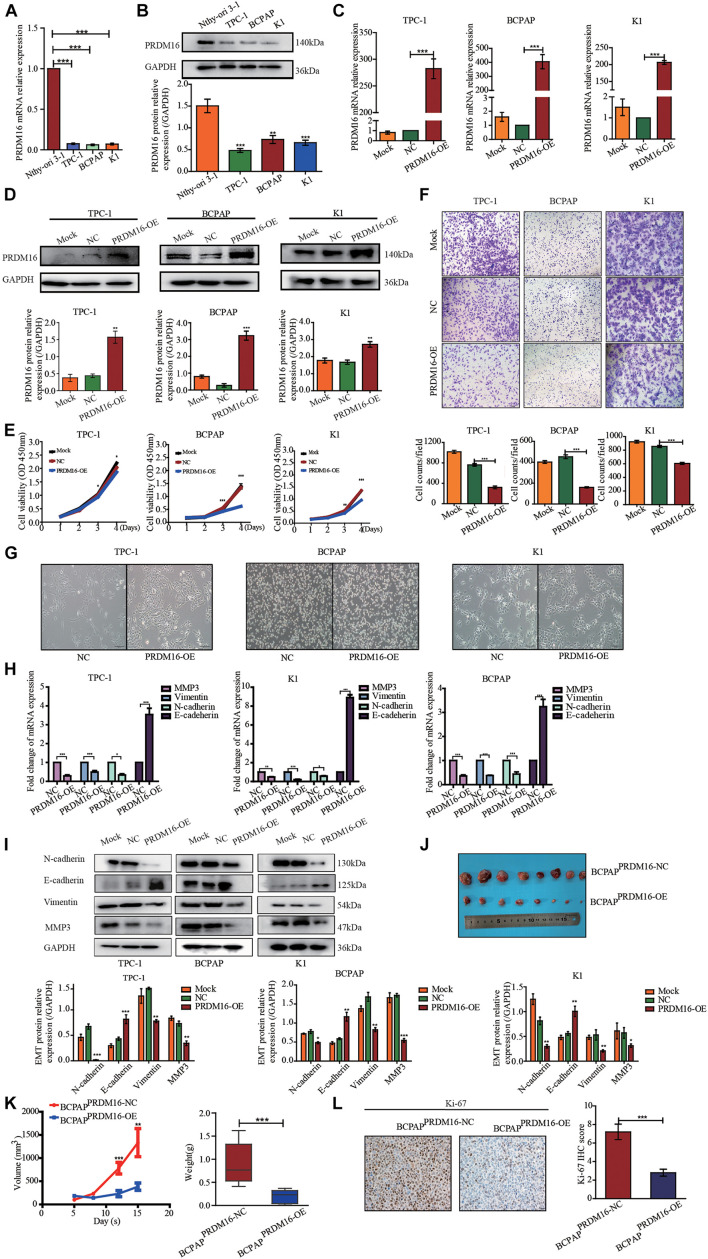
PRDM16 inhibits PTC cell proliferation, migration, and EMT *in vitro* and tumorigenicity *in vivo*. **(A)** PRDM16 mRNA expression level in one normal thyroid cell line Nthy-ori-3-1 and three PTC cell lines, TPC-1, BCPAP, and K1. The results were normalized to β-actin mRNA level. The relative quantification of PRDM16 expression in PTC cell lines was measured as fold to Nthy-ori-3-1 cell line. **(B)** Western blotting confirmed PRDM16 protein expression level in Nthy-ori-3-1, TPC-1, BCPAP, and K1 cell lines. An antibody against GAPDH was used to normalize the protein levels. Below, a plot with the quantification of the bands of Western blot experiments as the one shown. **(C,D)** Overexpression efficiency of PRDM16 in TPC-1, BCPAP, and K1 cell lines confirmed by qRT-PCR and Western blot analysis. **(E)** PRDM16 overexpression significantly inhibited cell proliferative ability of TPC-1, BCPAP, and K1 examined by CCK-8 assay. **(F)** Overexpressed PRDM16 significantly inhibited cell migration ability of TPC-1, BCPAP, and K1 examined by Transwell assay. **(G)** Mesenchymal and epithelial morphology induced by PRDM16 overexpression and negative control in TPC-1, BCPAP, and K1 cells (original magnification, ×100). **(H)** PRDM16 overexpression significantly downregulated mesenchymal markers, MMP3, N-cadherin, and Vimentin mRNA expression levels. E-cadherin, the epithelial marker, was significantly upregulated by PRDM16 overexpression in mRNA level. The results were normalized to β-actin mRNA level. **(I)** PRDM16 overexpression downregulated MMP3, N-cadherin, and Vimentin protein levels and upregulated E-cadherin protein level by Western blotting assay. The quantification results were normalized to GAPDH protein level. **(J,K)** Tumor volume and weight of recipient mice transfected with BCPAP^PRDM16–OE^ and BCPAP^PRDM16–NC^ cell lines. **(L)** IHC staining of Ki-67 expression in formalin-fixed, paraffin-embedded recipient mice PTC tissues of BCPAP^PRDM16–OE^ and BCPAP^PRDM16–NC^ (original magnification, ×100, ×400). Ki-67 was expressed significantly negative in BCPAP^PRDM16–OE^ PTC tissues. *P < 0.05, **P < 0.01, and ***P < 0.001, data were pooled from three independent experiments. IHC, immunohistochemistry; PRDM16, positive regulatory domain containing 16; PTC, papillary thyroid cancer.

To investigate whether PRDM16 expression in PTC cell lines recapitulated its expression patterns observed in PTC patient tissues, normal thyroid epithelial cell line Nthy-ori 3-1 and three PTC cell lines TPC-1, K1, and BCPAP were tested. PRDM16 was found to be downregulated in TPC-1 (*p* < 0.001), K1 (*p* < 0.001), and BCPAP (*p* < 0.001) cell lines, both transcriptionally (Figure 2A) and at protein level (Figure 2B), which was consistent with the data of human PTC tissues, suggesting PRDM16 may be a suppressor gene in PTC. Next, we performed CCK-8 and Transwell assays to illuminate the efficacy of PRDM16 on tumor cell proliferation and migration of PTC cell lines. Transcriptional (Figure 2C) and protein (Figure 2D) overexpression were successfully achieved after transfection of GV146-PRDM16 plasmids. Cell growth (Figure 2E) and migration (Figure 2F) were significantly suppressed in TPC-1, K1, and BCPAP cells overexpressing PRDM16 compared with negative controls.

### PRDM16 Inhibits Epithelial-to-Mesenchymal Transition of Papillary Thyroid Cancer Cell Lines

In the process of tumorigenesis and development, the occurrence of EMT is an important biological process of tumor cell migration and invasion of epithelial origin. Considering that PRDM16 gene was significantly associated with PTC aggressive behaviors, such as lymph node metastasis and primary extra-glandular invasion and PRDM16 overexpression could inhibit the migration ability of PTC cells, we speculated that the PRDM16 gene may be involved in the inhibition of EMT in PTC cells. We observed the cells under microscopy to determine the effects of PRDM16 on cellular morphology changes. As shown in Figure 2G, compared with the negative controls, PTC cells overexpressing PRDM16 retain their compact cell-to-cell nature and original morphology, such as short-spindle-like TPC-1 cells, round BCPAP cells, and polygonal K1 cells. On the contrary, the negative controls tended to lose their tight connection and become long-spindle shaped. Therefore, we used qRT-PCR to examine EMT-related gene marker mRNA expression in PRDM16-overexpressed TPC-1, K1, and BCPAP cells. We found that E-cadherin, the epithelial marker, was significantly increased compared with negative controls. Meanwhile, the mesenchymal markers N-cadherin, Vimentin, and MMP3 were significantly decreased compared with negative controls (Figure 2H). Next, we detected the same change trend of EMT markers in protein levels in three PTC cell lines using Western blotting (Figure 2I). Therefore, we proved that PRDM16 gene participated in the inhibition of EMT in PTC cells.

### Overexpression of PRDM16 Suppresses Papillary Thyroid Cancer Tumor Growth *in vivo*

We then tested antitumor effects of PRDM16 *in vivo*. First, we constructed a BCPAP^PRDM16–OE^ cell line stably overexpressing both PRDM16 and BCPAP^NC^ cell lines as a negative control with 100 μg/ml neomycin during cell culture. The efficiency of transfection was highly examined by fluorescence microscope (Supplementary Figure 1). To observe subcutaneous tumor formation, we injected either BCPAP^PRDM16–OE^ or BCPAP^NC^ into the flanks of nude mice. Each mouse was monitored once every 3 days, and the mice were euthanized after 2 weeks. As shown in Figures 2J,K, overexpression of PRDM16 significantly slowed the speed of tumor growth and reduced overall tumor weight *in vivo*. IHC assay with xenograft tissues showed that PRDM16 overexpression could inhibit Ki-67, a proliferation marker (Figure 2L). The data further confirmed the antitumor activities of PRDM16 in PTC.

### Pyruvate Carboxylase Might Participate in the Inhibition of Epithelial-to-Mesenchymal Transition Process by PRDM16

**FIGURE 3:**
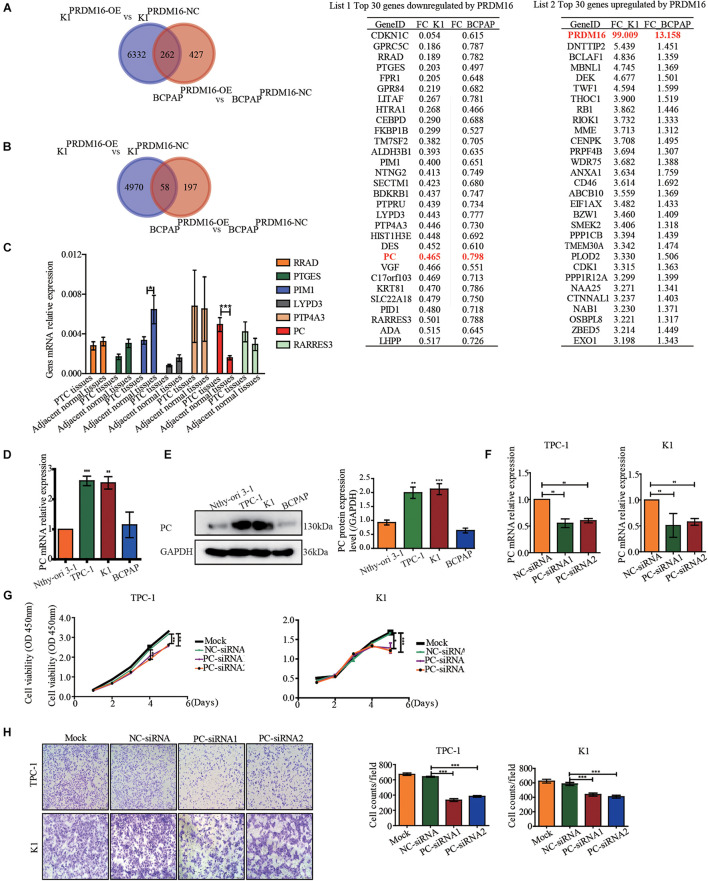
Pyruvate carboxylase was downregulated by PRDM16 and showed a reverse phenocopy of PRDM16 in PTCs. **(A)** Venn diagram shows genes downregulated by PRDM16 overexpression in both K1 and BCPAP cell lines by RNA-sequencing. The top 30 co-downregulated genes are listed in list 1. PC was significantly downregulated in both K1 and BCPAP cell lines. **(B)** Venn diagram shows genes upregulated by PRDM16 overexpression in both K1 and BCPAP cell lines by RNA-sequencing. The top 30 co-upregulated genes are listed in list 2. **(C)** QRT-PCR confirmed the targeted genes mRNA expression level in 20 pairs of human PTC and normal thyroid tissues. PC was significantly overexpressed in human PTC tissues. **(D)** PC mRNA expression level in one normal thyroid cell line Nthy-ori-3-1 and three PTC cell lines, TPC-1, BCPAP, and K1. The results were normalized to β-actin mRNA level. The relative quantification of PC expression in PTC cell lines was measured as fold to Nthy-ori-3-1 cell line. **(E)** Western blotting confirmed PC protein expression level in Nthy-ori-3-1, TPC-1, BCPAP, and K1 cell lines and further quantified against GAPDH **(F)** Knockdown efficiency of PC in TPC-1 and K1 cell lines confirmed by qRT-PCR. **(G)** PC downregulation significantly inhibited cell proliferative ability of TPC-1 and K1 examined by CCK-8 assay. **(H)** Knockdown of PC significantly inhibited cell migration ability of TPC-1 and K1 examined by Transwell assay. *P < 0.05, **P < 0.01, and ***P < 0.001, data were pooled from three independent experiments. PC, pyruvate carboxylase; PRDM16, positive regulatory domain containing 16; PTC, papillary thyroid cancer.

To further elucidate the molecular mechanism underlying the antitumor effect of PRDM16 in PTC, we performed an RNA-sequencing technique to find the downstream target genes of PRDM16 participating in the EMT process of PTC cells. First, we overexpressed PRDM16 in K1 and BCPAP cell lines and performed transcriptome sequencing. Figures 3A,B shows the top 30 genes whose expressions were co-downregulated or co-upregulated in PRDM16-overexpressing K1 and BCPAP cells. As we found before that the PRDM16 gene played a role as a cancer suppressor gene by inhibiting the EMT process in PTC, we conducted domestic and foreign literature searches for each gene on list 1 and found out seven genes are reported to participate in the EMT process. They were RRAD, PTGES, PIM1, LYPD3, PTP4A, PC, and RARRES3. Next, we tested these seven genes’ mRNA expression level in 20 pairs of FUSCC PTC tissue samples and adjacent normal thyroid tissues by qRT-PCR. As shown in Figure 3C, PC was found to be significantly upregulated in PTC tissues. Meanwhile, we also found PC was upregulated in TPC-1 and K1 cell lines compared with Nthy-ori 3-1 at both mRNA (Figure 3D) and protein (Figure 3E) levels, indicating its potential cancer-promoting gene role in PTC.

### Knockdown of Pyruvate Carboxylase Suppresses Proliferation and Migration in Papillary Thyroid Cancer Cells

We then evaluated the pro-tumorigenic role of PC in the proliferation and migration of PTC cells. We used siRNAs to knock down the PC mRNA expression level in TPC-1 and K1 and got a significant knockdown efficiency (Figure 3F). The results show that both TPC-1 (siRNA1, *P* < 0.01; siRNA2, *P* < 0.01) and K1 (siRNA1, *P* < 0.01; siRNA2, *P* < 0.01) cells transfected with PC-siRNA1 or PC-siRNA2 displayed a significantly decreased cell proliferative capacity than that of the siRNA-control vector and blank control (Figure 3G). As shown in Figure 3H, the migration capacity was examined by Transwell assay, and the migrative rate was markedly decreased in the PC-siRNA1 or PC-siRNA2-transfected TPC-1 (siRNA1, *P* < 0.001; siRNA2, *P* < 0.001) and K1 (siRNA1, *P* < 0.001; siRNA2, *P* < 0.001) than in the siRNA-control transfected cells or blank control cells. Thus, we confirmed PC’s pro-tumorigenic role in PTC cells.

### PRDM16 Exhibits an Antitumor Effect and Epithelial-to-Mesenchymal Transition Inhibition Function on Papillary Thyroid Cancer Cells via Pyruvate Carboxylase

**FIGURE 4:**
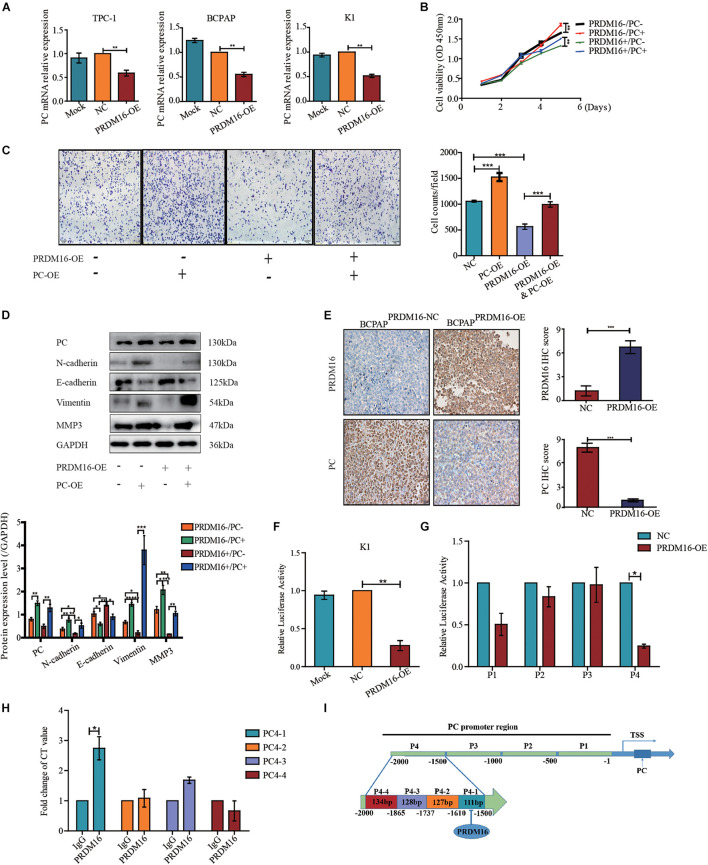
Pyruvate carboxylase recovered the effect of PRDM16 and was regulated directly by PRDM16 at transcriptional level. **(A)** PC mRNA expression was significantly downregulated by PRDM16 overexpression. The relative quantification of PC expression was measured as fold to each normal PTC cell line. The results were normalized to β-actin mRNA level. **(B)** PC overexpression significantly promoted normal BCPAP cell proliferative ability. BCPAP^PRDM16–OE^ cell line had a significant recover of proliferation after PC overexpression. The results were examined by CCK-8 assay. **(C)** PC overexpression significantly promoted normal BCPAP cell migrative ability. BCPAP^PRDM16–OE^ cell line had a significant recovery of migration by PC overexpression. The results were examined by Transwell assay. **(D)** PC overexpression promoted EMT-related protein expression in a normal BCPAP cell line and recovered the inhibition of EMT-related protein expression level in BCPAP^PRDM16–OE^ cell line. PC protein expression was reduced with no significance by quantification in BCPAP^PRDM16–OE^ cells compared with BCPAP^PRDM16–NC^ cells. **(E)** Representative IHC images showed successful upregulation of PRDM16 gene in BCPAP^PRDM16–OE^ cell line in recipient mice PTC tissues. PC was expressed significantly negative in BCPAP^PRDM16–OE^ PTC tissues compared with BCPAP^PRDM16–NC^ PTC tissues. **(F)** Luciferase reporter assay in K1 cell line with PRDM16 enhancement. The relative quantification of luciferase activity was measured as fold to negative controls. **(G)** Luciferase reporter assay showed that PRDM16 bound to specific region of PC promoter in K1 cell line. The relative quantification of Luciferase activity was measured as fold to negative controls. **(H)** QRT-PCR results of ChIP analysis showed the direct binding of PRDM16 to PC promoter region. The relative quantification of CT value was measured as fold to IgG antibody as negative control. **(I)** The map of PRDM16 binding sits in the promoter region of PC. *P < 0.05, **P < 0.01, and ***P < 0.001, data were pooled from three independent experiments. ChIP, chromatin immunoprecipitation; IHC, immunohistochemistry; PC, pyruvate carboxylase; PRDM16, positive regulatory domain containing 16; PTC, papillary thyroid cancer; TSS, transcription start site.

To confirm whether PRDM16 regulated proliferation and migration and EMT of PTC cells via PC, we determined the PC expression level by applying qRT-PCR and Western blotting in PRDM16-overexpressing TPC-1, K1, and BCPAP cells. The results show that PC mRNA level but not protein level was downregulated when PRDM16 was overexpressed in TPC-1, K1, and BCPAP cells (Figure 4A and Supplementary Figure 2). Then, we performed the rescue experiments by transfecting GV492-PC plasmids into PRDM16-overexpressing BCPAP cells and observed the impact on cell proliferation, migration, and EMT-related gene changes. In PRDM16-overexpressing BCPAP cells, PC overexpression partially reversed the effect of cell proliferation and migration caused by PRDM16 overexpression (Figures 4B,C). Meanwhile, PC overexpression could accelerate the cell proliferative and migration ability in BCPAP cells (Figures 4B,C). In addition, PC overexpression significantly reverted the change of EMT-related genes (E-cadherin, N-cadherin, Vimentin, and MMP3). We also observed that PC overexpression could decreased E-cadherin protein level and increased N-cadherin, Vimentin, and MMP3 protein levels in the BCPAP cell line (Figure 4D), indicating that PC alone could promote the EMT process in BCPAP cells. As for xenograft tissues, IHC assay results (Figure 4E) show that PC was expressed significantly lower in BCPAP^PRDM16–OE^ tumor tissues compared with BCPAP^PRDM16–NC^ tumor tissues. Taken together, these data suggest that PRDM16 could suppress tumor progression and EMT process via PC.

### PRDM16 Directly Binds to the Pyruvate Carboxylase Promoter

Next, we constructed a PC promoter luciferase reporter plasmid and performed a luciferase reporter assay to confirm the mechanistic link between PRDM16 and PC. First, we transfected PC promoter plasmids into PRDM16-overexpressing K1 cells. Compared with the control groups, PRDM16 significantly inhibited PC promoter activity in the K1 cell line (Figure 4F). To further explore the PRDM16 binding sites within the PC promoter, we constructed four 500-bp-length truncated segments of PC promoter luciferase reporter plasmids and repeated the luciferase reporter assay. PC4, the fourth truncated segment of the PC promoter, showed significantly inhibited luciferase activity in PRDM16-overexpressing K1 cells (Figure 4G). Sequences of the fourth truncated segments of PC promoter (PC4) are listed in Supplementary Table 7. To confirm the exact region of PRDM16 binding sites within PC4 truncated segment promoter, we performed ChIP in the K1 cell line and found that there was one binding region that existed at approximately from 1500 to 1610 bp upstream of the transcription start site (TSS) of PC (Figure 4H). The abridged diagram of PRDM16 directly binding to the PC promoter was illustrated in Figure 4I.

### Pyruvate Carboxylase Is Upregulated in Papillary Thyroid Cancer Tissues and Is Negatively Correlated With PRDM16 Expression

**FIGURE 5:**
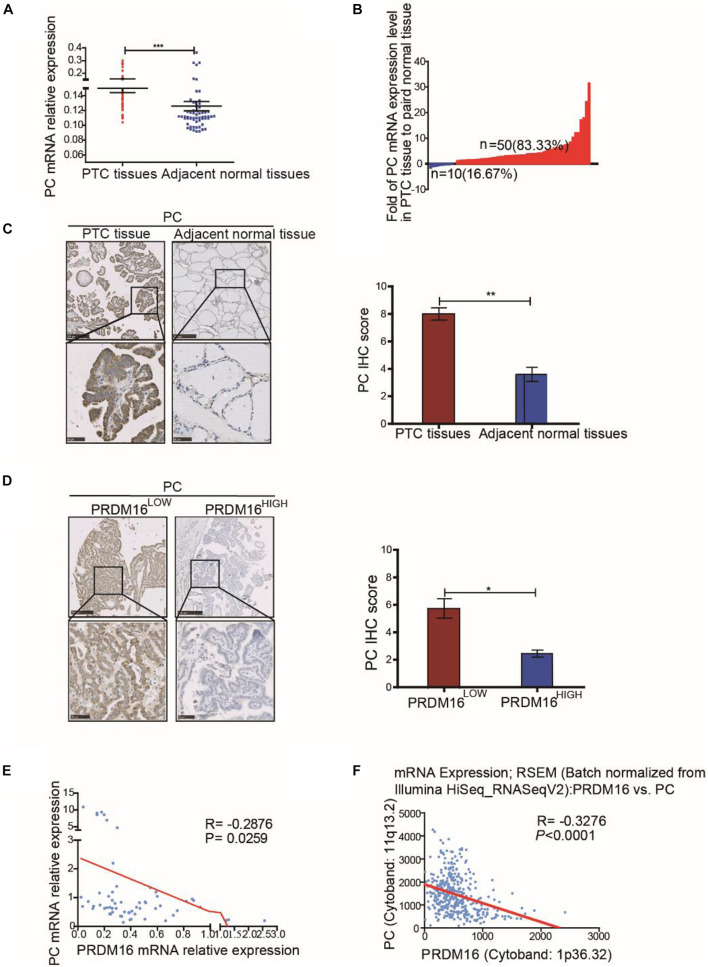
Pyruvate carboxylase was lowly expressed in human PTC tissues and was negatively correlated with PRDM16 expression. **(A)** PC mRNA expression level between tumor and normal tissues in 60 PTC patients from FUSCC. The results were normalized to β-actin mRNA level. **(B)** Waterfall plot showed the distribution of PC expression level in each PTC patients from FUSCC. **(C)** IHC staining of PC expression in formalin-fixed, paraffin-embedded PTC and corresponding nontumor thyroid tissues (original magnification, ×100, ×400). PC was strongly positive in PTC tissues and almost negative in normal thyroid regions. **(D)** Representative IHC images showed PC expression of human PTC tissues in low and high PRDM16 expression levels (original magnification, ×100, ×400). **(E)** PC and PRDM16 mRNA expression level had significantly negative correlation by qRT-PCR in 60 PTC patients from FUSCC. **(F)** PC and PRDM16 mRNA expression level had significantly negative correlation in PTC patients from TCGA. *P < 0.05, **P < 0.01, and ***P < 0.001 data were pooled from three independent experiments. FUSCC, Fudan University Shanghai Cancer Center; IHC, immunohistochemistry; PC, pyruvate carboxylase; PRDM16, positive regulatory domain containing 16; PTC, papillary thyroid cancer; TCGA, The Cancer Genome Atlas.

To determine the clinical significance of PC in PTC, we assessed PC mRNA expression in 60 pairs of PTC tissue samples and their adjacent normal thyroid tissues from FUSCC. The results revealed that PC was upregulated in PTC samples compared with adjacent normal thyroid tissues (*P* < 0.001, Figure 5A). Fifty of 60 PTC patients (83.33%) presented a higher PC expression level in tumor tissue than that in paired normal tissue although only 16.67% patients had a lower PC expression level (Figure 5B). The correlation of PC with the clinicopathological characteristics of 382 PTC patients from the TCGA cohort were analyzed as shown in Supplementary Table 6. The results of the TCGA cohort showed that high PC expression was significantly related with LNM (*P* = 0.001), ETE (*P* < 0.001), and BRAF^V600E^ mutation (*P* < 0.001).

IHC analysis (*P* < 0.01, Figure 5C) confirmed high PC protein expression in PTC and low PC protein expression in normal thyroid tissue specimens. Moreover, consistent with our previous data *in vitro*, the expression level of PC in PTC patients with low PRDM16 expression were significantly higher than that in patients with high PRDM16 expression (*P* < 0.05, Figure 5D). Furthermore, we probed into the relationship between PRDM16 mRNA expression and PC mRNA expression using linear regression in both FUSCC and TCGA cohorts. Figures 5E,F indicated that PC mRNA expression was significantly negatively related with PRDM16 mRNA expression level in PTC patients from both FUSCC (*R* = -0.2876, *P* < 0.05) and TCGA (*R* = -0.31, *P* < 0.001) cohorts. Therefore, we finally demonstrated that PC was downregulated by PRDM16 in PTC tissues.

## Discussion

Papillary thyroid cancer comprises the majority of all thyroid cancers and has a promising good prognosis. Traditional treatment for PTC includes total or subtotal thyroidectomy, radioactive iodine, and thyroid hormone inhibitory therapy. However, advanced, progressive, and radioactive iodine refractory differentiated thyroid cancers could be lethal due to limited effective treatment options. In this study, we illustrated the regulatory function of PRDM16 in the regulation of PTC. Here, we identified PRDM16 exhibited antitumor activities toward cancer cell proliferation and migration by its targeting of pyruvate carboxylase and regulating EMT. This preclinical study is the first to illustrate the involvement of PRDM16 as a potential tumor suppressor gene in PTC and explain the underlying mechanism of its regulatory role on PTC.

The presence of ETE and LNM are regarded as important factors affecting recurrence and survival of PTC. Clain et al. (2014) showed that patients with minimal ETE were significantly more likely to have extranodal extension in positive lymph nodes, manifesting its aggressive behavior. It is imperative for surgeons and patients to know the possibility of ETE before surgery in order to decide the extent of the surgery (Lee et al., 2014). Besides this, ETE is also the critical determinant for postoperative radioactive iodine treatment. Our study showed that PTC patients with ETE had a significantly higher percentage in PRDM16 low-expression level compared with that in high-expression level in both the FUSCC and TCGA cohorts. Meanwhile, PRDM16 low expression was demonstrated as an independent risk factor for ETE of PTC in the FUSCC cohort, which was also validated in TCGA cohort. Central lymph node dissection now is a widely accepted procedure and currently performed routinely in the treatment of thyroid cancer in many institutions. Although The 2015 ATA guidelines state that data to determine risk based on LNM’s location are insufficient, recent studies confirm that the persistent/recurrence disease and distant metastases were significantly more frequent in patients with lateral-cervical LN (N1b) metastases (Sapuppo et al., 2017). Our study demonstrates that a low PRDM16 level is significantly related with N1b status in both the FUSCC and TCGA cohorts. In conclusion, we identified that PTC patients with low PRDM16 expression level were more likely to have ETE and N1b presence. Therefore, PRDM16 could serve as a biomarker for aggressive behavior of PTC.

PRDM16 is a member of the PR domain zinc finger family. It was first discovered and deeply studied in AML and MLL. It could suppress MLL through its intrinsic histone methyltransferase activity of the PR domain. In Seale et al. (2008) reported that it plays a decisive role as a transcription factor in the conversion of white to brown adipocytes and is a co-activator of PPARγ15. Therefore, PRDM16 can play a role in transcription factor regulation. Apart from the study in hematologic neoplasm, recent research has explored controversial functions of PRDM16 gene in solid cancers, such as gastric, prostate, esophageal squamous cell, and lung cancers. Our results determined PRDM16 as a tumor suppressor gene in PTC and found loss of PRDM16 was prevalent in PTC tissues.

Our study discovered that PRDM16 interacted directly with the PC promoter and regulated the expression of PC transcriptionally. PC is an enzyme that exists in human mitochondria. It is a rate-limiting enzyme that catalyzes the decarboxylation of pyruvate to form acetyl-CoA. In recent years, the role of PC in tumor cells has been gradually reported (Shinde et al., 2018). Christen et al. (2016) found that the process of lung metastasis in breast cancer relies on the glucose metabolism pathway in which PC participates. The increased PC expression promotes lung metastasis in breast cancer. In addition, PC can also promote the proliferation of non-small cell lung cancer cells (Sellers et al., 2015) and malignant glioma cells (Cheng et al., 2011). As for thyroid cancer, a latest study reports that increased expression of PC in PTC could reprogram energy metabolism (Strickaert et al., 2019). Our research not only confirms the upregulated expression of PC both in PTC tissues and PTC cell lines, we also found a significant relationship between PC high expression with LNM, ETE, and BRAF^V600E^ mutation in PTC from the TCGA database, indicating its role as a tumor-promoting gene in PTC. Knockdown of PC could attenuate PTC cell proliferation and migration, which was consistent with a previous study. Our results first report that PRDM16 directly binds to the promoter region of PC and inhibited its expression, reinforcing the importance of PC in PRDM16 mediated tumorigenesis. Furthermore, increased PC expression in PTC cells reversed the inhibition of cell proliferation and migration induced by PRDM16.

The occurrence of EMT is an important biological process of tumor cell migration and invasion (Lamouille et al., 2014). By losing the characteristics of epithelial cells, tumor cells acquire the characteristics of mesenchymal cells, which can migrate and invade, making tumors more likely to invade surrounding tissues or metastasize. Therefore, exploring the molecular mechanism of the EMT process in tumor development and finding molecular targets of EMT pathways is of great significance for predicting and treating tumor invasion and metastasis. The role of EMT in the development of PTC has been proven (Knauf et al., 2011; Baquero et al., 2013). We used an RNA-sequencing technique and found seven genes that are reported to be related to the EMT process. They are RRAD (Yeom et al., 2014), PTGES (Wang et al., 2020), PIM1 (Zhao et al., 2018), LYPD3 (Oshiro et al., 2012), PTP4A (Wang et al., 2007), RARRES3 (Morales et al., 2014; Hsu et al., 2015), and PC. In Lee et al. (2012) reported a study on the relationship between PC and EMT in breast tumor cell line MCF-7. They found that PC can affect the mitochondrial respiratory process through the Wnt/Snail pathway and, thus, regulates EMT process. Unfortunately, the research team failed to prove direct binding of Wnt/Snail to the PC promoter region, but they confirmed that PC can take part in the EMT process. Our research suggests that PC could promote the EMT process in PTC. Meanwhile, our qRT-PCR results showed a significant suppression of PC mRNA expression by PRDM16, but Western blot did not show a significant downregulation of PC protein expression. Therefore, we focused on how PRDM16 downregulates PC expression at the transcriptional level. Luciferase and ChIP assays indicated that PRDM16 exhibits antitumor activities in PTC as a transcription factor through binding to PC promoter, thus inhibiting PC promoter activity at the transcriptional level and further suppressing the EMT process of PTC tumor cells. The mechanism of whether PRDM16 affects PC at the translational level remains unclear. Therefore, PRDM16 and PC, as key molecules involved in the EMT pathway of PTC cells, may be involved in the migration behavior of PTC, including extra-glandular invasion of tumors, LNM, and even distant metastasis. PRDM16 and PC can be used as molecular targets for future EMT therapy on PTC.

The main limitations of this study are the small sample size of the FUSCC cohort and the retrospective nature of the data analysis. First, the small sample size and the selection bias in the FUSCC cohort led to inconsistent results compared with that in the TCGA cohort. We demonstrate that a low PRDM16 expression level was significantly associated with histological type and BRAF^V600E^ mutation in the TCGA cohort. As for the histological type, the tall-cell and sclerosing types of PTC, which are aggressive histologies, have a higher proportion in the PRDM16 low level group. However, the FUSCC cohort had no patients with a tall-cell or sclerosing histological type of PTC; therefore, it was impossible to assess its relationship with PRDM16. In the FUSCC cohort, we found out that the percentage of patients harboring a BRAF^V600E^ mutation in the PRDM16 low-expression group (51.79%) was higher than that in high-expression group (48.21%), but it failed to show significance. Meanwhile, small sample size and maldistribution of clinical data led to an opposite result for BRAF^V600E^ in an ETE risk factor multivariable analysis between our FUSCC cohort and the TCGA cohort. Therefore, more patients should be enrolled in the study to enlarge the sample size to further explore the significant correlated clinical-pathological factors with PRDM16 and the predictive factors for extrathyroidal extension. Second, all of the studied patients should have long-term follow-up to see if PRDM16 could affect the prognosis of PTC. Moreover, further studies are needed to explore the underlying molecular mechanisms of PRDM16 function in PTC.

In summary, our study demonstrates that PRDM16 is downregulated in PTC tissues and cell lines TPC-1, K1, and BCPAP. Meanwhile, the relationship between PRDM16 low expression and LNM and ETE of PTC patients from TCGA was in line with our FUSCC data, indicating that PRDM16 can be a novel marker for the prognosis of PTC patients. *In vitro* and *in vivo* data show that overexpression of PRDM16 attenuated cell proliferation and migration of PTC cells, confirming the vital function of PRDM16 in PTC. Further mechanism study demonstrates that the role of PRDM16 in PTC may result from the regulation of EMT via its directly binding to the PC promoter. Thus, PRDM16 may present as a new biomarker of favorable prognosis in PTC and as a potential novel target for the treatment of PTC.

## Data Availability Statement

The datasets presented in this study can be found in online repositories. The names of the repository/repositories and accession number(s) can be found below: https://www.ncbi.nlm.nih.gov/Traces/study/?acc=PRJNA739686&o=acc_s%3Aa, PRJNA739686.

## Ethics Statement

The studies involving human participants were reviewed and approved by the Human Ethics Committee/Institutional Review Board of Fudan University Shanghai Cancer Center. The patients/participants provided their written informed consent to participate in this study. The animal study was reviewed and approved by the Animal Welfare and Ethics Group, Laboratory Animal Science Department, Fudan University.

## Author Contributions

W-LL performed the cell line culture, Luciferase assay, ChIP assay, statistical analysis, and wrote the manuscript. QG performed the FUSCC human tissues collection and helped to write the manuscript. DW performed the cell proliferation and migration, and Western blot. BM helped the mechanism exploration and statistical analysis. W-BX finished the xenograft assays. J-QH did the qRT-PCR. W-JW finished the IHC assay. D-SL and YW provided the human PTC tissues. JX and TL provided the technical support and tissue clinical data. Q-HJ designed the project and supervised the project. All authors read and approved the final manuscript.

## Conflict of Interest

The authors declare that the research was conducted in the absence of any commercial or financial relationships that could be construed as a potential conflict of interest.

## Publisher’s Note

All claims expressed in this article are solely those of the authors and do not necessarily represent those of their affiliated organizations, or those of the publisher, the editors and the reviewers. Any product that may be evaluated in this article, or claim that may be made by its manufacturer, is not guaranteed or endorsed by the publisher.

## References

[B1] Baquero P. Sanchez-Hernandez I. Jimenez-Mora E. Orgaz J. L. Jimenez B. Chiloeches A. (2013). (V600E)BRAF promotes invasiveness of thyroid cancer cells by decreasing E-cadherin expression through a Snail-dependent mechanism. *Cancer Lett.* 335 232–241. 10.1016/j.canlet.2013.02.033 23435375

[B2] Bray F. Ferlay J. Soerjomataram I. Siegel R. L. Torre L. A. Jemal A. (2018). Global cancer statistics 2018: GLOBOCAN estimates of incidence and mortality worldwide for 36 cancers in 185 countries. *CA Cancer J. Clin.* 68 394–424. 10.3322/caac.21492 30207593

[B3] Cabanillas M. E. Patel A. Danysh B. P. Dadu R. Kopetz S. Falchook G. (2015). BRAF inhibitors: experience in thyroid cancer and general review of toxicity. *Horm. Cancer* 6 21–36. 10.1007/s12672-014-0207-9 25467940 PMC4312215

[B4] Cancer Genome Atlas Research Network (2014). Integrated genomic characterization of papillary thyroid carcinoma. *Cell* 159 676–690. 10.1016/j.cell.2014.09.050 25417114 PMC4243044

[B5] Carling T. Udelsman R. (2014). Thyroid cancer. *Annu. Rev. Med.* 65 125–137. 10.1146/annurev-med-061512-105739 24274180

[B6] Caronia L. M. Phay J. E. Shah M. H. (2011). Role of BRAF in thyroid oncogenesis. *Clin. Cancer Res.* 17 7511–7517. 10.1158/1078-0432.CCR-11-1155 21900390

[B7] Chen A. Y. Jemal A. Ward E. M. (2009). Increasing incidence of differentiated thyroid cancer in the United States, 1988-2005. *Cancer* 115 3801–3807. 10.1002/cncr.24416 19598221

[B8] Cheng T. Sudderth J. Yang C. Mullen A. R. Jin E. S. Mates J. M. (2011). Pyruvate carboxylase is required for glutamine-independent growth of tumor cells. *Proc. Natl. Acad. Sci. U.S.A.* 108 8674–8679. 10.1073/pnas.1016627108 21555572 PMC3102381

[B9] Christen S. Lorendeau D. Schmieder R. Broekaert D. Metzger K. Veys K. (2016). Breast cancer-derived lung metastases show increased pyruvate carboxylase-dependent anaplerosis. *Cell Rep.* 17 837–848. 10.1016/j.celrep.2016.09.042 27732858

[B10] Clain J. B. Scherl S. Dos Reis L. Turk A. Wenig B. M. Mehra S. (2014). Extrathyroidal extension predicts extranodal extension in patients with positive lymph nodes: an important association that may affect clinical management. *Thyroid* 24 951–957. 10.1089/thy.2013.0557 24443878

[B11] Falchook G. S. Millward M. Hong D. Naing A. Piha-Paul S. Waguespack S. G. (2015). BRAF inhibitor dabrafenib in patients with metastatic BRAF-mutant thyroid cancer. *Thyroid* 25 71–77. 10.1089/thy.2014.0123 25285888 PMC4291160

[B12] Fumasoni I. Meani N. Rambaldi D. Scafetta G. Alcalay M. Ciccarelli F. D. (2007). Family expansion and gene rearrangements contributed to the functional specialization of PRDM genes in vertebrates. *BMC Evol. Biol.* 7:187. 10.1186/1471-2148-7-187 17916234 PMC2082429

[B13] Haugen B. R. Alexander E. K. Bible K. C. Doherty G. M. Mandel S. J. Nikiforov Y. E. (2016). 2015 American thyroid association management guidelines for adult patients with thyroid nodules and differentiated thyroid cancer: the American thyroid association guidelines task force on thyroid nodules and differentiated thyroid cancer. *Thyroid* 26 1–133. 10.1089/thy.2015.0020 26462967 PMC4739132

[B14] Hay I. D. Thompson G. B. Grant C. S. Bergstralh E. J. Dvorak C. E. Gorman C. A. (2002). Papillary thyroid carcinoma managed at the Mayo Clinic during six decades (1940-1999): temporal trends in initial therapy and long-term outcome in 2444 consecutively treated patients. *World J. Surg.* 26 879–885. 12016468 10.1007/s00268-002-6612-1

[B15] Hsu T. H. Jiang S. Y. Chang W. L. Eckert R. L. Scharadin T. M. Chang T. C. (2015). Involvement of RARRES3 in the regulation of Wnt proteins acylation and signaling activities in human breast cancer cells. *Cell Death Differ.* 22:1561. 10.1038/cdd.2015.90 26256516 PMC4532785

[B16] Jo A. Mitani S. Shiba N. Hayashi Y. Hara Y. Takahashi H. (2015). High expression of EVI1 and MEL1 is a compelling poor prognostic marker of pediatric AML. *Leukemia* 29 1076–1083. 10.1038/leu.2015.5 25567132

[B17] Kitahara C. M. Sosa J. A. (2016). The changing incidence of thyroid cancer. *Nat. Rev. Endocrinol.* 12 646–653. 10.1038/nrendo.2016.110 27418023 PMC10311569

[B18] Knauf J. A. Sartor M. A. Medvedovic M. Lundsmith E. Ryder M. Salzano M. (2011). Progression of BRAF-induced thyroid cancer is associated with epithelial-mesenchymal transition requiring concomitant MAP kinase and TGFbeta signaling. *Oncogene* 30 3153–3162. 10.1038/onc.2011.44 21383698 PMC3136543

[B19] Lamouille S. Xu J. Derynck R. (2014). Molecular mechanisms of epithelial-mesenchymal transition. *Nat. Rev. Mol. Cell Biol.* 15 178–196. 10.1038/nrm3758 24556840 PMC4240281

[B20] Leboulleux S. Rubino C. Baudin E. Caillou B. Hartl D. M. Bidart J. M. (2005). Prognostic factors for persistent or recurrent disease of papillary thyroid carcinoma with neck lymph node metastases and/or tumor extension beyond the thyroid capsule at initial diagnosis. *J. Clin. Endocrinol. Metab.* 90 5723–5729. 10.1210/jc.2005-0285 16030160

[B21] Lee D. Y. Kwon T. K. Sung M. W. Kim K. H. Hah J. H. (2014). Prediction of extrathyroidal extension using ultrasonography and computed tomography. *Int. J. Endocrinol.* 2014:351058. 10.1155/2014/351058 25525431 PMC4265702

[B22] Lee S. Y. Jeon H. M. Ju M. K. Kim C. H. Yoon G. Han S. I. (2012). Wnt/Snail signaling regulates cytochrome C oxidase and glucose metabolism. *Cancer Res.* 72 3607–3617. 10.1158/0008-5472.CAN-12-0006 22637725

[B23] Liu X. Qu S. Liu R. Sheng C. Shi X. Zhu G. (2014). TERT promoter mutations and their association with BRAF V600E mutation and aggressive clinicopathological characteristics of thyroid cancer. *J. Clin. Endocrinol. Metab.* 99 E1130–E1136. 10.1210/jc.2013-4048 24617711 PMC4037723

[B24] Mazzaferri E. L. Massoll N. (2002). Management of papillary and follicular (differentiated) thyroid cancer: new paradigms using recombinant human thyrotropin. *Endocr. Relat. Cancer* 9 227–247. 12542401 10.1677/erc.0.0090227

[B25] Mochizuki N. Shimizu S. Nagasawa T. Tanaka H. Taniwaki M. Yokota J. (2000). A novel gene, MEL1, mapped to 1p36.3 is highly homologous to the MDS1/EVI1 gene and is transcriptionally activated in t(1;3)(p36;q21)-positive leukemia cells. *Blood* 96 3209–3214. 11050005

[B26] Morales M. Arenas E. J. Urosevic J. Guiu M. Fernandez E. Planet E. (2014). RARRES3 suppresses breast cancer lung metastasis by regulating adhesion and differentiation. *EMBO Mol. Med.* 6 865–881. 10.15252/emmm.201303675 24867881 PMC4119352

[B27] Oshiro R. Yamamoto H. Takahashi H. Ohtsuka M. Wu X. Nishimura J. (2012). C4.4A is associated with tumor budding and epithelial-mesenchymal transition of colorectal cancer. *Cancer Sci.* 103 1155–1164. 10.1111/j.1349-7006.2012.02263.x 22404718 PMC7685091

[B28] Peng X. Xue H. Lu L. Shi P. Wang J. Wang J. (2017). Accumulated promoter methylation as a potential biomarker for esophageal cancer. *Oncotarget* 8 679–691. 10.18632/oncotarget.13510 27893424 PMC5352188

[B29] Sapuppo G. Palermo F. Russo M. Tavarelli M. Masucci R. Squatrito S. (2017). Latero-cervical lymph node metastases (N1b) represent an additional risk factor for papillary thyroid cancer outcome. *J. Endocrinol. Invest.* 40 1355–1363. 10.1007/s40618-017-0714-y 28646475

[B30] Seale P. Bjork B. Yang W. Kajimura S. Chin S. Kuang S. (2008). PRDM16 controls a brown fat/skeletal muscle switch. *Nature* 454 961–967. 10.1038/nature07182 18719582 PMC2583329

[B31] Seale P. Conroe H. M. Estall J. Kajimura S. Frontini A. Ishibashi J. (2011). Prdm16 determines the thermogenic program of subcutaneous white adipose tissue in mice. *J. Clin. Invest.* 121 96–105. 10.1172/JCI44271 21123942 PMC3007155

[B32] Seale P. Kajimura S. Yang W. Chin S. Rohas L. M. Uldry M. (2007). Transcriptional control of brown fat determination by PRDM16. *Cell Metab.* 6 38–54. 10.1016/j.cmet.2007.06.001 17618855 PMC2564846

[B33] Sellers K. Fox M. P. Bousamra M. II Slone S. P. Higashi R. M. Miller D. M. (2015). Pyruvate carboxylase is critical for non-small-cell lung cancer proliferation. *J. Clin. Invest.* 125 687–698. 10.1172/JCI72873 25607840 PMC4319441

[B34] Shiba N. Ohki K. Kobayashi T. Hara Y. Yamato G. Tanoshima R. (2016). High PRDM16 expression identifies a prognostic subgroup of pediatric acute myeloid leukaemia correlated to FLT3-ITD, KMT2A-PTD, and NUP98-NSD1: the results of the Japanese Paediatric Leukaemia/Lymphoma Study Group AML-05 trial. *Br. J. Haematol.* 172 581–591. 10.1111/bjh.13869 26684393

[B35] Shinde A. Wilmanski T. Chen H. Teegarden D. Wendt M. K. (2018). Pyruvate carboxylase supports the pulmonary tropism of metastatic breast cancer. *Breast Cancer Res.* 20:76. 10.1186/s13058-018-1008-9 30005601 PMC6045837

[B36] Strickaert A. Corbet C. Spinette S. A. Craciun L. Dom G. Andry G. (2019). Reprogramming of energy metabolism: increased expression and roles of pyruvate carboxylase in papillary thyroid cancer. *Thyroid* 29 845–857. 10.1089/thy.2018.0435 30990120

[B37] Takahata M. Inoue Y. Tsuda H. Imoto I. Koinuma D. Hayashi M. (2009). SKI and MEL1 cooperate to inhibit transforming growth factor-β signal in gastric cancer cells. *J. Biol. Chem.* 284 3334–3344. 10.1074/jbc.M808989200 19049980

[B38] Tan S. Hu R. Liu J. Tan Y. Liu W. (2014). Methylation of PRDM2, PRDM5 and PRDM16 genes in lung cancer cells. *Int. J. Clin. Exp. Pathol.* 7 2305–2311. 24966940 PMC4069964

[B39] Wang H. Quah S. Y. Dong J. M. Manser E. Tang J. P. Zeng Q. (2007). PRL-3 down-regulates PTEN expression and signals through PI3K to promote epithelial-mesenchymal transition. *Cancer Res.* 67 2922–2926. 10.1158/0008-5472.CAN-06-3598 17409395

[B40] Wang T. Jing B. Xu D. Liao Y. Song H. Sun B. (2020). PTGES/PGE2 signaling links immunosuppression and lung metastasis in Gprc5a-knockout mouse model. *Oncogene* 39 3179–3194. 10.1038/s41388-020-1207-6 32060421 PMC7142021

[B41] Xing M. Alzahrani A. S. Carson K. A. Shong Y. K. Kim T. Y. Viola D. (2015). Association between BRAF V600E mutation and recurrence of papillary thyroid cancer. *J. Clin. Oncol.* 33 42–50. 10.1200/JCO.2014.56.8253 25332244 PMC4268252

[B42] Yeom S. Y. Nam D. H. Park C. (2014). RRAD promotes EGFR-mediated STAT3 activation and induces temozolomide resistance of malignant glioblastoma. *Mol. Cancer Ther.* 13 3049–3061. 10.1158/1535-7163.MCT-14-0244 25313011

[B43] Zhang M. Wu J. Mao K. Deng H. Yang Y. Zhou E. (2017). Role of transforming growth factor-beta1 in triple negative breast cancer patients. *Int. J. Surg.* 45 72–76. 10.1016/j.ijsu.2017.07.080 28754615

[B44] Zhao B. Liu L. Mao J. Zhang Z. Wang Q. Li Q. (2018). PIM1 mediates epithelial-mesenchymal transition by targeting Smads and c-Myc in the nucleus and potentiates clear-cell renal-cell carcinoma oncogenesis. *Cell Death Dis.* 9:307. 10.1038/s41419-018-0348-9 29472550 PMC5833424

[B45] Zhou B. Wang J. Lee S. Y. Xiong J. Bhanu N. Guo Q. (2016). PRDM16 suppresses MLL1r leukemia via intrinsic histone methyltransferase activity. *Mol. Cell* 62 222–236. 10.1016/j.molcel.2016.03.010 27151440 PMC5061593

[B46] Zhu S. Xu Y. Song M. Chen G. Wang H. Zhao Y. (2016). PRDM16 is associated with evasion of apoptosis by prostatic cancer cells according to RNA interference screening. *Mol. Med. Rep.* 14 3357–3361. 10.3892/mmr.2016.5605 27511603

