# Supplementary material for: PRDM16 Inhibits Cell Proliferation and Migration via Epithelial-to-Mesenchymal Transition by Directly Targeting Pyruvate Carboxylase in Papillary Thyroid Cancer
Source: Front Cell Dev Biol. 2021 Nov 2;9:723777. doi: 10.3389/fcell.2021.723777 (PMC8593917; doi:10.3389/fcell.2021.723777)
Supplement: Supplementary file 1 [file Data_Sheet_1.ZIP › Full scans of the original gels.pptx]

## Slide 1
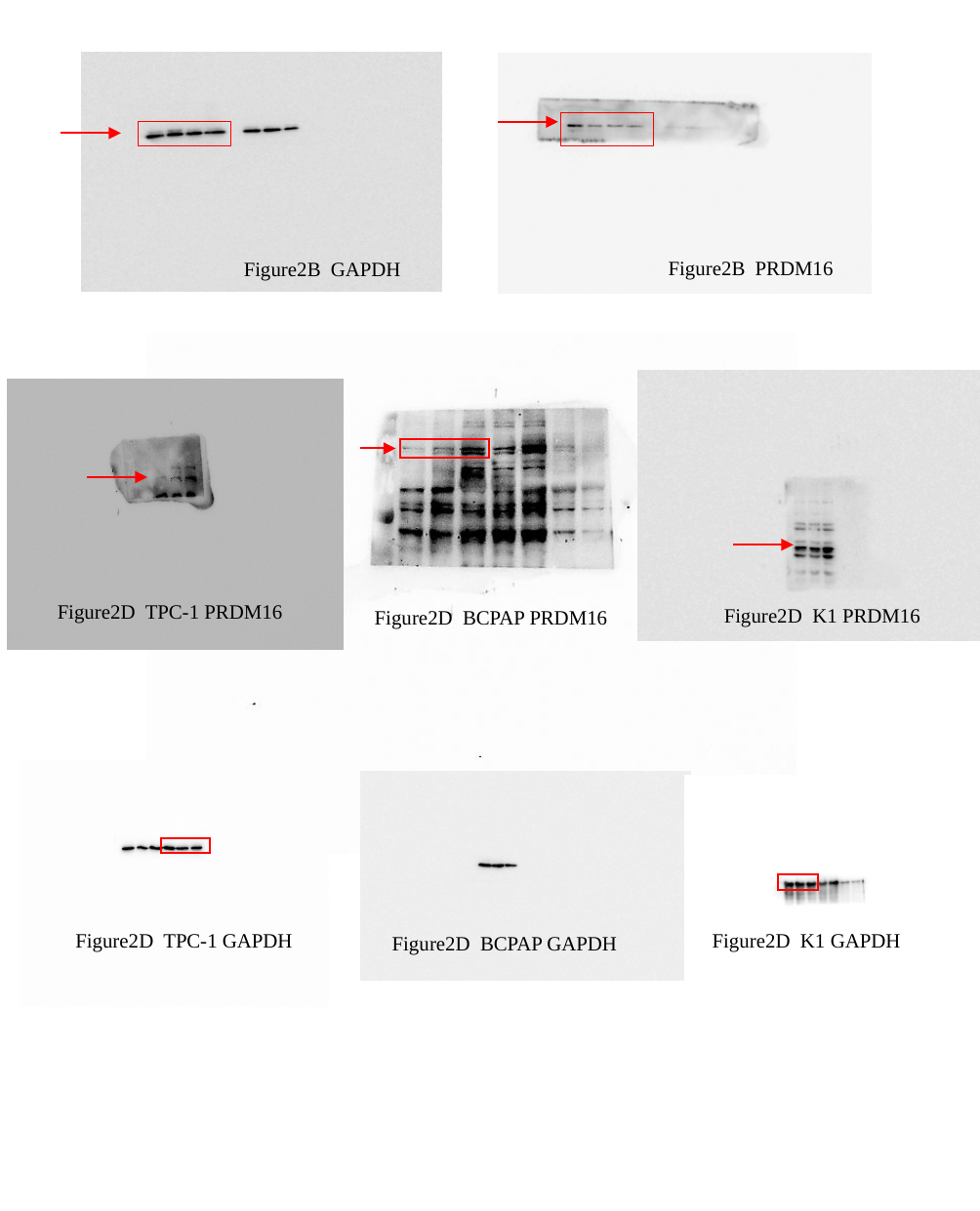

Figure2B PRDM16
Figure2B GAPDH
Figure2D BCPAP PRDM16
Figure2D K1 PRDM16
Figure2D TPC-1 PRDM16
Figure2D TPC-1 GAPDH
Figure2D BCPAP GAPDH
Figure2D K1 GAPDH
Figure2D TPC-1 GAPDH

## Slide 2
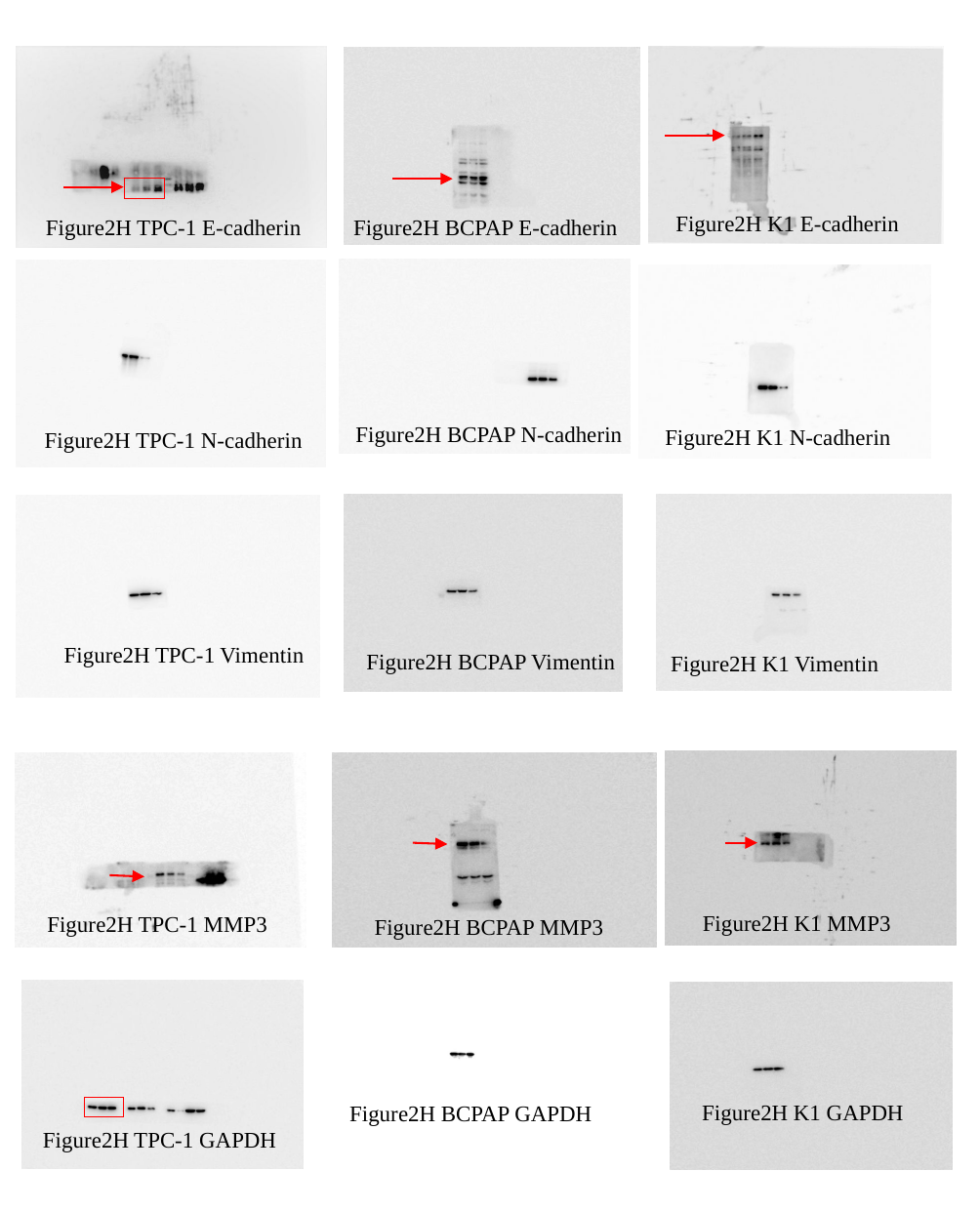

Figure2H TPC-1 E-cadherin
Figure2H K1 E-cadherin
Figure2H BCPAP E-cadherin
Figure2H K1 N-cadherin
Figure2H BCPAP N-cadherin
Figure2H TPC-1 N-cadherin
Figure2H BCPAP Vimentin
Figure2H TPC-1 Vimentin
Figure2H K1 Vimentin
Figure2H K1 MMP3
Figure2H TPC-1 MMP3
Figure2H BCPAP MMP3
Figure2H BCPAP GAPDH
Figure2H K1 GAPDH
Figure2H TPC-1 GAPDH

## Slide 3
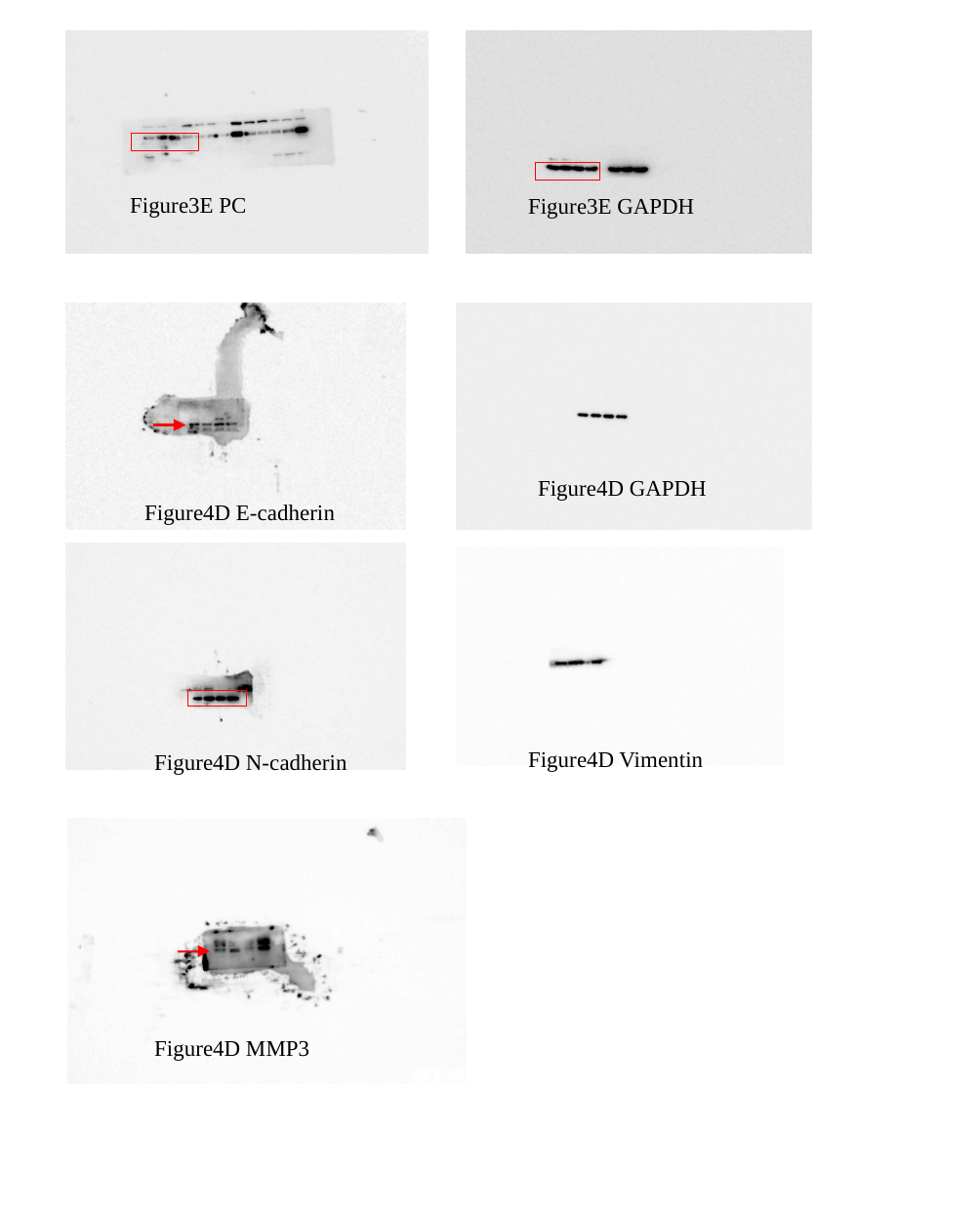

Figure3E PC
Figure3E GAPDH
Figure4D GAPDH
Figure4D E-cadherin
Figure4D Vimentin
Figure4D N-cadherin
Figure4D MMP3

## Slide 4
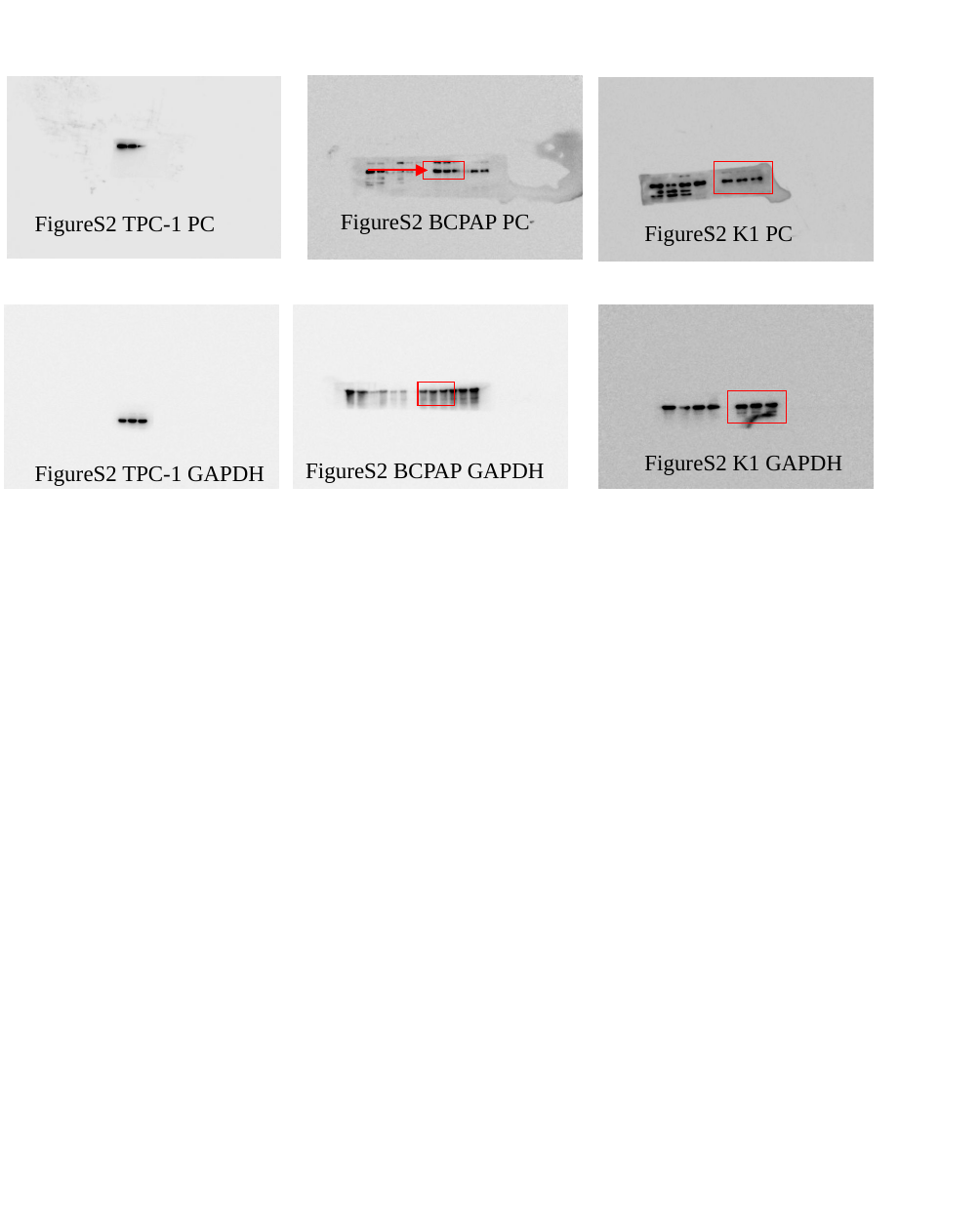

FigureS2 BCPAP PC
FigureS2 TPC-1 PC
FigureS2 BCPAP PC
FigureS2 K1 PC
FigureS2 K1 GAPDH
FigureS2 BCPAP GAPDH
FigureS2 TPC-1 GAPDH
