# Supplementary figures and images for: PRDM16 Inhibits Cell Proliferation and Migration via Epithelial-to-Mesenchymal Transition by Directly Targeting Pyruvate Carboxylase in Papillary Thyroid Cancer
Source: Front Cell Dev Biol. 2021 Nov 2;9:723777. doi: 10.3389/fcell.2021.723777 (PMC8593917; doi:10.3389/fcell.2021.723777)

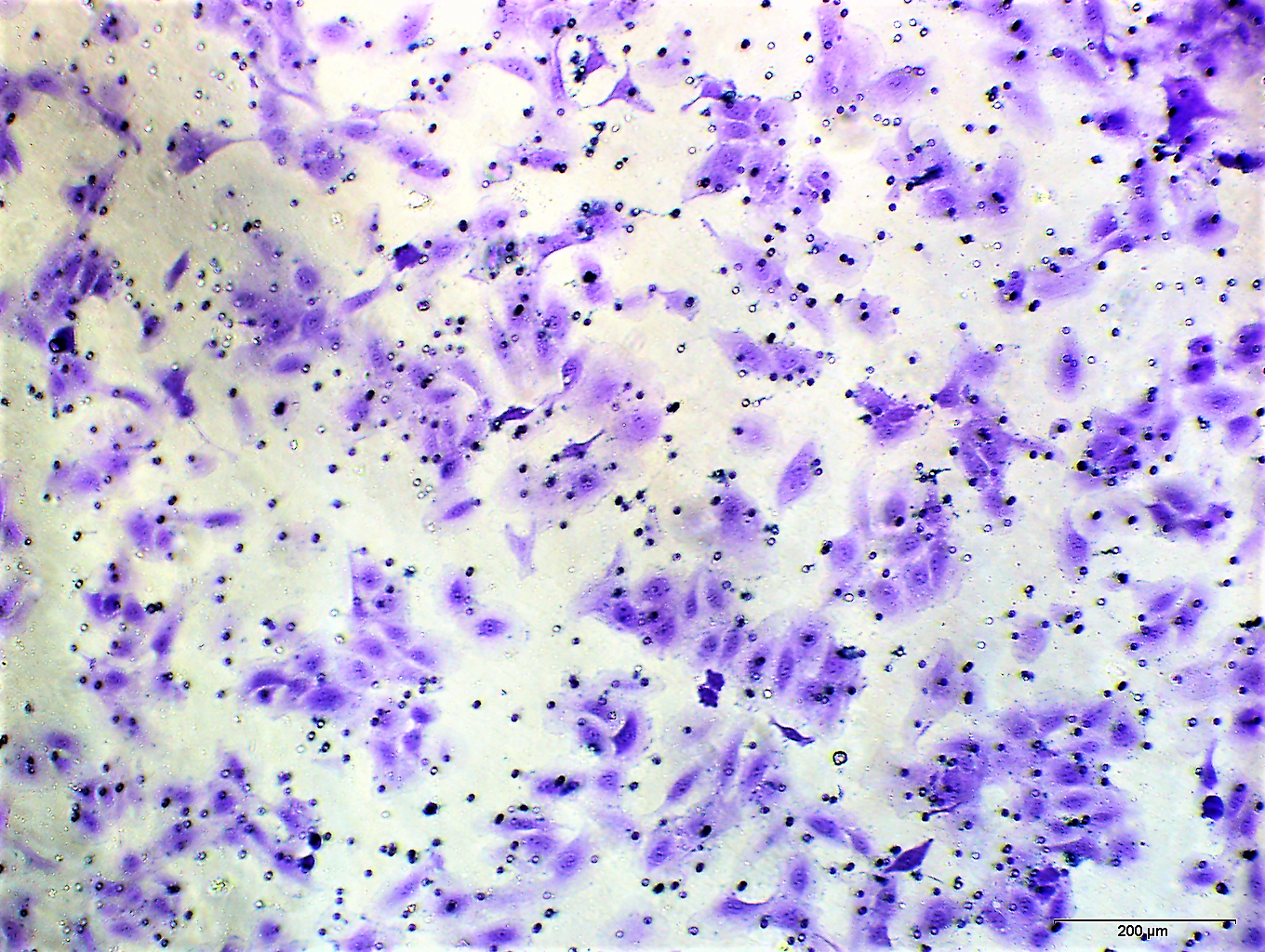

Supplement: Supplementary file 3 [file Data_Sheet_3.ZIP › Transwell figures-2/Transwell K1-PC-SI1.tif]

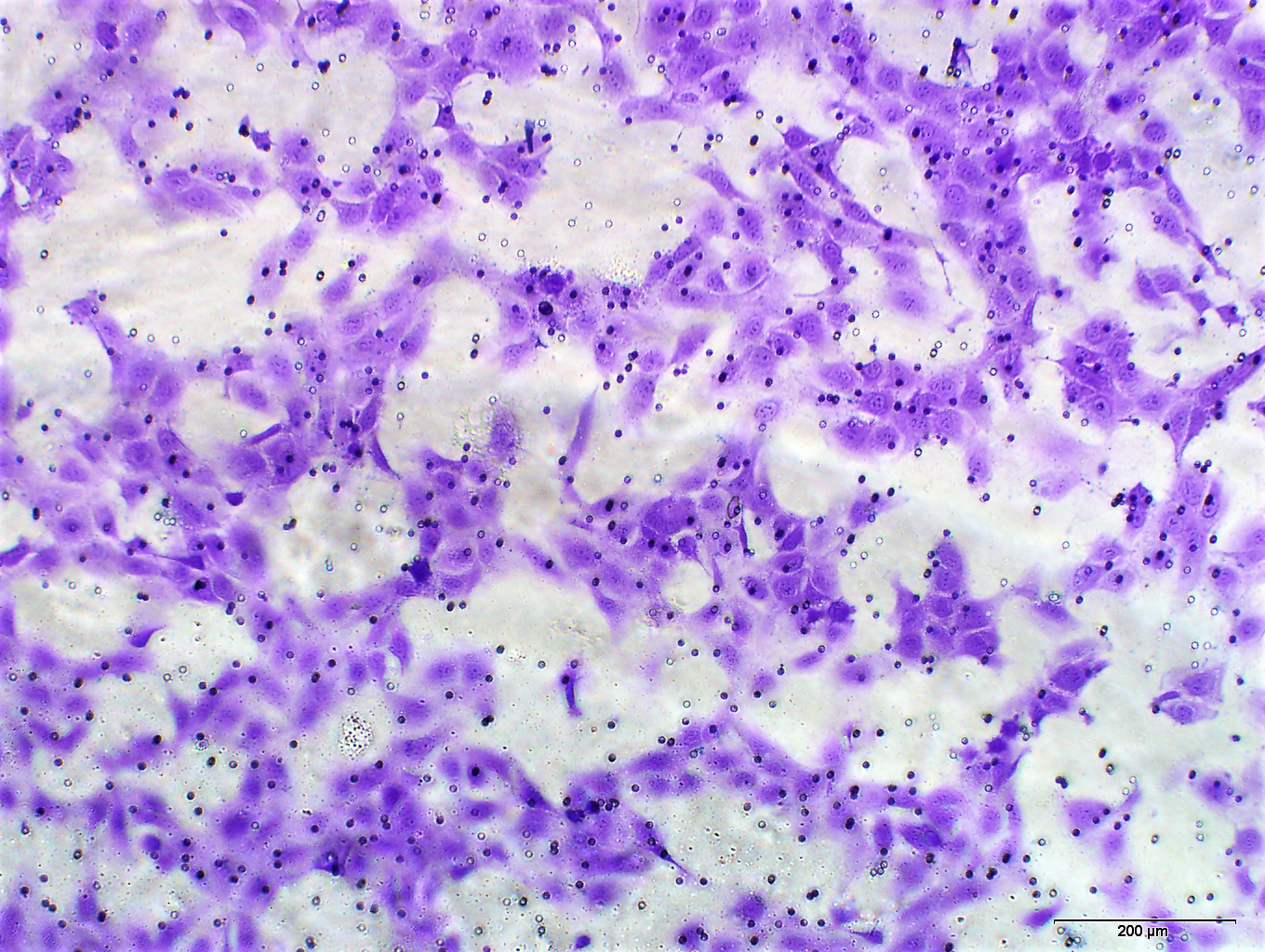

Supplement: Supplementary file 3 [file Data_Sheet_3.ZIP › Transwell figures-2/Transwell K1-PC-SI2.tif]

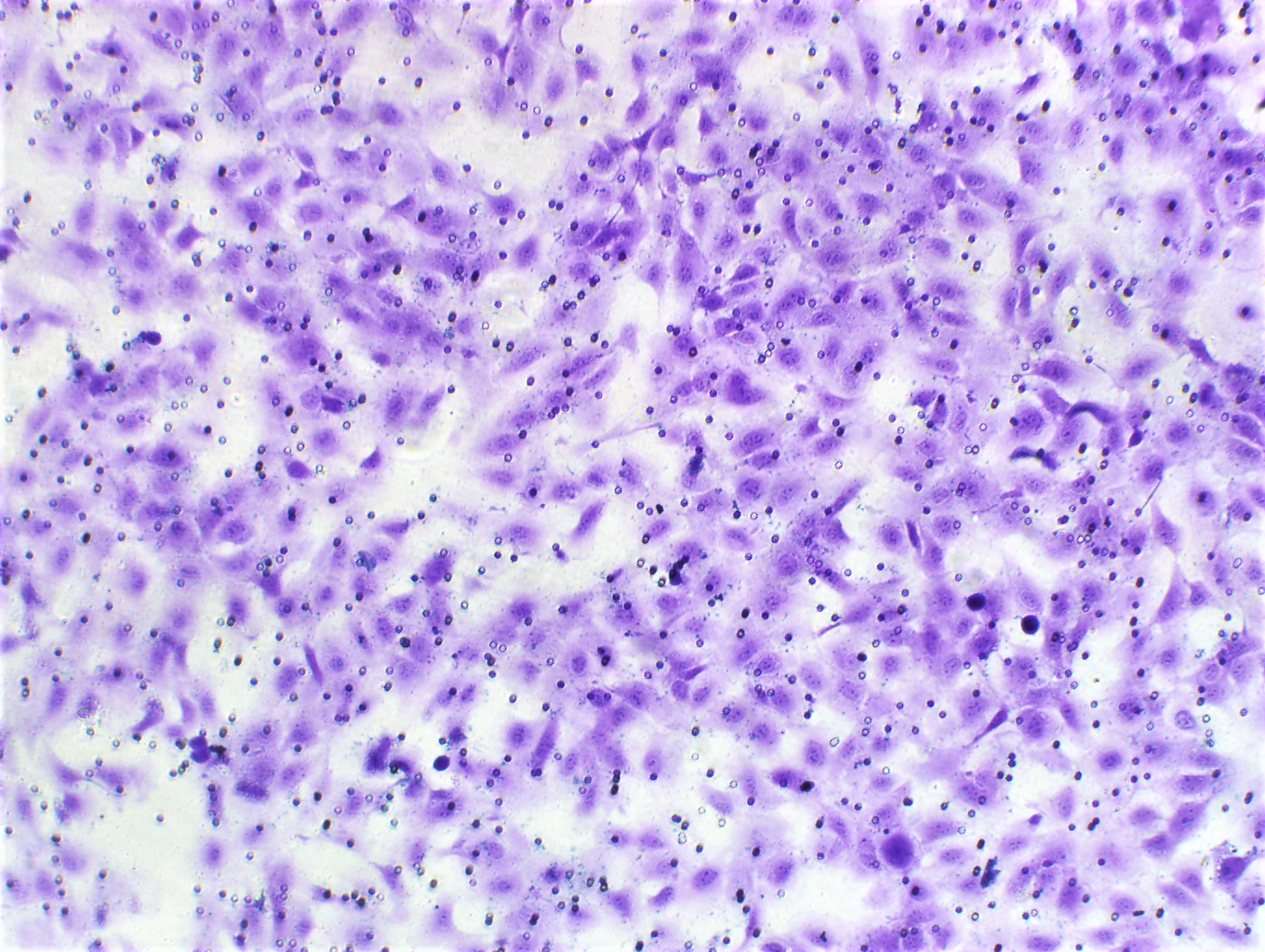

Supplement: Supplementary file 3 [file Data_Sheet_3.ZIP › Transwell figures-2/Transwell K1-PCsi-Mock.tif]

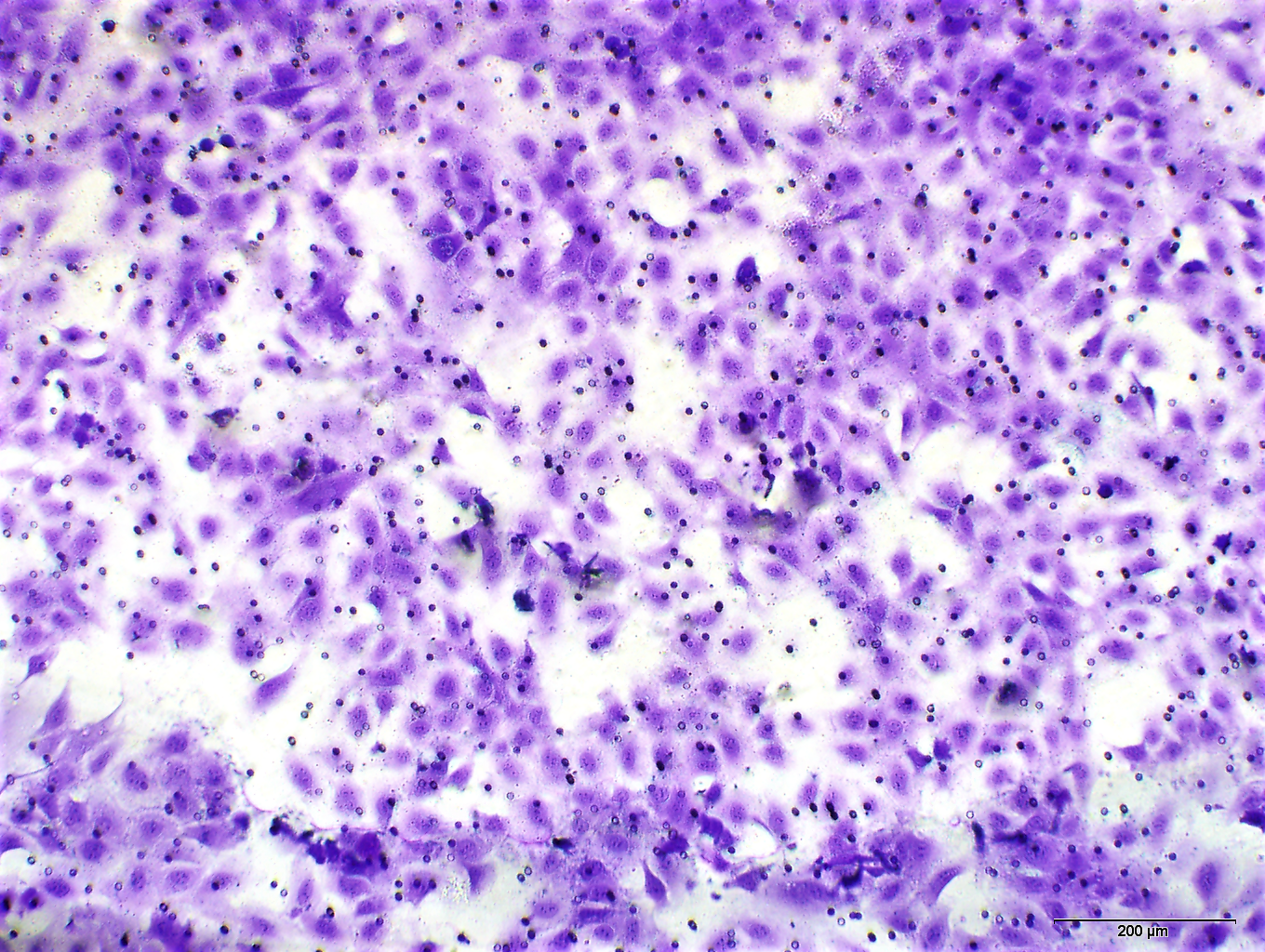

Supplement: Supplementary file 3 [file Data_Sheet_3.ZIP › Transwell figures-2/Transwell K1-PCsi-NC.tif]

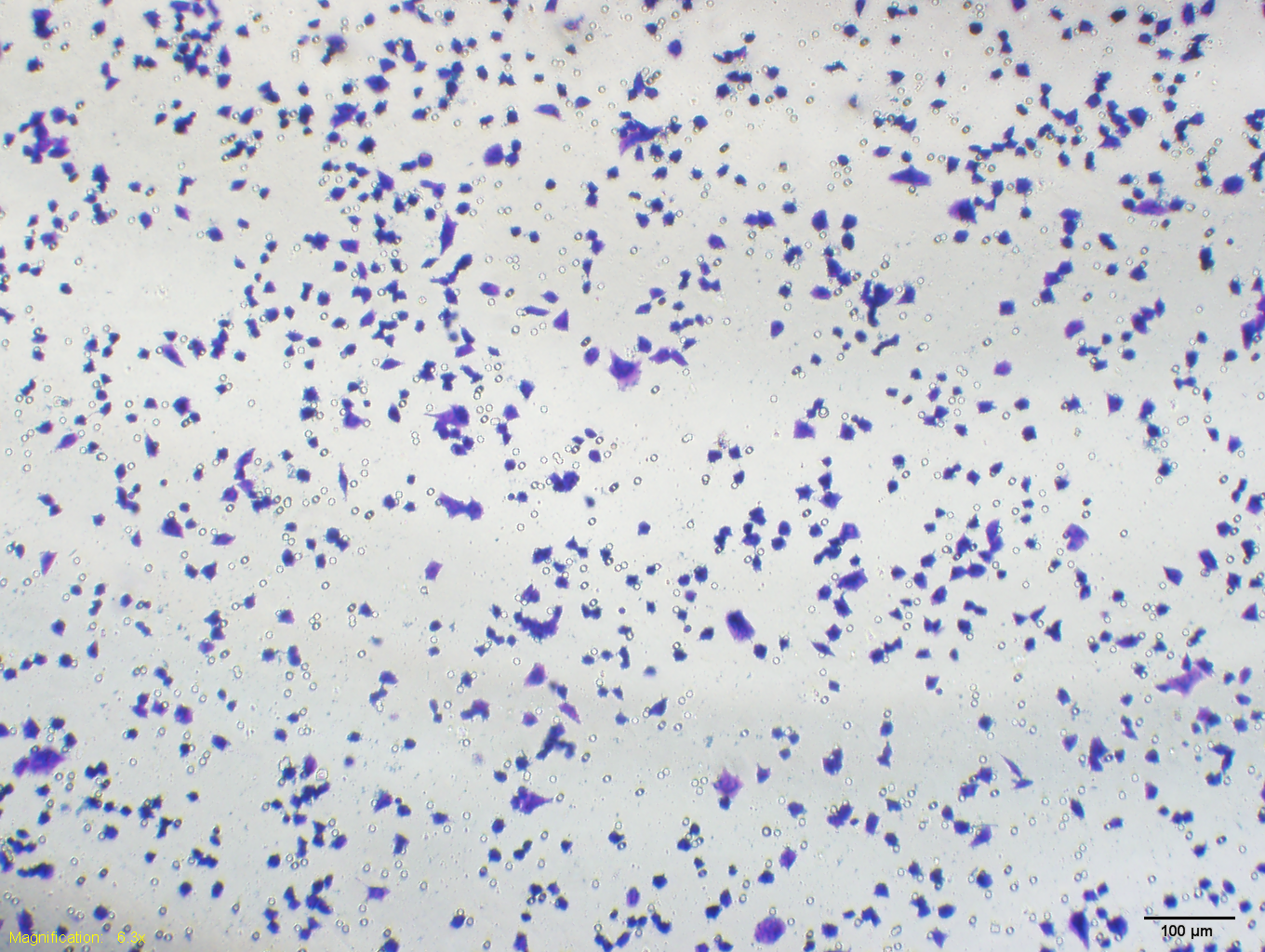

Supplement: Supplementary file 3 [file Data_Sheet_3.ZIP › Transwell figures-2/Transwell-BCPAP-prdm16NC-PC-NC.tif]

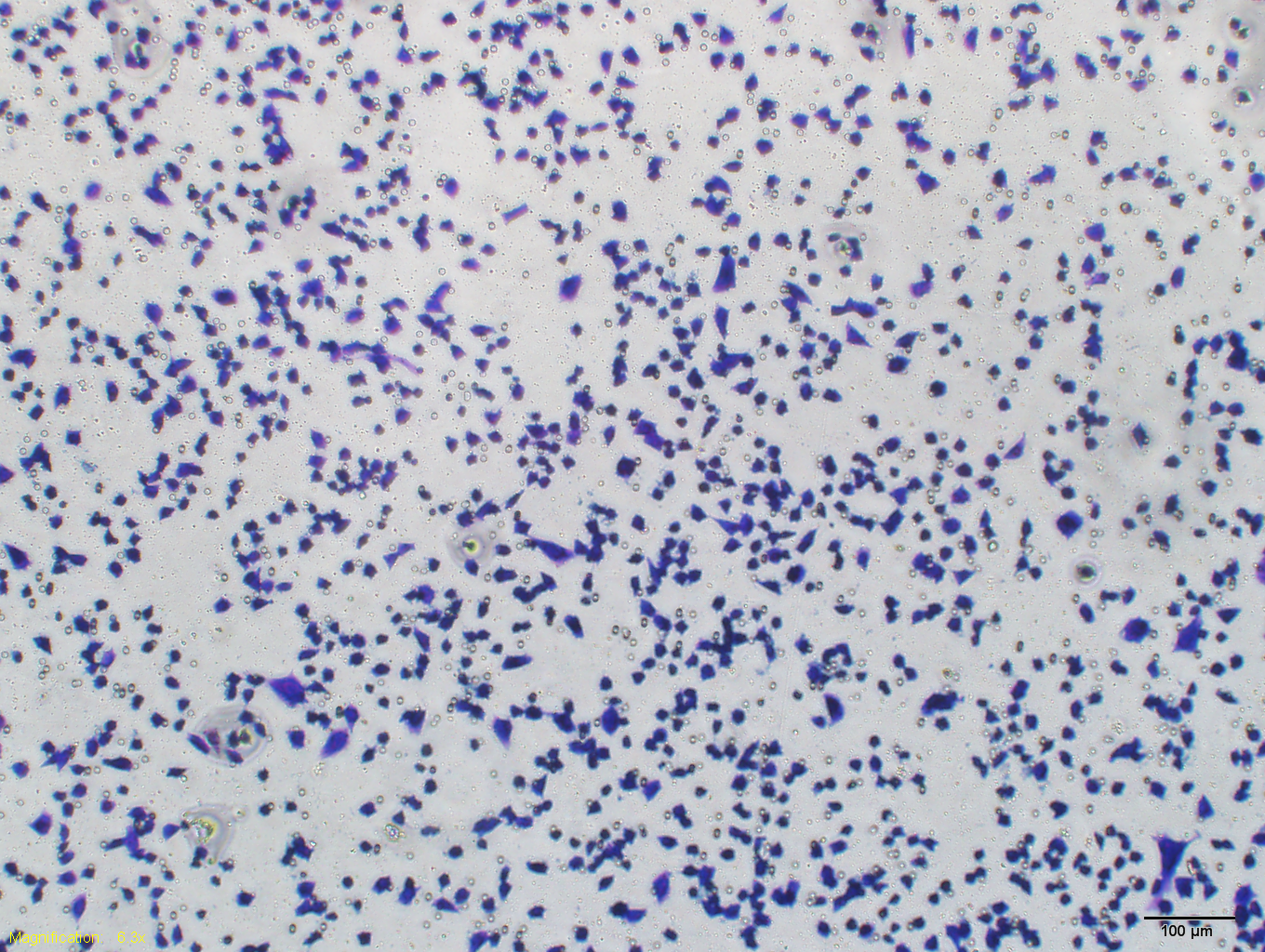

Supplement: Supplementary file 3 [file Data_Sheet_3.ZIP › Transwell figures-2/Transwell-BCPAP-prdm16nc-PC-OE.tif]

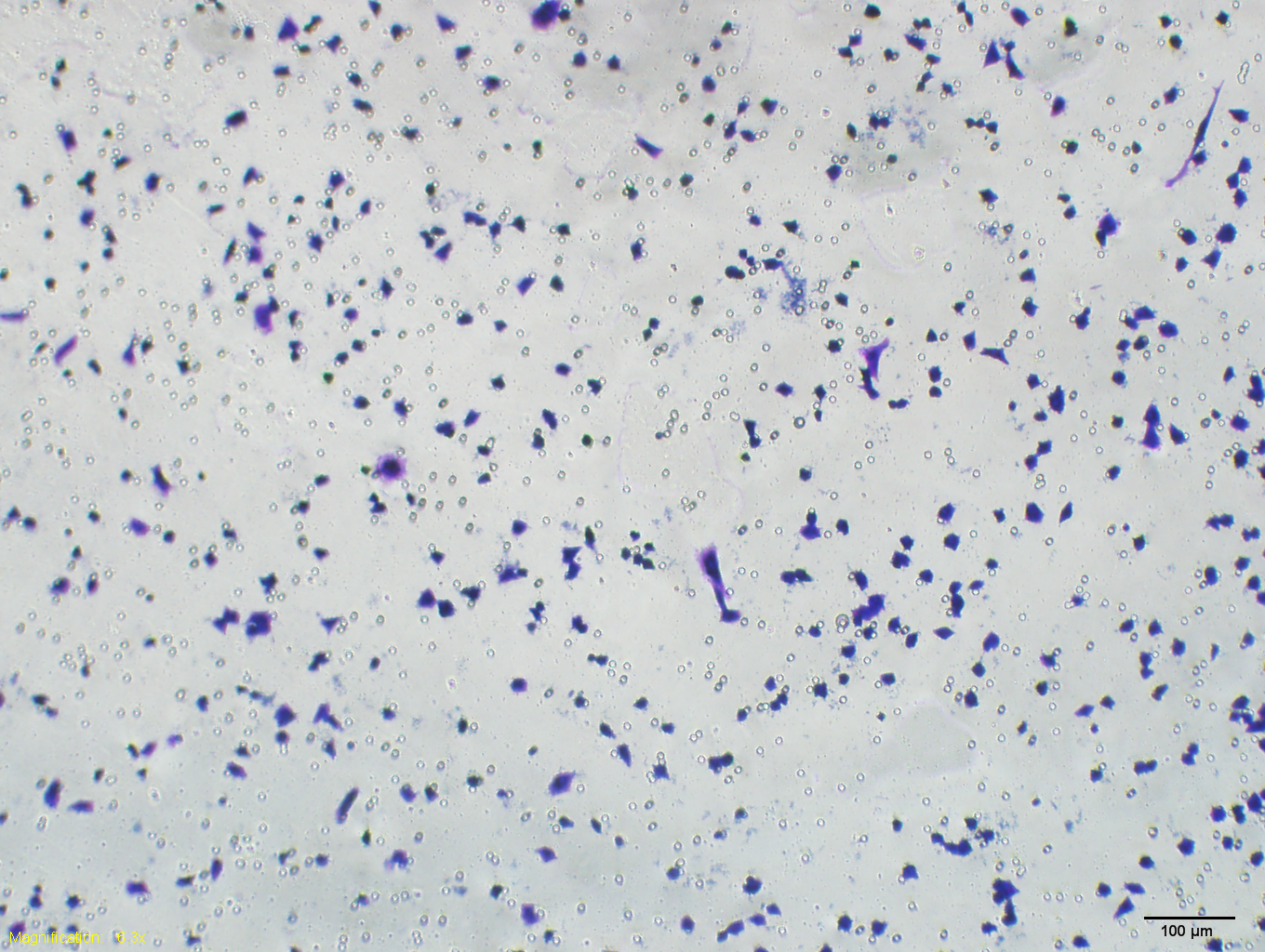

Supplement: Supplementary file 3 [file Data_Sheet_3.ZIP › Transwell figures-2/Transwell-BCPAP-prdm16OE-PC-NC.tif]

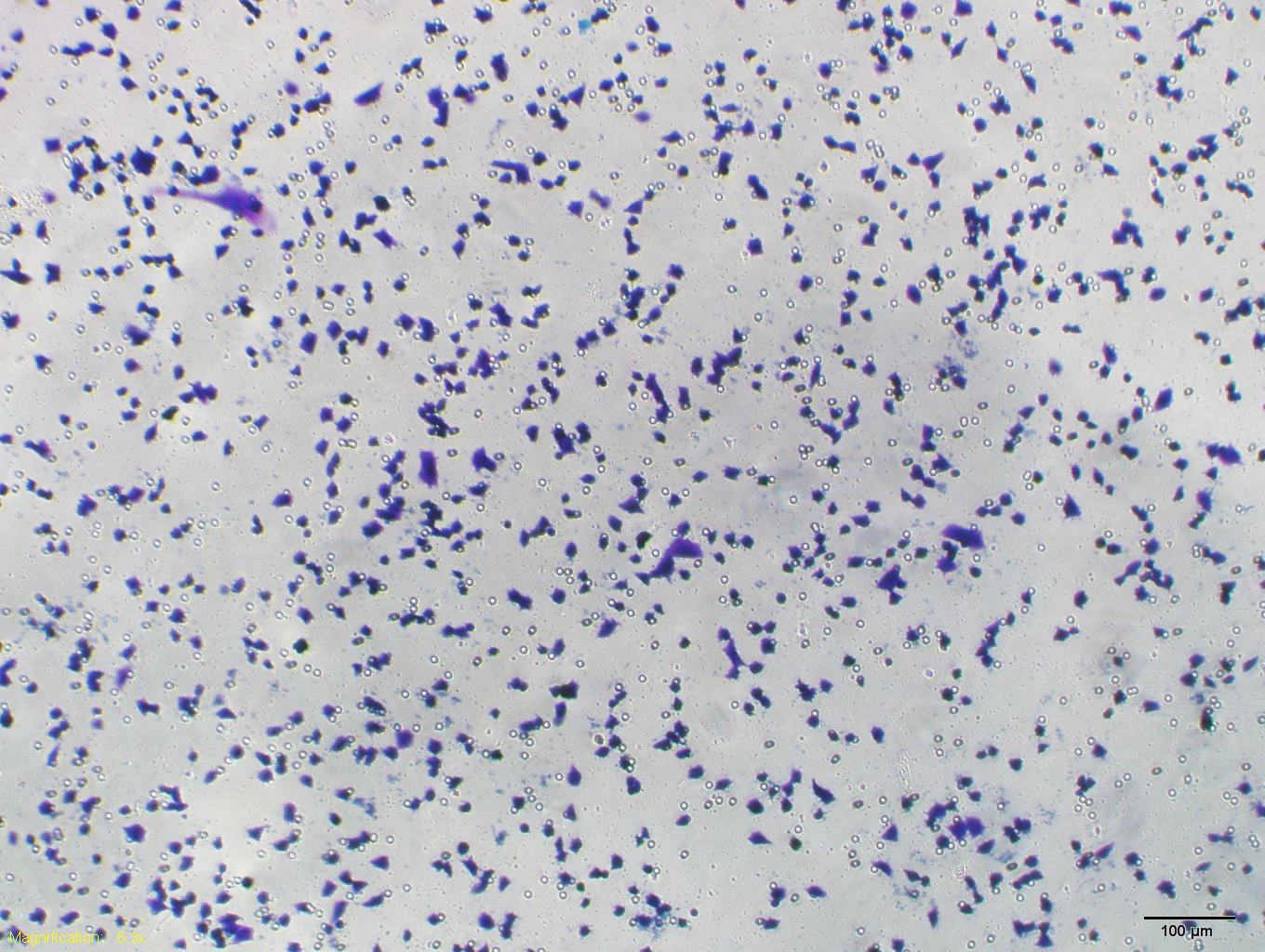

Supplement: Supplementary file 3 [file Data_Sheet_3.ZIP › Transwell figures-2/Transwell-BCPAP-prdm16OE-PC-OE.tif]

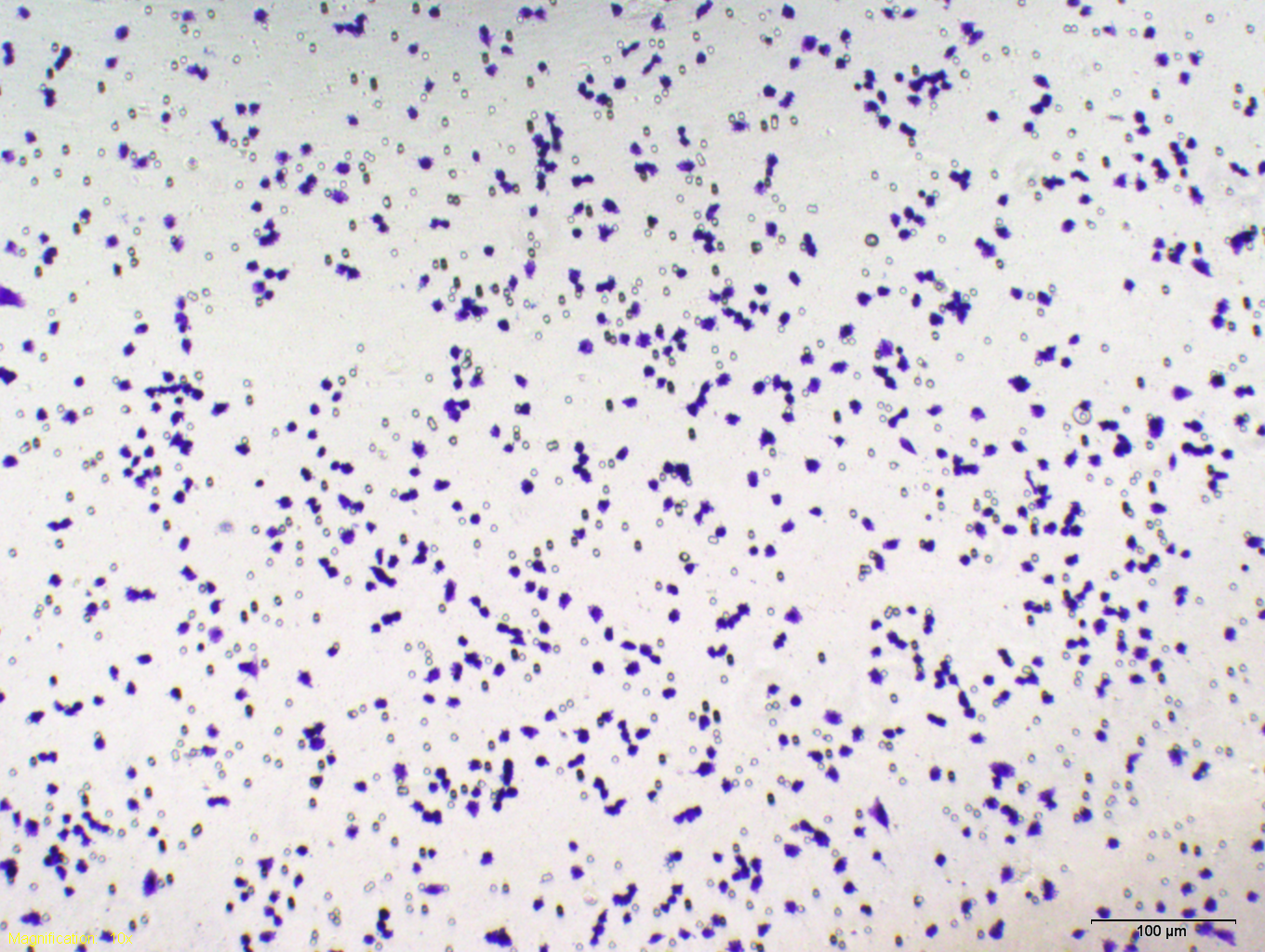

Supplement: Supplementary file 4 [file Data_Sheet_4.ZIP › Transwell figures-1/Transwell BCPAP-MOCK.tif]

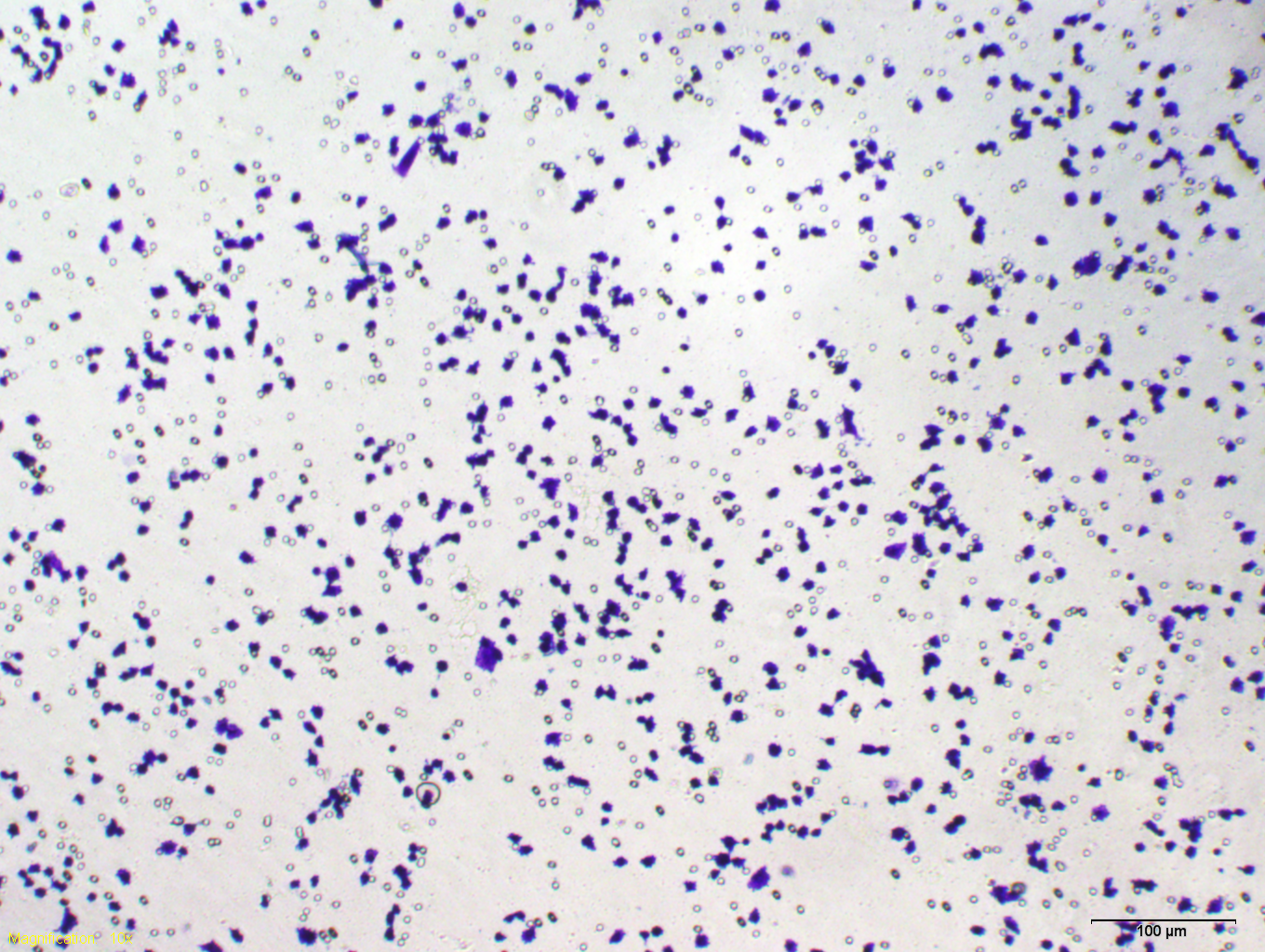

Supplement: Supplementary file 4 [file Data_Sheet_4.ZIP › Transwell figures-1/Transwell BCPAP-NC.tif]

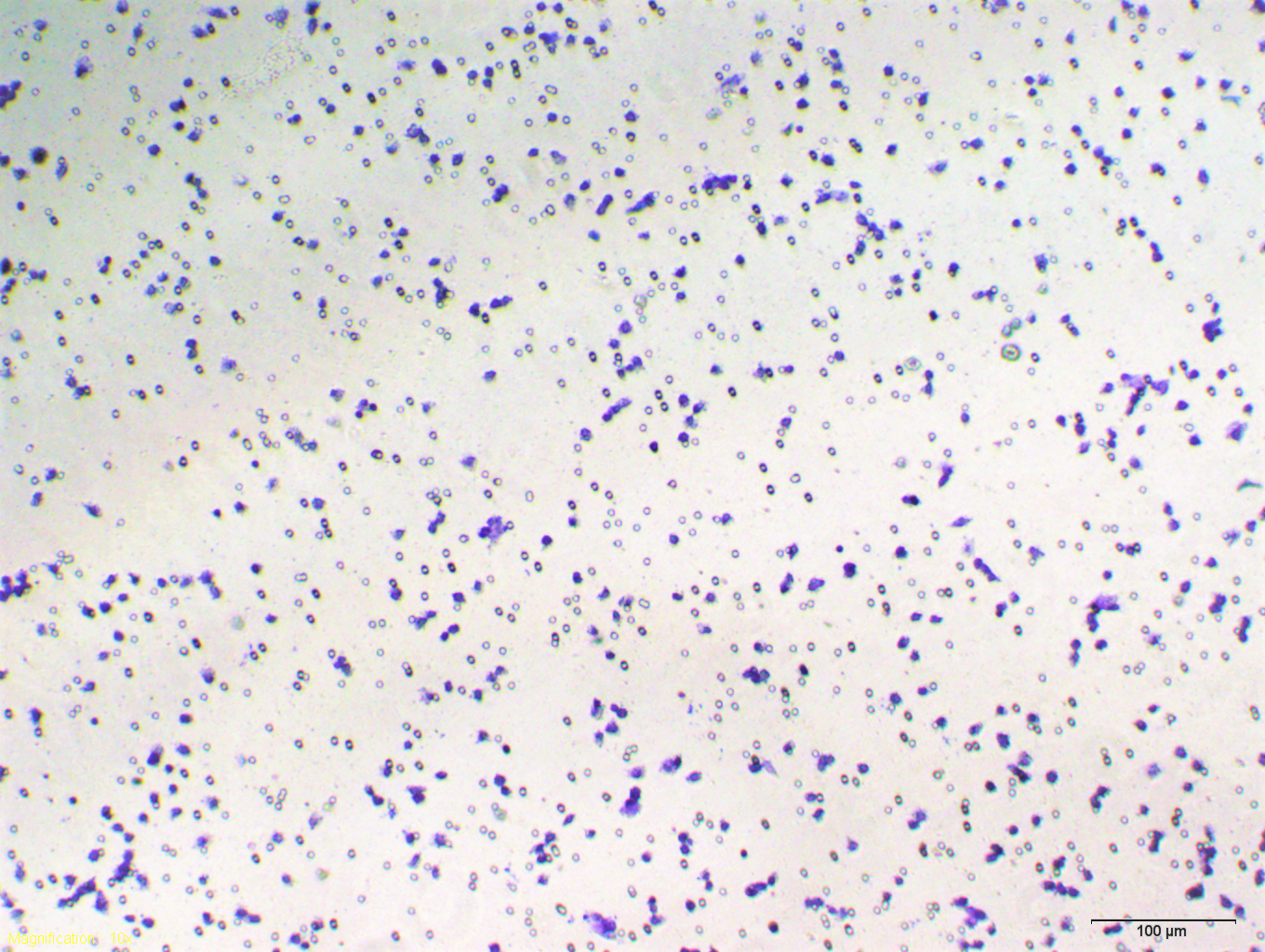

Supplement: Supplementary file 4 [file Data_Sheet_4.ZIP › Transwell figures-1/Transwell BCPAP-PRDM16OE.tif]

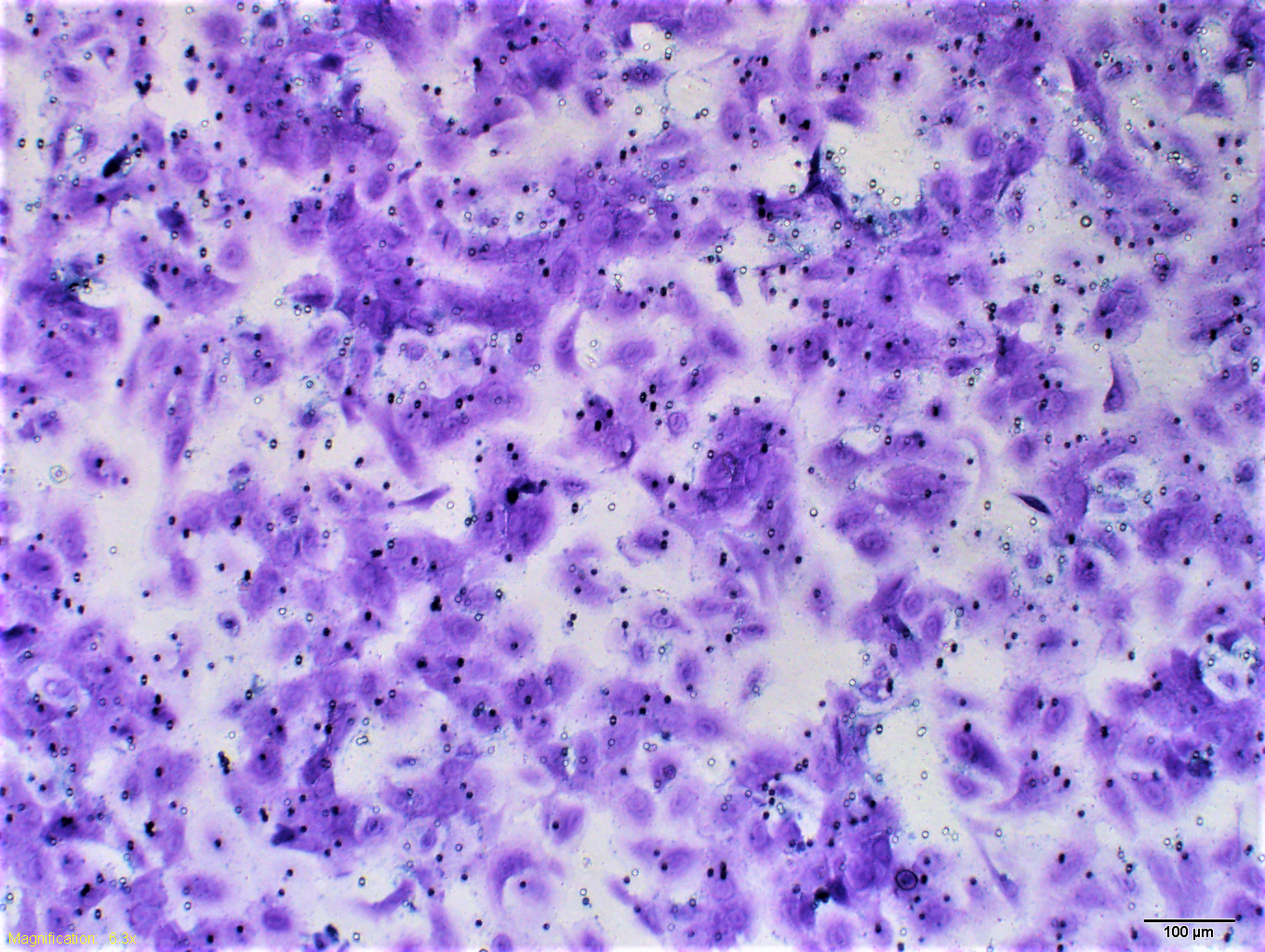

Supplement: Supplementary file 4 [file Data_Sheet_4.ZIP › Transwell figures-1/Transwell K1-MOCK.tif]

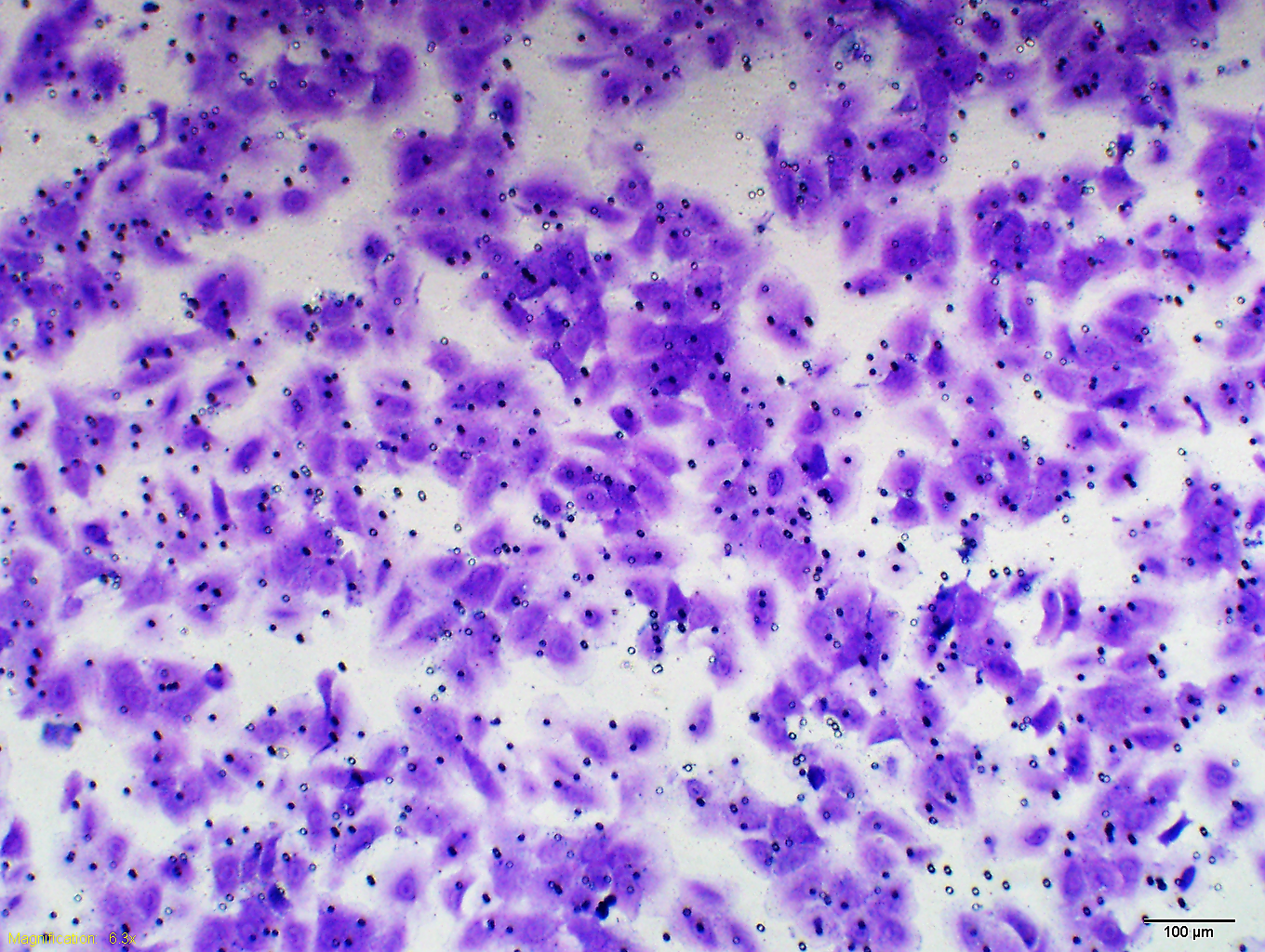

Supplement: Supplementary file 4 [file Data_Sheet_4.ZIP › Transwell figures-1/Transwell K1-NC.tif]

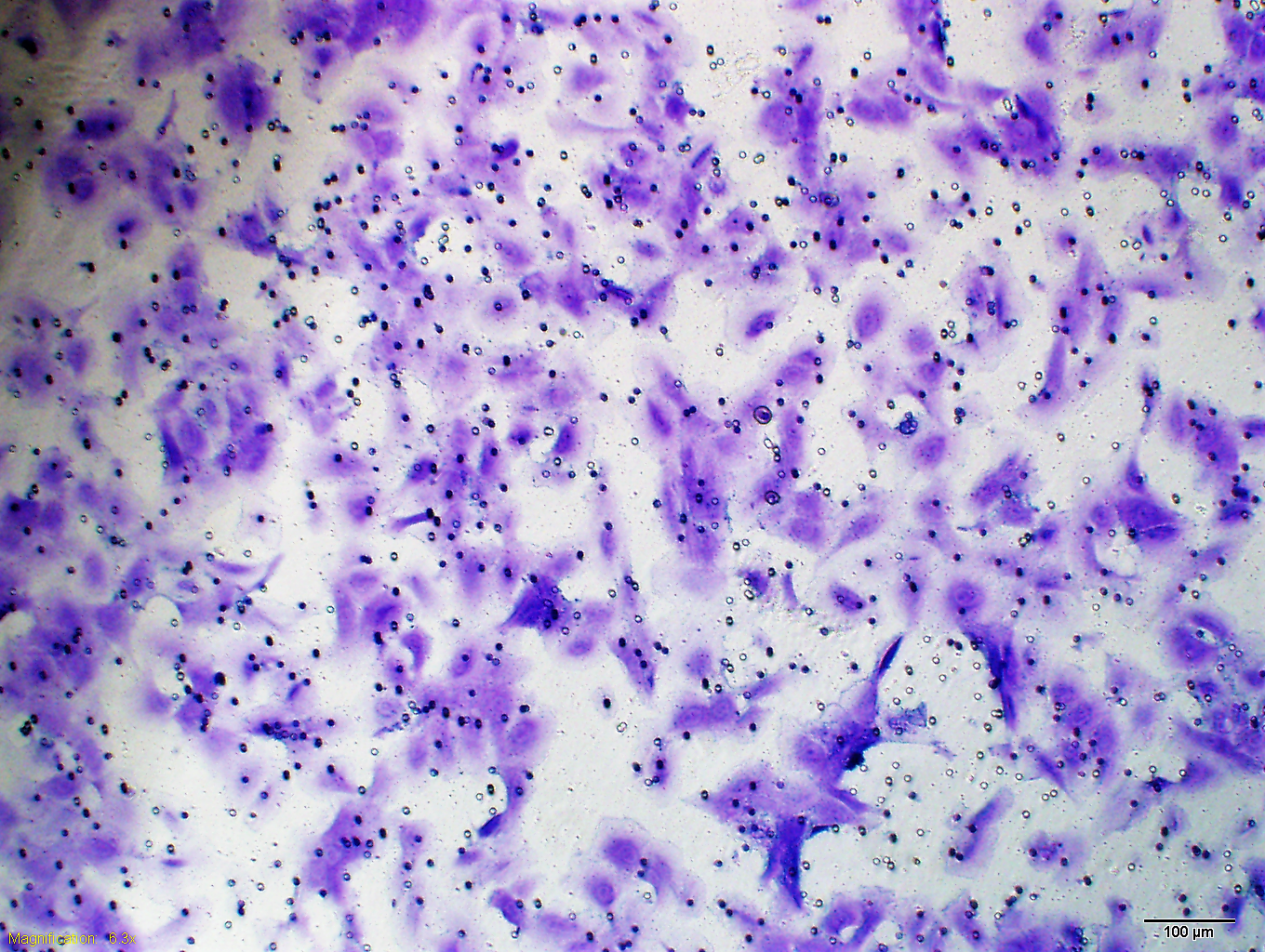

Supplement: Supplementary file 4 [file Data_Sheet_4.ZIP › Transwell figures-1/Transwell K1-PRDM16OE.tif]

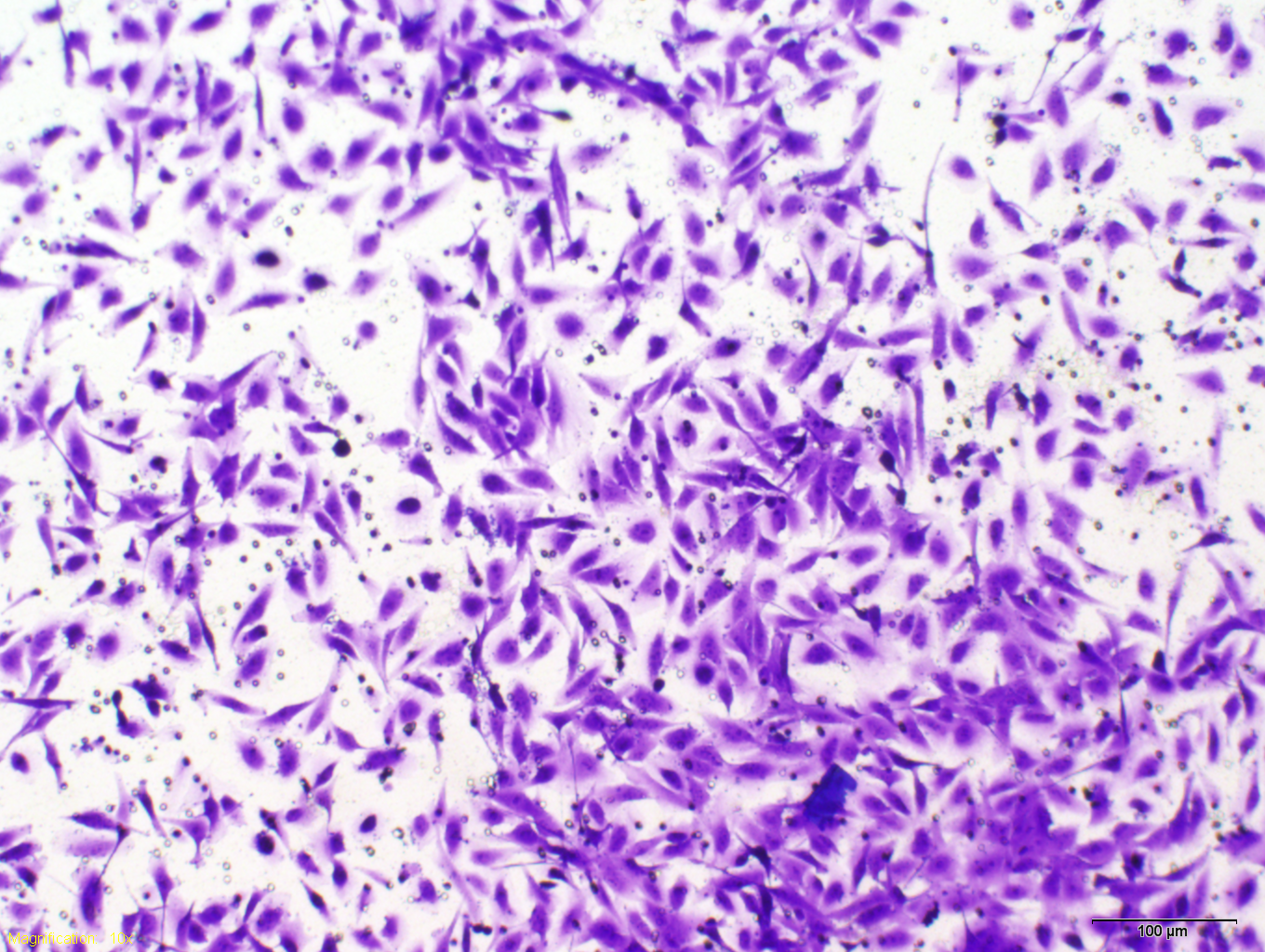

Supplement: Supplementary file 4 [file Data_Sheet_4.ZIP › Transwell figures-1/Transwell TPC-1-MOCK.tif]

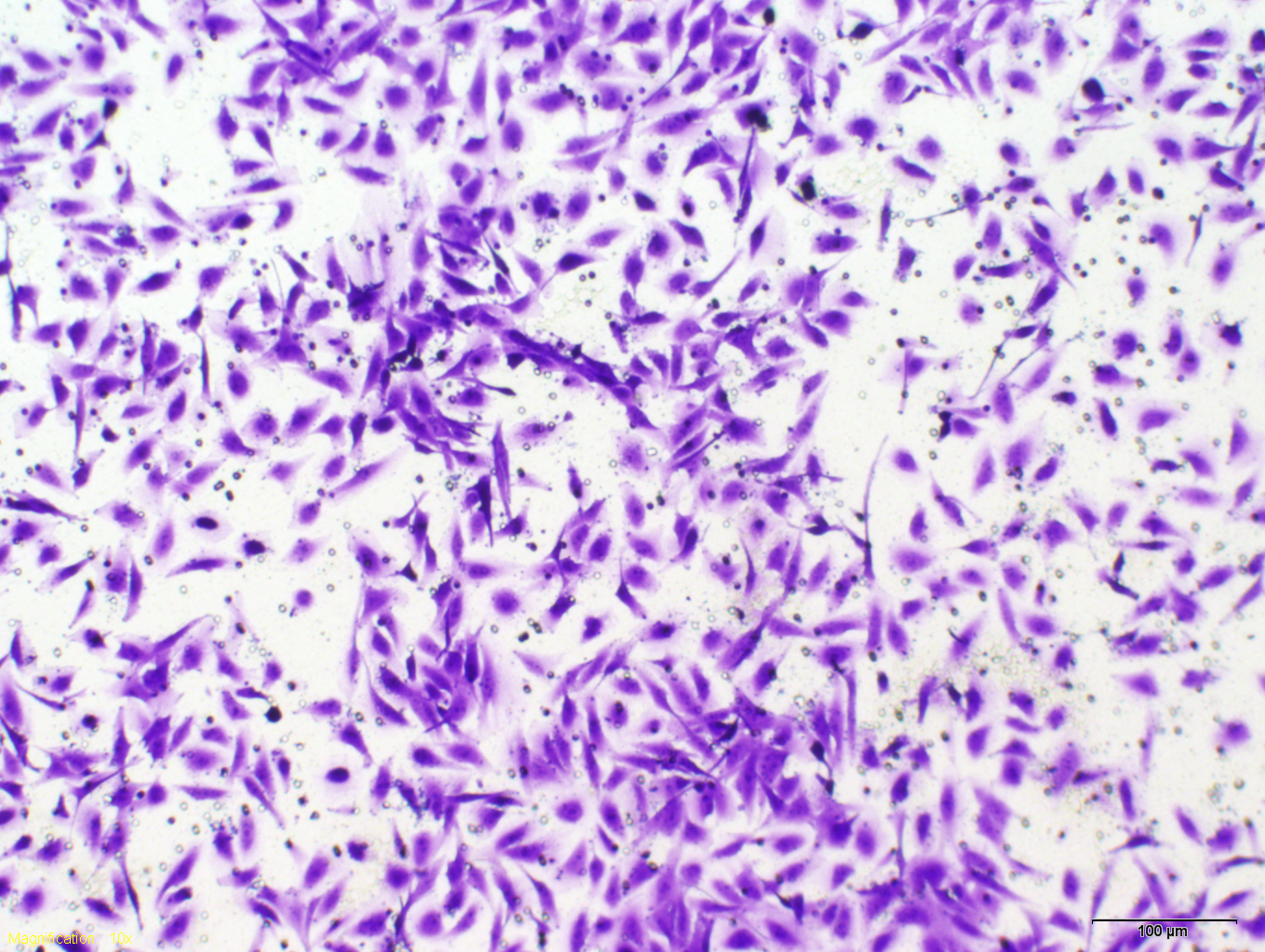

Supplement: Supplementary file 4 [file Data_Sheet_4.ZIP › Transwell figures-1/Transwell TPC-1-NC.tif]

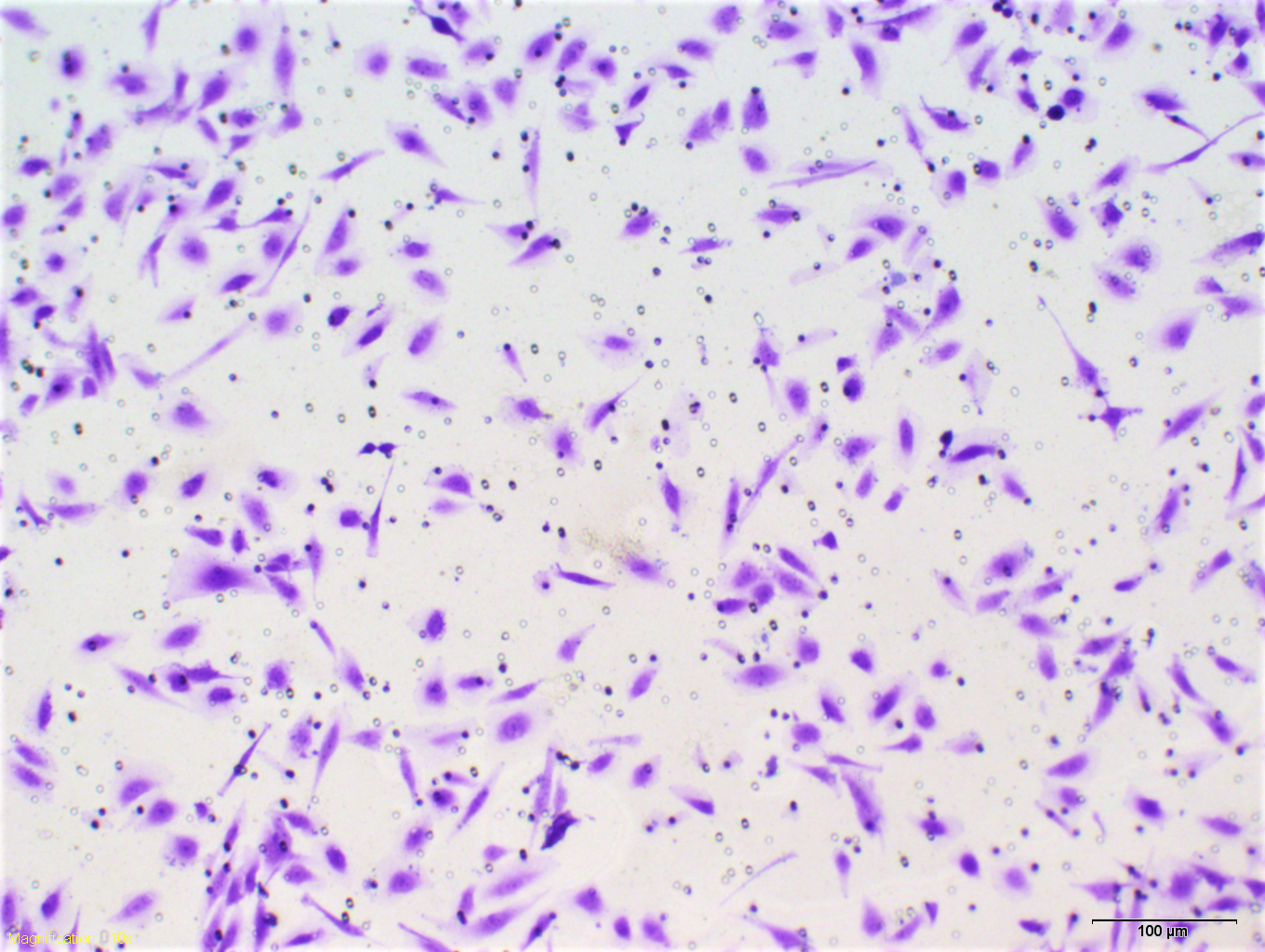

Supplement: Supplementary file 4 [file Data_Sheet_4.ZIP › Transwell figures-1/Transwell TPC-1-PRDM16OE.tif]

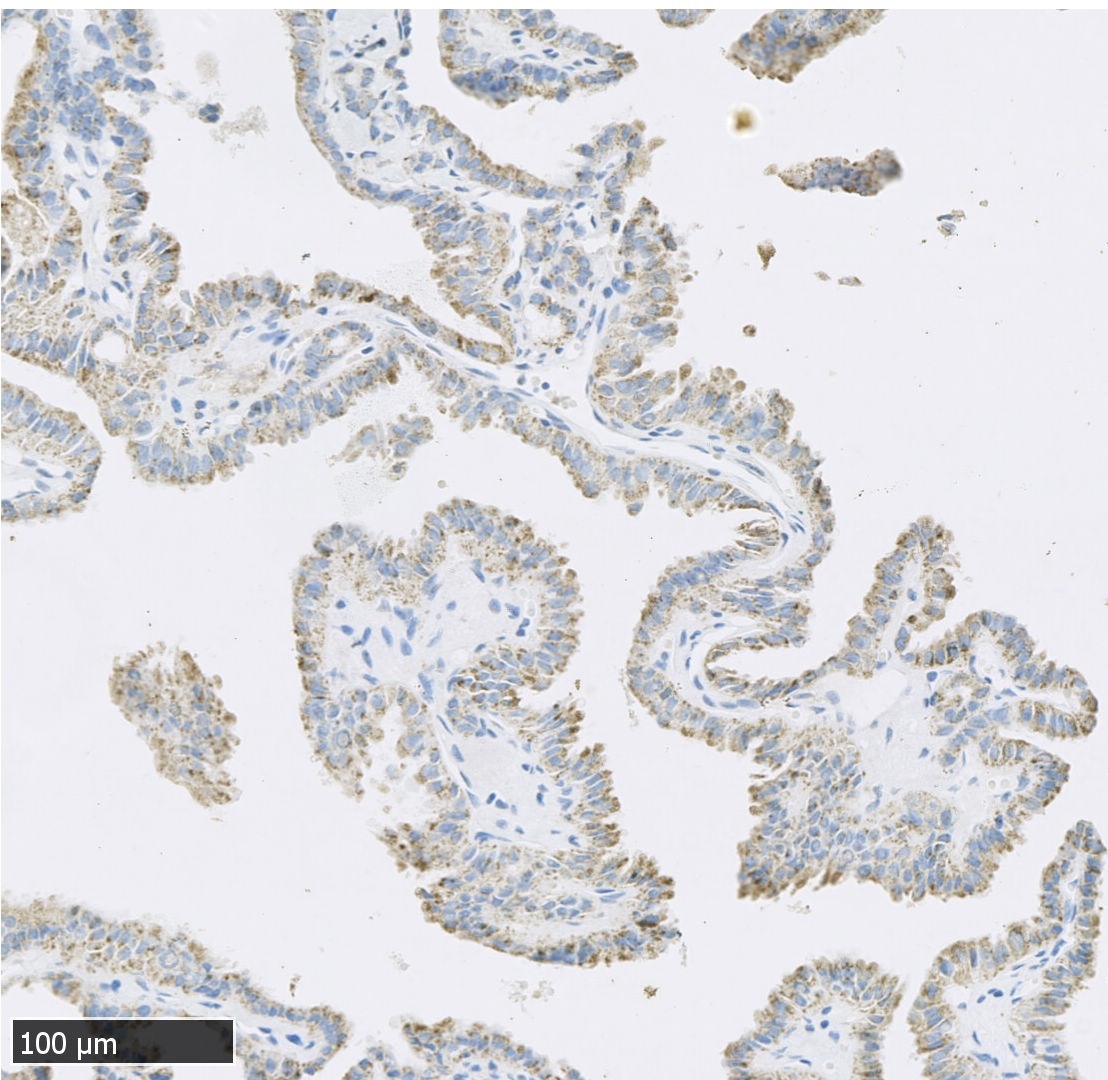

Supplement: Supplementary file 5 [file Data_Sheet_5.ZIP › IHC figures-1/IHC pc vs prdm16-PC.jpg]

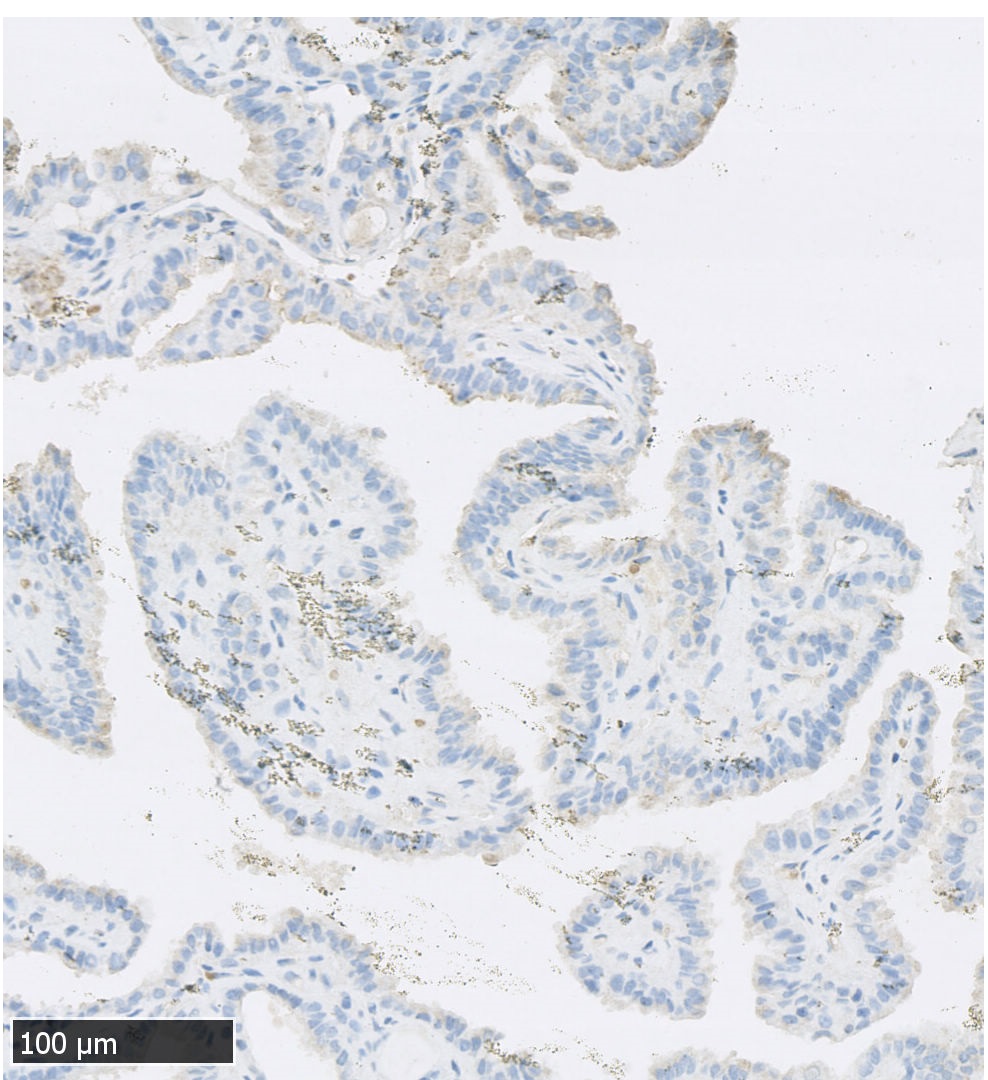

Supplement: Supplementary file 5 [file Data_Sheet_5.ZIP › IHC figures-1/IHC pc vs prdm16-PR16.jpg]

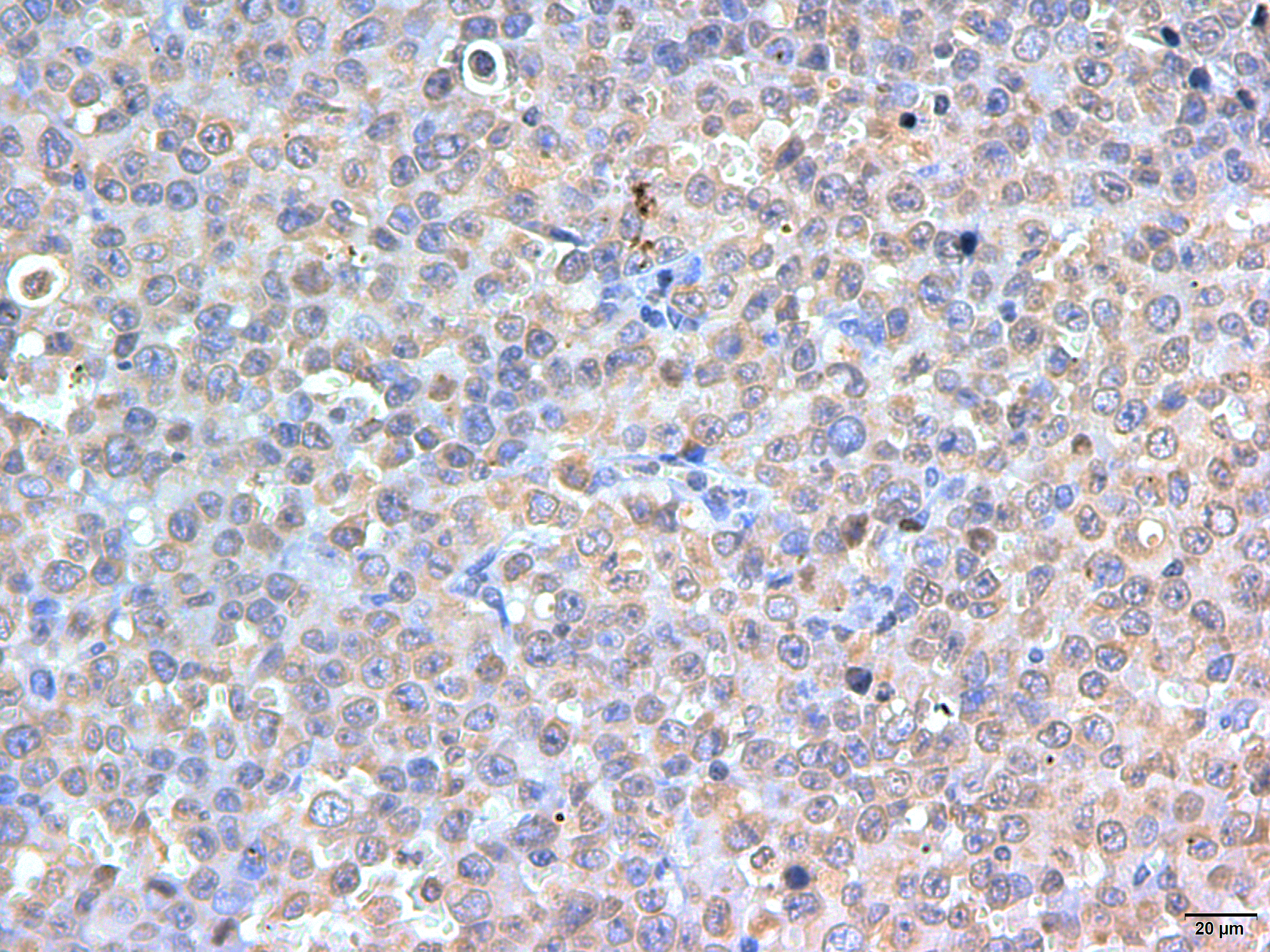

Supplement: Supplementary file 5 [file Data_Sheet_5.ZIP › IHC figures-1/IHC-MOUSE-PC-PRDM16OE.tif]

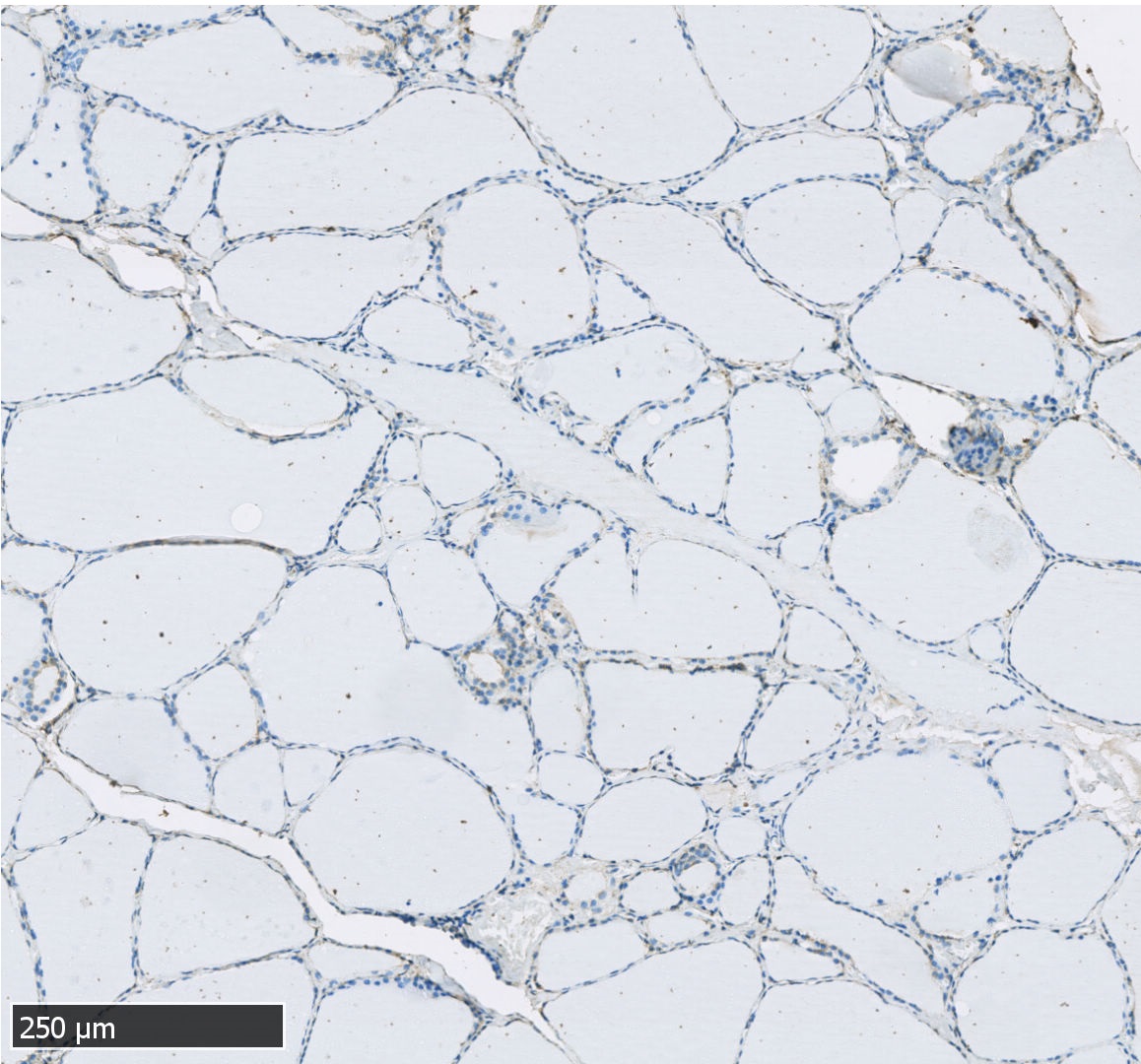

Supplement: Supplementary file 5 [file Data_Sheet_5.ZIP › IHC figures-1/IHC-PC-PT.jpg]

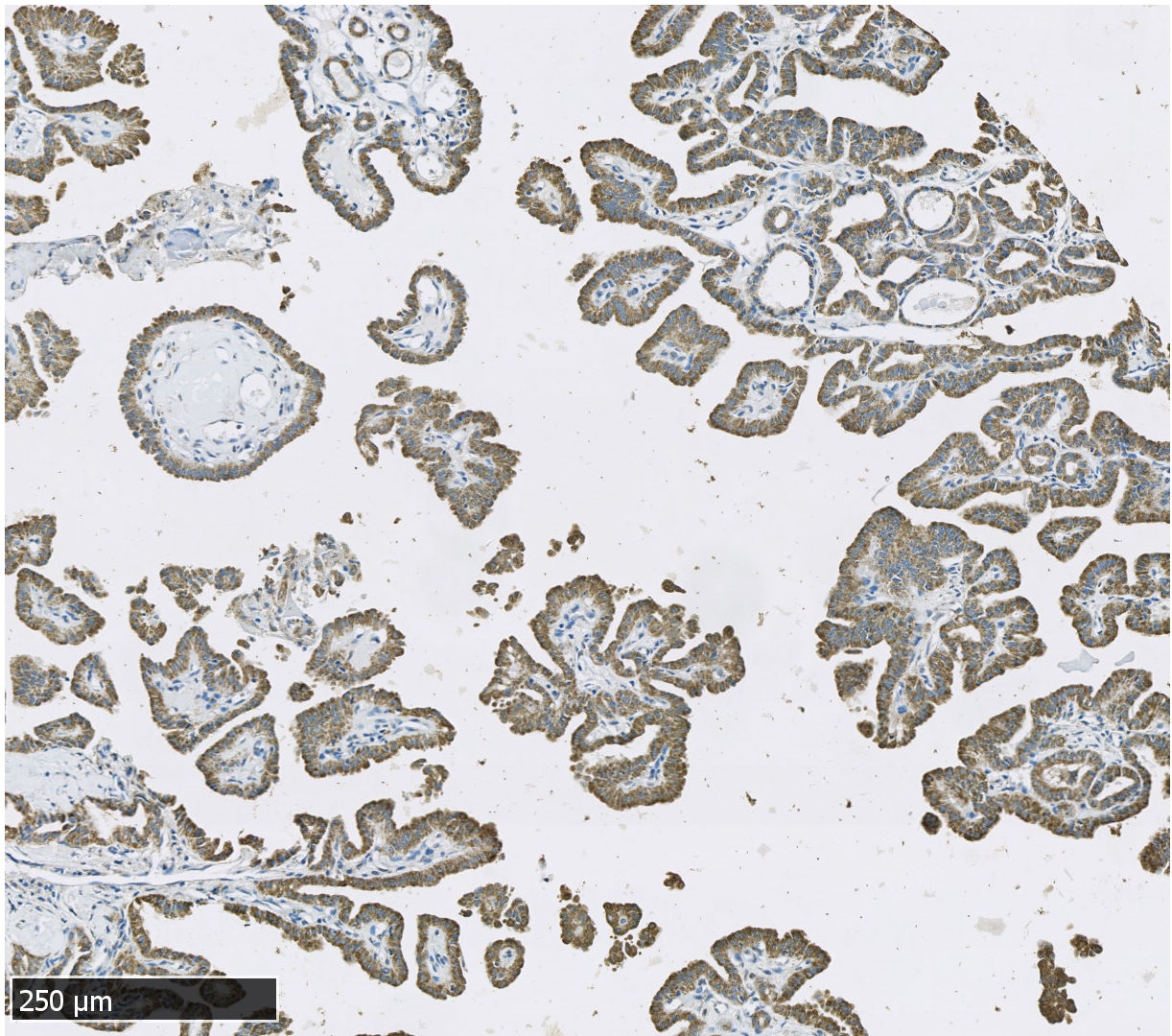

Supplement: Supplementary file 5 [file Data_Sheet_5.ZIP › IHC figures-1/IHC-PC-T.jpg]

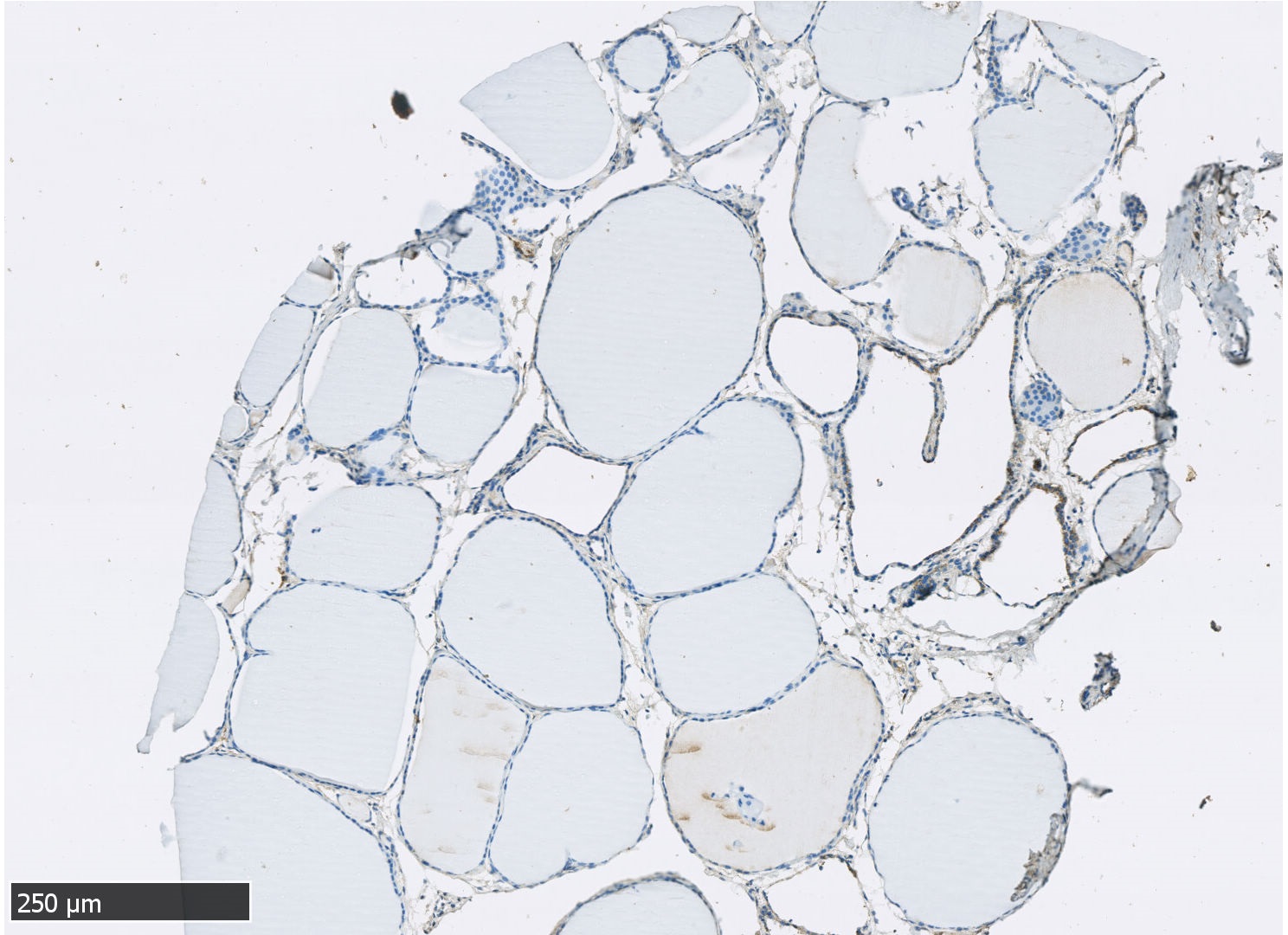

Supplement: Supplementary file 5 [file Data_Sheet_5.ZIP › IHC figures-1/IHC-PRDM16-PT.jpg]

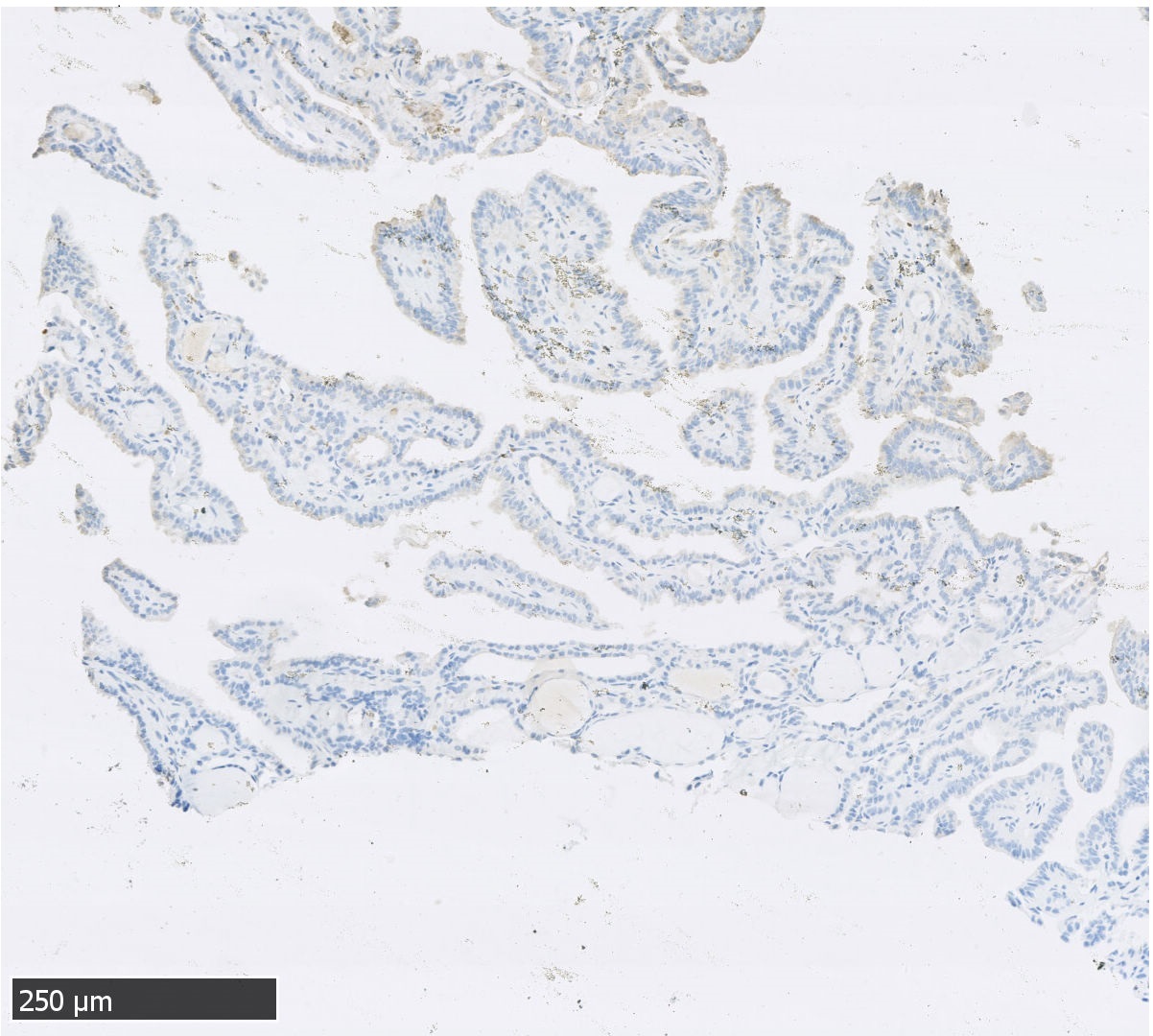

Supplement: Supplementary file 5 [file Data_Sheet_5.ZIP › IHC figures-1/IHC-PRDM16-T.jpg]

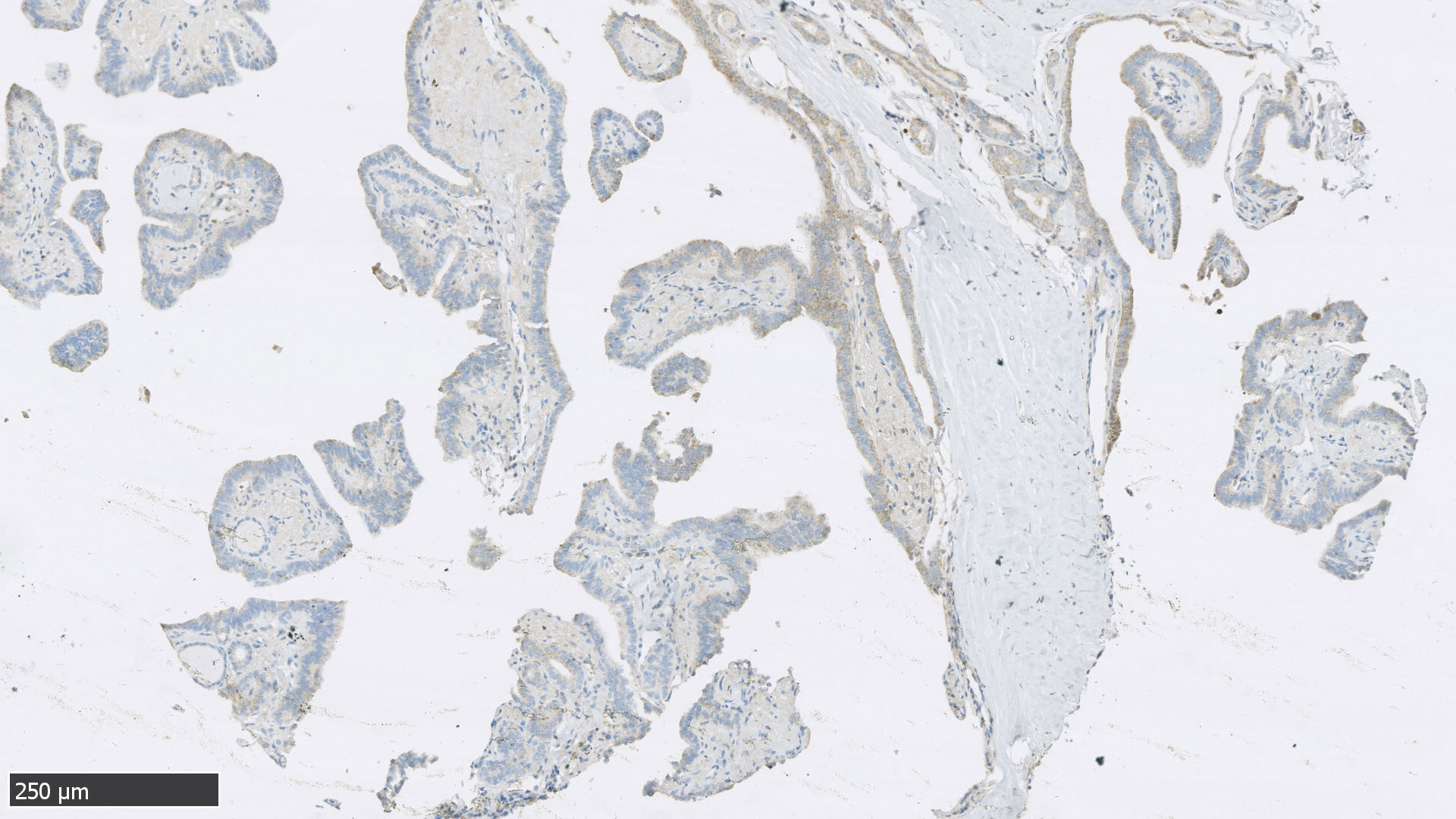

Supplement: Supplementary file 5 [file Data_Sheet_5.ZIP › IHC figures-1/pc-prdm16-PR16.jpg]

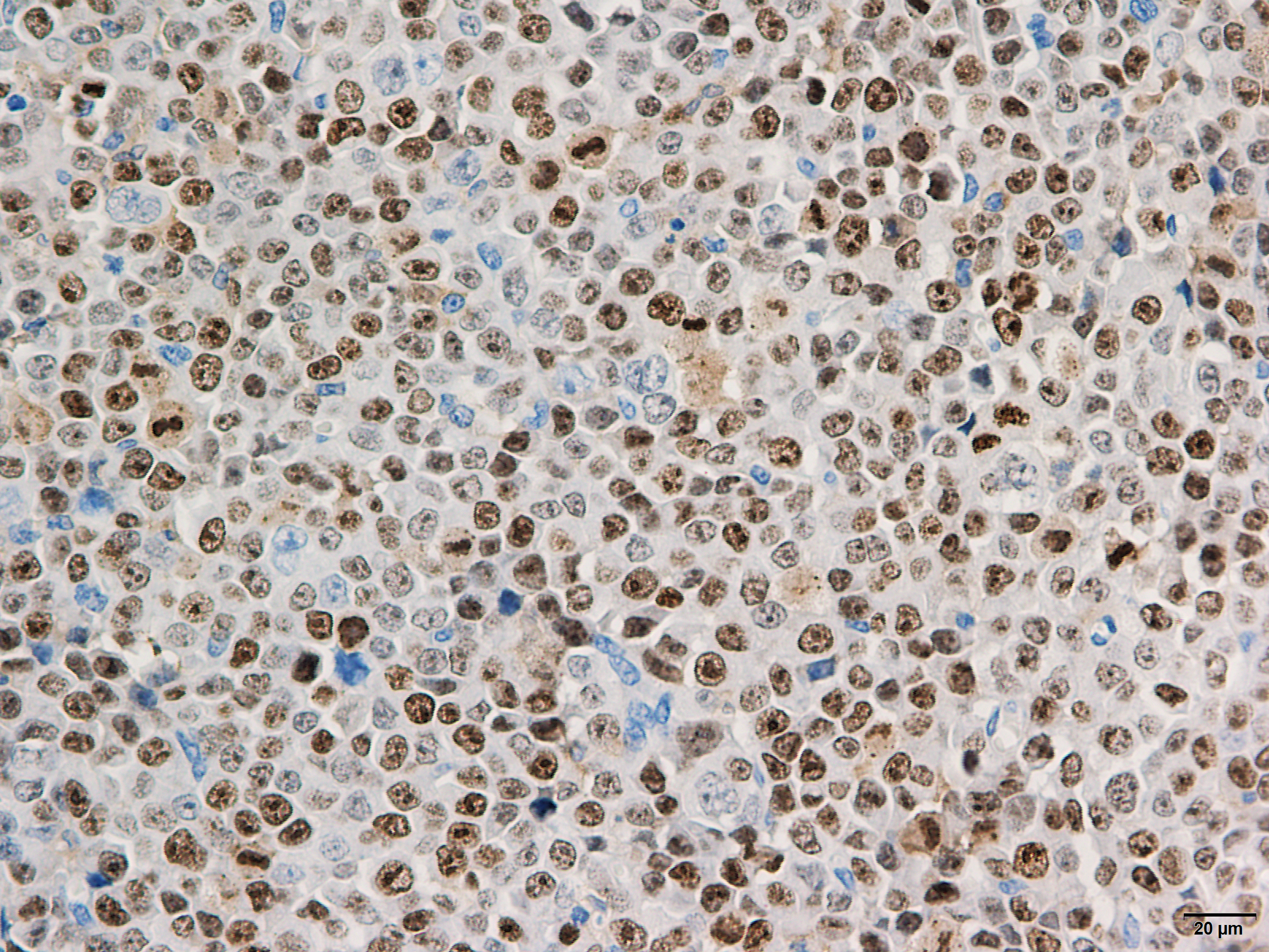

Supplement: Supplementary file 6 [file Data_Sheet_6.ZIP › IHC figures-2/IHC-ki67-control.tif]

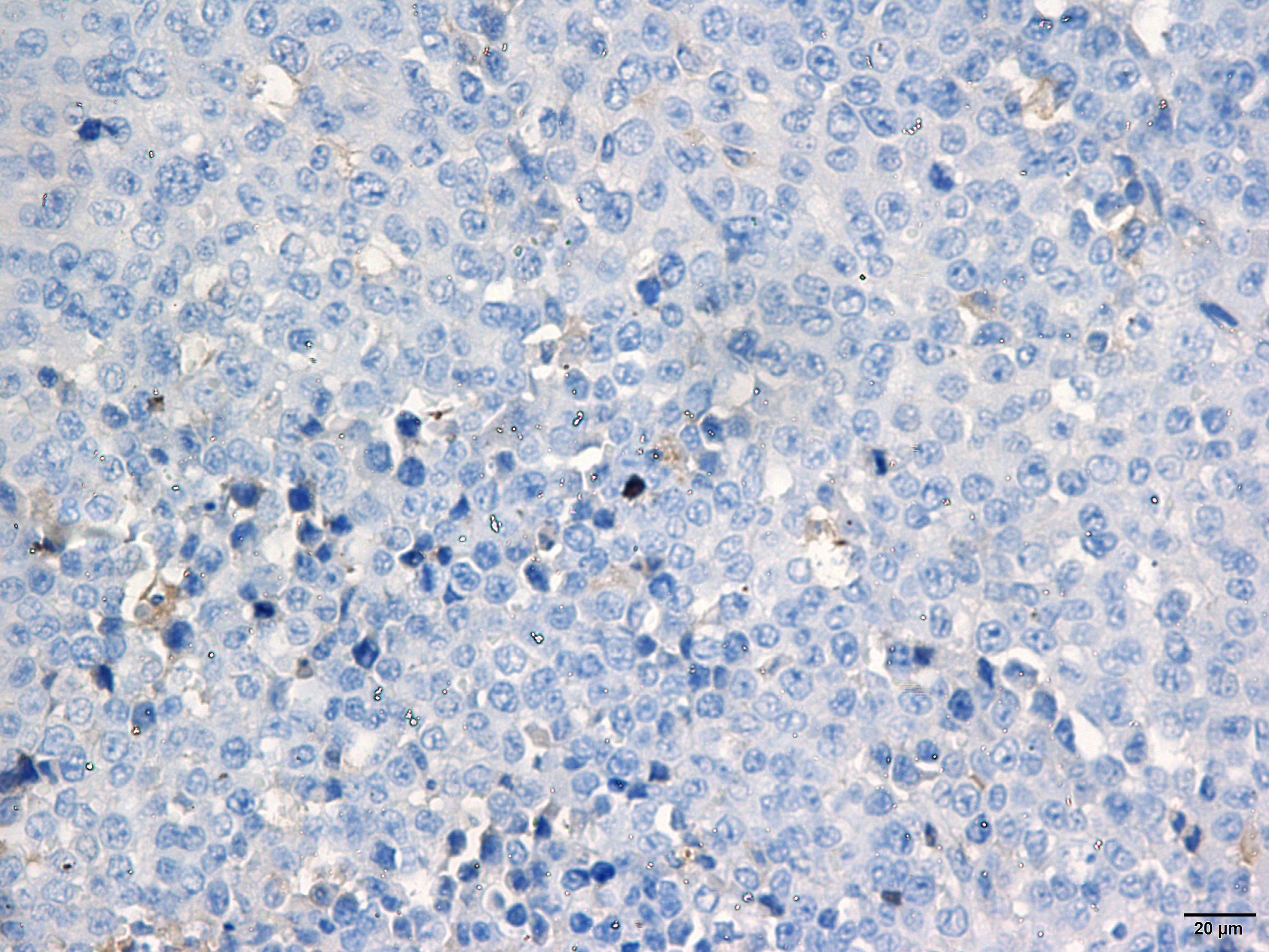

Supplement: Supplementary file 6 [file Data_Sheet_6.ZIP › IHC figures-2/IHC-MOUSE-PRDM16-PRDM16NC.tif]

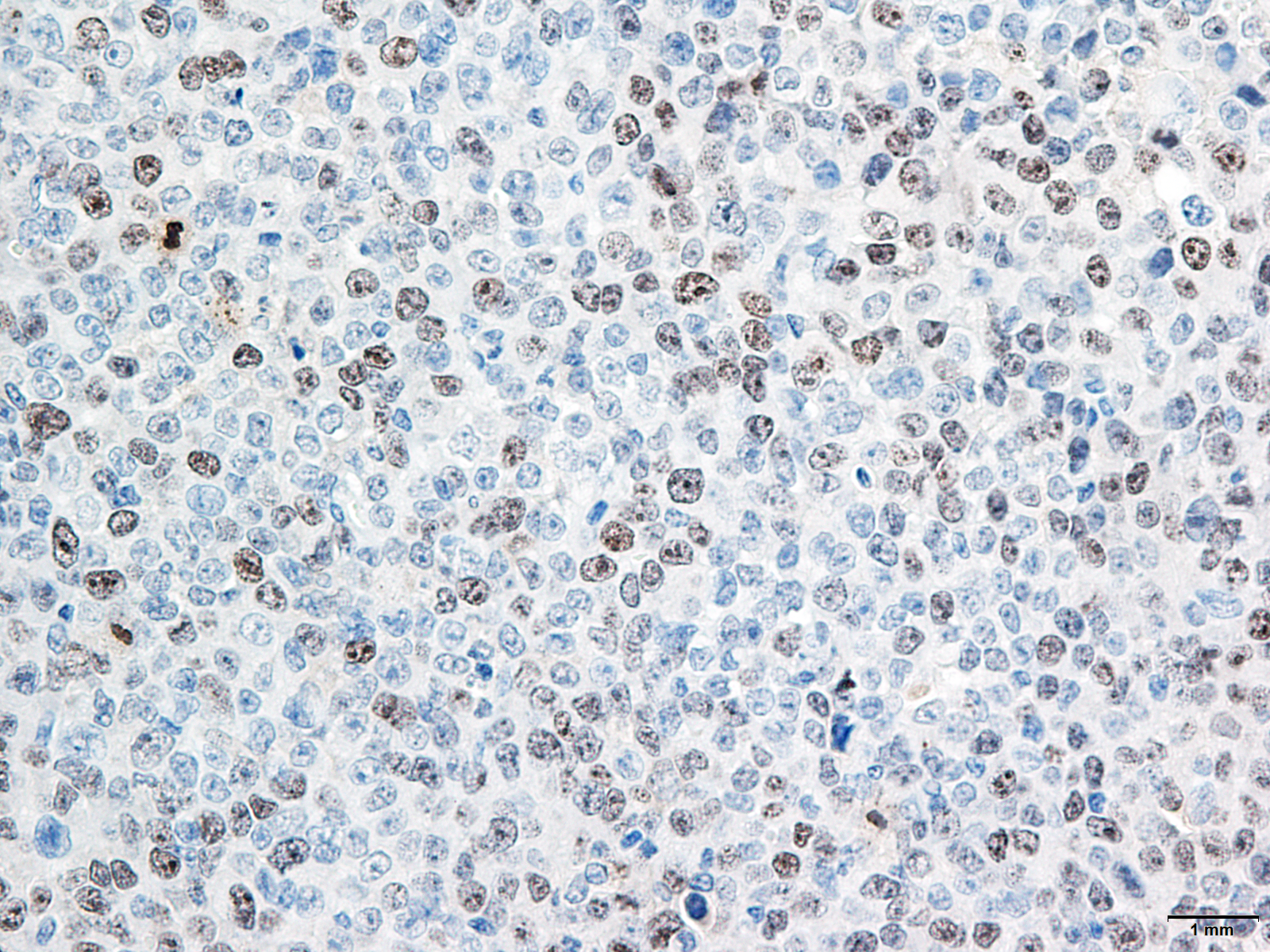

Supplement: Supplementary file 7 [file Data_Sheet_7.ZIP › IHC figures-3/IHC ki67-PRDM16OE.tif]

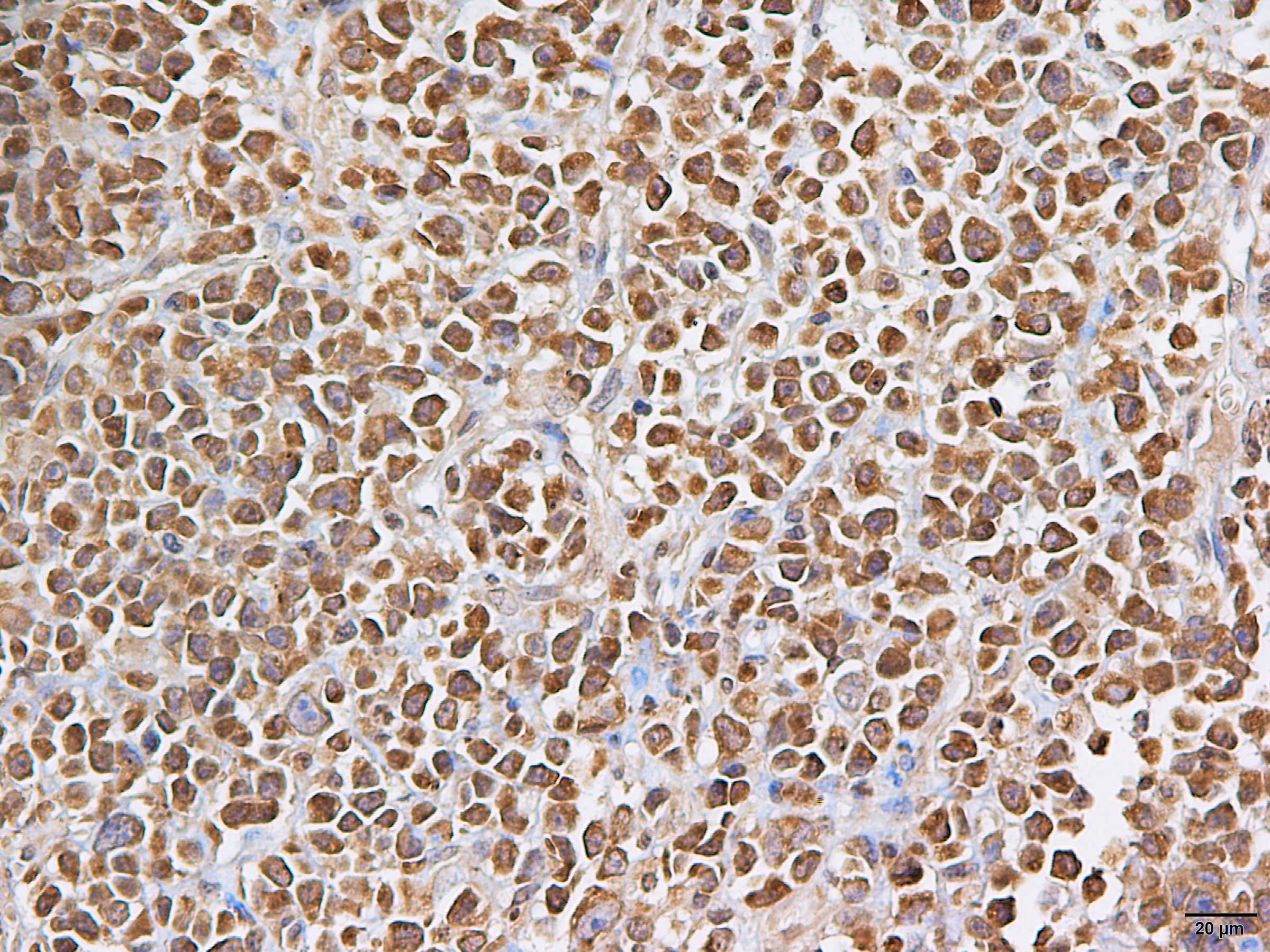

Supplement: Supplementary file 7 [file Data_Sheet_7.ZIP › IHC figures-3/IHC-MOUSE-PC-PRDM16NC.tif]

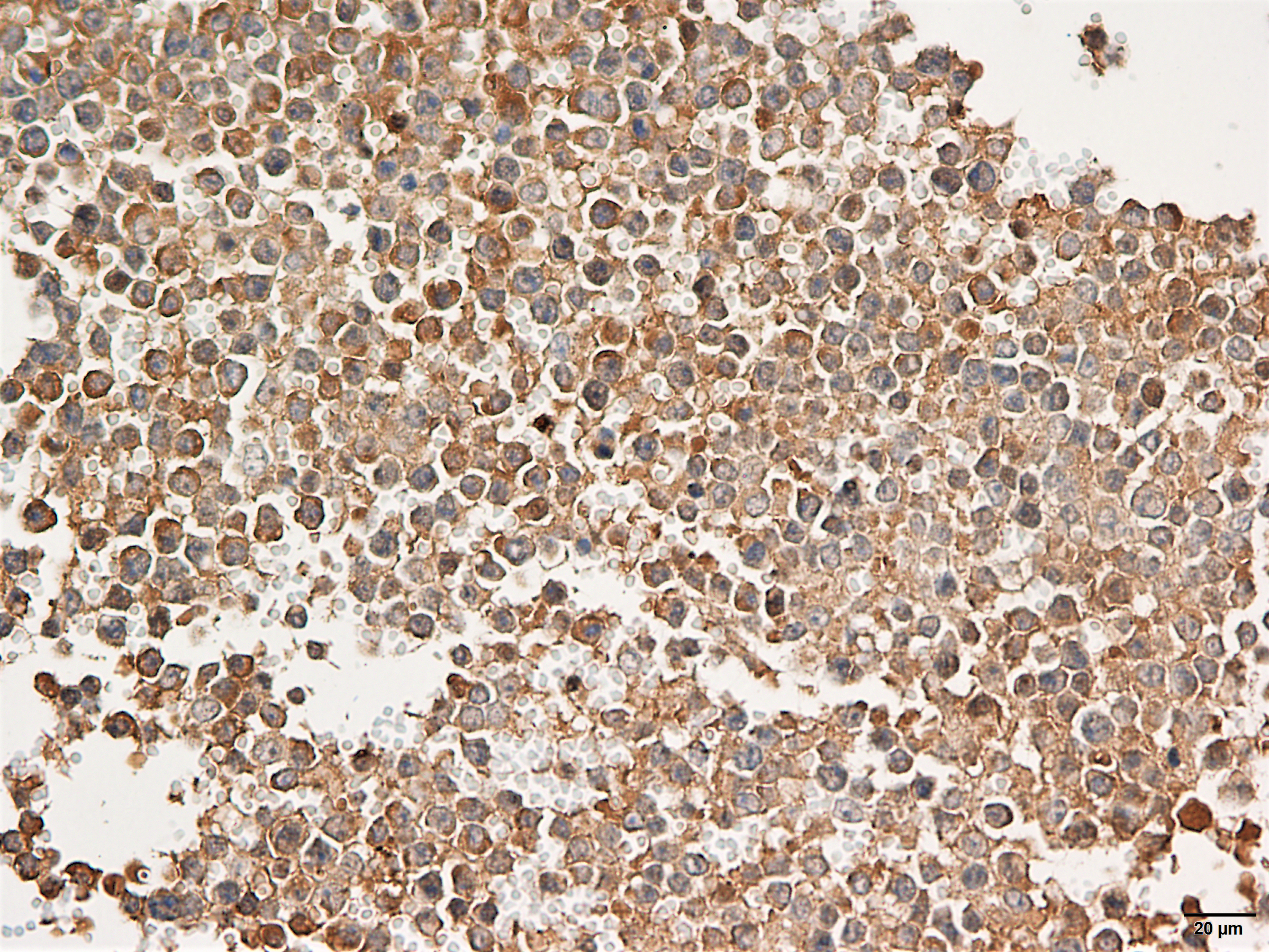

Supplement: Supplementary file 7 [file Data_Sheet_7.ZIP › IHC figures-3/IHC-MOUSE-PRDM16-PRDM16OE.tif]

Figure S1

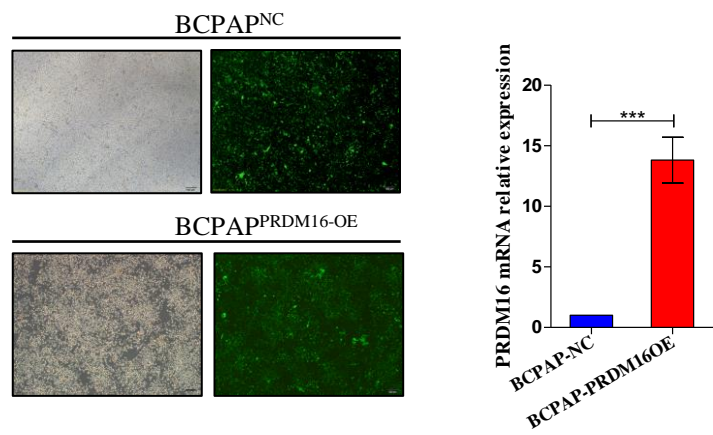

Figure S2

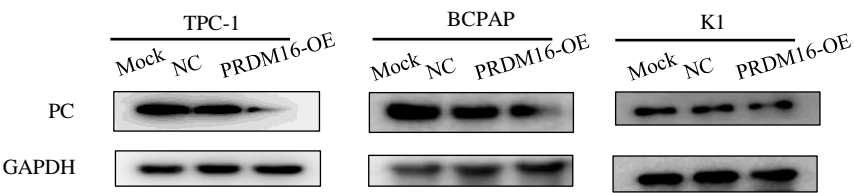

Supplement: Supplementary file 9 [file Data_Sheet_9.pdf]
