# Supplementary material for: PRDM16 Inhibits Cell Proliferation and Migration via Epithelial-to-Mesenchymal Transition by Directly Targeting Pyruvate Carboxylase in Papillary Thyroid Cancer
Source: Front Cell Dev Biol. 2021 Nov 2;9:723777. doi: 10.3389/fcell.2021.723777 (PMC8593917; doi:10.3389/fcell.2021.723777)
Supplement: Supplementary file 8 [file Data_Sheet_8.PDF]

Supplementary tables

Table S1. Primers used for quantative real-time PCR

Gene	Forward (5’-3’)	Reverse (5’-3’)
β-actin	TGACGTGGACATCCGCAAAG	CTGGAAGGTGGACAGCGAGG
PRDM16	AGGCTGCATCACAAAGATCTCC	CCTCACCTGGCTCAATGTCC
E-cadherin	GAAGGTGACAGAGCCTCTGGAT	GATCGGTTACCGTGATCAAAATC
N-cadherin	AGGGGACCTTTTCCTCAAGA	CAATGTCAATGGGGTTCTCC
Vimentin	GGACCAGCTAACCAACGACA	AAGGTCAAGACGTGCCAGAG
MMP3	CAGGCTTTCCCAAGCAAATA	GTGCCCATATTGTGCCTTCT
RRAD	CCTGGTGCGCTCTCGTGAGGT	GTGCCTGCTTGCCGTCGTG
PTGES	TCCTAACCCTTTTGTCGCCTG	CGCTTCCCAGAGGATCTGC
PIM1	CCCGACAGTTTCGTCCTGAT	ACCCGAAGTCGATGAGCTTG
LYPD3	AAGAATGACCGCGGCCTGGATC	GACATGATCGCTGGCGTTGTAG-
PTP4A3	GAAGGCCAAGTTCTGTGAGG	TCCAGGTAGGTGAGCTGCTT
PC	TGGAGAAGGAGCTGGTAGAC	GGCAGTGAAGTCCTTGAAGT
RARRES3	ACCAGACCTCCTCTTGGC	GAAGGGGCAGATGGCTGT

Table S2. Sequences of PC si-RNAs

	Sense (5’-3’)	Antisense (5’-3’)
PC-siRNA1	GCAGAUGAAGCCUAUCUCAdTdT	UGAGAUAGGCUUCAUCUGCdTdT
PC-siRNA2	GGAACAUCCUGCACCUGUAdTdT	UACAGGUGCAGGAUGUUCCdTdT
Negative control	UUCUCCGAACGUGUCACGUdTdT	ACGUGACACGUUCGGAGAAdTdT

Table S3. Primers used for ChIP RT-PCR assay

PC promoter	Sense (5’-3’)	Antisense (5’-3’)
PC4-1	TCTTCTCCGCTAGCCAGCGCGTATC	CGATTCATTCACCCGAGTAGCAC
PC4-2	AACGTCCCCACCCCCGCCGACAGCC	ACCACCGCCTACTCCCTTGTCTCC
PC4-3	GCGCCAGGGGCGGGCTCTCCCAGCC	TCTGGACTCCTCCCTGTGCCCGCCT
PC4-4	GCCTAGGGGCGAGGAGTGACGGAGA	AGAGAACAGCTGCGGTCTCCGTGCA

Table S4. Clinicopathological and molecular factors associated with ETE in PTC in FUSCC cohort

	Univariate analysis			Multivariate analysis		
Variables	*P*-value	OR	95% CI for OR	*P*-value	OR	95% CI for OR
Female	0.751	1.172	0.439-3.127			
Age≥55 years	0.632	1.326	0.418-4.205			
Multifocality	0.083	2.179	0.903-5.256			
LNM	0.134	2.269	0.778-6.618			
$BRAF^{V600E}$	0.042*	0.4	0.166-0.968	0.031*	0.353	0.137-0.910
PRDM16 low vs high expression	0.020*	2.921	1.186-7.192	0.036*	2.914	1.074-7.911

Note: *Statistically significant.

Abbreviations: PRDM16, positive regulatory domain containing 16; PTC: papillary thyroid cancer; FUSCC: Fudan University Shanghai Cancer Center; LNM, lymph node metastasis; OR, odd ratio.

Table S5. Clinicopathological and molecular factors associated with ETE in PTC in TCGA cohort

	Univariate analysis			Multivariate analysis		
Variables	*P*-value	OR	95% CI for OR	*P*-value	OR	95% CI for OR
Female	0.784	1.076	0.636-1.820			
Age≥55 years	0.023*	1.742	1.079-2.813	0.009*	2.219	1.224-4.025
Multifocality	<0.001*	2.907	1.618-5.222	0.001*	3.139	1.577-6.250
LNM	<0.001*	2.719	1.657-4.462	0.002*	2.451	1.381-4.347
$BRAF^{V600E}$	<0.001*	4.068	2.354-7.030	0.001*	2.812	1.505-5.254
PRDM16 low vs high expression	<0.001*	3.874	2.347-6.394	0.001*	2.772	1.535-5.007

Note: *Statistically significant.

Abbreviations: PRDM16, positive regulatory domain containing 16; PTC: papillary thyroid cancer; FUSCC: Fudan University Shanghai Cancer Center; LNM, lymph node metastasis; OR, odd ratio.

Table S6. Correlation of PC expression with clinicopathological characteristics in PTC of the TCGA cohort

Variables		Low (n=191)	High (n=191)	*P*-value
Gender	Female	139(48.80%)	146(51.20%)	0.411
	Male	52(53.60%)	45(46.40%)	
Age at diagnosis（years）	<55	128(49.80%)	129(50.20%)	0.913
	⩾55	63(50.4%)	62(49.6%)	
ETE	YES	34(32.7%)	70(67.3%)	<0.001*
	NO	139(55.4%)	112(44.6%)	
Histological Type Name	Classical	116(43.1%)	153(56.9%)	<0.001*
	Follicular	67(80.7%)	16(19.3%)	
	High risk subtypes (Tall cell, sclerosing, columlar)	8(26.7%)	22(73.3%)	
Multifocality	Unifocal	160(51.1%)	153(48.9%)	0.397
	Multifocal	29(45.31%)	35(54.69%)	
T stage	T1-T2	128(53.3%)	112(46.7%)	0.09
	T3-T4	63(44.4%)	79(55.6%)	
LNM	N0	104(57.8%)	76(42.2%)	0.001*
	N1	64(39.5%)	98(60.5%)	
Metastasis	M0	180(50.3%)	178(49.7%)	0.338
	M1	7(70.0%)	3(30.0%)	
8th AJCC TNM stage	I-II	159(51.8%)	147(48.2%)	0.161
	III-IV	33(42.9%)	44(57.1%)	
$BRAF^{V600E}$	Wildtype	131(84.5%)	24(15.5%)	<0.001*
	Mutation	60(26.4%)	167(73.6%)	

Note: *Statistically significant.

Abbreviations: PC, pyruvate carboxylase; PTC: papillary thyroid cancer; TCGA: The Cancer Genomics Atlas; ETE, extrathyroidal extension; LNM, lymph node metastasis; TNM: tumor–node–metastasis.

Table S7. Sequences of PC promoter

PC promoter	Sequences
**PC-1**	GTCAGATCTCTCCAGGCTCAATAAACAATTTCTAGTTAGCAAGATGTTCT<br>CATAGTCTTTGATTCTCCTTTTTGGAAGCAGGTTATTTTTTCTCTTTGCC<br>CTTATCACCATCTGATGTACTATGTATTAATCTGGGCCGGGCGCGGTGGC<br>TCACGCCTATAATCCCAGCACTTTGGGAGGCCGAGGCGGGTGGATCATGA<br>GGTCAGGAGATCGAGACCATCCTGGCTAACAAGGTGAAACCCCGTCTCTA<br>CTAAAAATACAAAAAATTAGCCGGGCGCGGTGGCGGGCGCCTGTAGTCCC<br>AGCTACTCAGGAGGCTGAGGCAGGAGAATGGCGTGAACCCGGGAAGCGGA<br>GCTTGCAGTGAGCCGAGATTGCGCCACTGCAGTCCGCAGTCCGGCCTGGG<br>CGACAGAGCGAGACTCCGTCTCAAAAAAAAAAAAAAAAAAATATATATAT<br>ATATATATATATTAATCTGTCTGCTCTCCTTTGAAGGCAGGATTTTGGTT
**PC-2**	TGTTTGGTCACTCCCTTATCCCCTGGTTGGCACAGACCCCAGTTTACGCA<br>GCGGTGAATGAAAGTAAAAATCTCGGGACCCCCCCCAGCGCCCCAATTC<br>ATGCGAAAGTGAAGATTAAGCCTTGAAGCTGAGTCACTGCAACACCCTCT<br>TCCAAAAGAATAGCTGTTACTAGCATTAGGCATCAGCCATATCCACACGG<br>AAAGGTAAAAACCTCAGGCATTTGGGAAGGGCTGCCCCCACAGATCATTC<br>ACAAGTAAATTCTTGCTGGCCTCCCATAAACAAGGACAGGTCAATTGTAG<br>CTTCAGGTCTACAATCTAAGTCTAGCTCCTAAAAATTCCACAATAAGAAT<br>GTCATTACAAGCTTATTTTCCCAGGTGCAGAACAAAGTCAAAACTACCTA<br>CTCAGAGACATCTGCATAACTGACTCTTCCTTTACTCTCTTTTTCTCTTC<br>AGACATTCATCTCATCTTACGTAGATTTACCGGGCATTAGCTAAAGTCCC
**PC-3**	ATAGGAATGTAACTATTGTCCTAATCGCCTACTTGCCCTTCTTCCTACAT<br>GCCTTCCCCTCATTCCCCCTTTAAGGAAATGCATAAATACTAAACCTCCC<br>GAAAACCTCTTCGGAAAAACAACCACGGATGTGTCTGTGGTTCGTGTTTT<br>TCCCGAGCACGCCCTCAAACTGGATTAATAAGCCTCGATGACAGAGACTT<br>ATGCCTCAGTCACTTGTTCCACTTGTCAAAGCTTTCCTCTTTGACAAGTG<br>TCTTGGGTCTTGTCAAGTGTCTTGTGTCTCAAACAAGGCTTCCAAACCCC<br>CTAGGTCCTGAGTAGCTTCGCTCTGCAGATGACAAAACCGAGACTCAGAG<br>GTTAAGTTGCTTGCCCAAGGTCACACAGACGTACTGTAGACCCCCATCAT<br>CATCTACGCAGCTGTTAAAGGACATAGTCATGTAACCCGTGTGGCACAAG<br>CGCCTTCTCCTTCCTCCTGCCCTTCGCTAACGTTAGCTTTTCCTGCGACC
**PC-4-1**	TCTTCTCCGCTAGCCAGCGCGTATCTGCGTCTAGCCGGGATGCCTGAGCCCC<br>AGTGCGAACTGTTGTGCTTGCAGCTTCGGGAGACACGTGCTACTCGGGTGA<br>ATGAATCG
**PC-4-2**	AACGTCCCCACCCCCGCCGACAGCCAATAACTGCGAGCCACAGCCCGGCCA<br>CTTCCGCCTATTGCGGGCGTCGGCTAGGGTCCGGCGGCCCACGTGAGGCTCC<br>GGAGACAAGGGAGTAGGCGGTGGT

**PC-4-3**	GCGCCAGGGGCGGGCTCTCCCAGCCTCGCCACTTATCCAGGCGCTCGCCGG<br>GGACGGGAGGGGCGGGGCTGACGTGGGGCGCCAAGGCTTAAACGTGACGG<br>ACAGGCGGGCACAGGGAGGAGTCCAGA
**PC-4-4**	GCCTAGGGGCGAGGAGTGACGGAGAACACTGCCCAATAACGGGAGGGGTT<br>GGGCTGTCTGGGCCAATAGGAAGTCCGTAAGGCGGGGCCGGGACTGCAGCA<br>AGTTCGGTTGCACGGAGACCGCAGCTGTTCTCT
